# *De Novo* Missense Substitutions in the Gene Encoding CDK8, a Regulator of the Mediator Complex, Cause a Syndromic Developmental Disorder

**DOI:** 10.1016/j.ajhg.2019.02.006

**Published:** 2019-03-21

**Authors:** Eduardo Calpena, Alexia Hervieu, Teresa Kaserer, Sigrid M.A. Swagemakers, Jacqueline A.C. Goos, Olajumoke Popoola, Maria Jesus Ortiz-Ruiz, Tina Barbaro-Dieber, Lucy Bownass, Eva H. Brilstra, Elise Brimble, Nicola Foulds, Theresa A. Grebe, Aster V.E. Harder, Melissa M. Lees, Kristin G. Monaghan, Ruth A. Newbury-Ecob, Kai-Ren Ong, Deborah Osio, Francis Jeshira Reynoso Santos, Maura R.Z. Ruzhnikov, Aida Telegrafi, Ellen van Binsbergen, Marieke F. van Dooren, The Deciphering Developmental Disorders Study, Peter J. van der Spek, Julian Blagg, Stephen R.F. Twigg, Irene M.J. Mathijssen, Paul A. Clarke, Andrew O.M. Wilkie

**Affiliations:** 1 Clinical Genetics Group, MRC Weatherall Institute of Molecular Medicine, University of Oxford, Oxford OX3 9DS, UK; 2 Cancer Research UK Cancer Therapeutics Unit, the Institute of Cancer Research, London SM2 5NG, UK; 3 Department of Pathology and Department of Bioinformatics, Erasmus University Medical Center, University Medical Center Rotterdam, PO Box 2040, 3000 CA, Rotterdam, the Netherlands; 4 Department of Plastic and Reconstructive Surgery, Erasmus MC, University Medical Center Rotterdam, PO Box 2040, 3000 CA, Rotterdam, the Netherlands; 5 Genetics Division, Cook Children’s Medical Center, Fort Worth, TX 76102, USA; 6 Department of Clinical Genetics, University Hospitals Bristol NHS Foundation Trust, St. Michael’s Hospital, Bristol BS2 8EG, UK; 7 Department of Genetics, University Medical Center Utrecht, Utrecht University, 3508 AB Utrecht, the Netherlands; 8 Department of Neurology and Neurological Sciences, Stanford University School of Medicine, Stanford, CA 94305, USA; 9 Wessex Clinical Genetics Services, University Hospital Southampton, Southampton SO16 5YA, UK; 10 Department of Child Health, University of Arizona College of Medicine, Division of Genetics and Metabolism, Phoenix Children’s Hospital, Phoenix, AZ 85016, USA; 11 North Thames Regional Genetics Service, Great Ormond Street Hospital for Children NHS Foundation Trust, London WC1N 3EH, UK; 12 GeneDx, Gaithersburg, MD 20877, USA; 13 Department of Clinical Genetics, Birmingham Women’s and Children’s NHS Foundation Trust, Birmingham B15 2TG, UK; 14 Genetics Division, Joe DiMaggio Children’s Hospital, Hollywood, FL 33021, USA; 15 Charles E. Schmidt College of Medicine, Florida Atlantic University, Hollywood, FL 33021, USA; 16 Department of Clinical Genetics, Erasmus MC University Medical Center Rotterdam, PO Box 2040, 3000 CA, Rotterdam, the Netherlands; 17 Deciphering Developmental Disorders Study, Wellcome Sanger Institute, Cambridge CB10 1SA, UK

**Keywords:** CDK8, kinase, Mediator complex, hypotonia, *de novo* mutation, intellectual disability, behavioral disorder, congenital heart disease, dominant negative, Mediator kinase modulopathy

## Abstract

The Mediator is an evolutionarily conserved, multi-subunit complex that regulates multiple steps of transcription. Mediator activity is regulated by the reversible association of a four-subunit module comprising CDK8 or CDK19 kinases, together with cyclin C, MED12 or MED12L, and MED13 or MED13L. Mutations in *MED12*, *MED13,* and *MED13L* were previously identified in syndromic developmental disorders with overlapping phenotypes. Here, we report *CDK8* mutations (located at 13q12.13) that cause a phenotypically related disorder. Using whole-exome or whole-genome sequencing, and by international collaboration, we identified eight different heterozygous missense *CDK8* substitutions, including 10 shown to have arisen *de novo*, in 12 unrelated subjects; a recurrent mutation, c.185C>T (p.Ser62Leu), was present in five individuals. All predicted substitutions localize to the ATP-binding pocket of the kinase domain. Affected individuals have overlapping phenotypes characterized by hypotonia, mild to moderate intellectual disability, behavioral disorders, and variable facial dysmorphism. Congenital heart disease occurred in six subjects; additional features present in multiple individuals included agenesis of the corpus callosum, ano-rectal malformations, seizures, and hearing or visual impairments. To evaluate the functional impact of the mutations, we measured phosphorylation at STAT1-Ser727, a known CDK8 substrate, in a *CDK8* and *CDK19* CRISPR double-knockout cell line transfected with wild-type (WT) or mutant *CDK8* constructs. These experiments demonstrated a reduction in STAT1 phosphorylation by all mutants, in most cases to a similar extent as in a kinase-dead control. We conclude that missense mutations in *CDK8* cause a developmental disorder that has phenotypic similarity to syndromes associated with mutations in other subunits of the Mediator kinase module, indicating probable overlap in pathogenic mechanisms.

## Main Text

**Figure 1:**
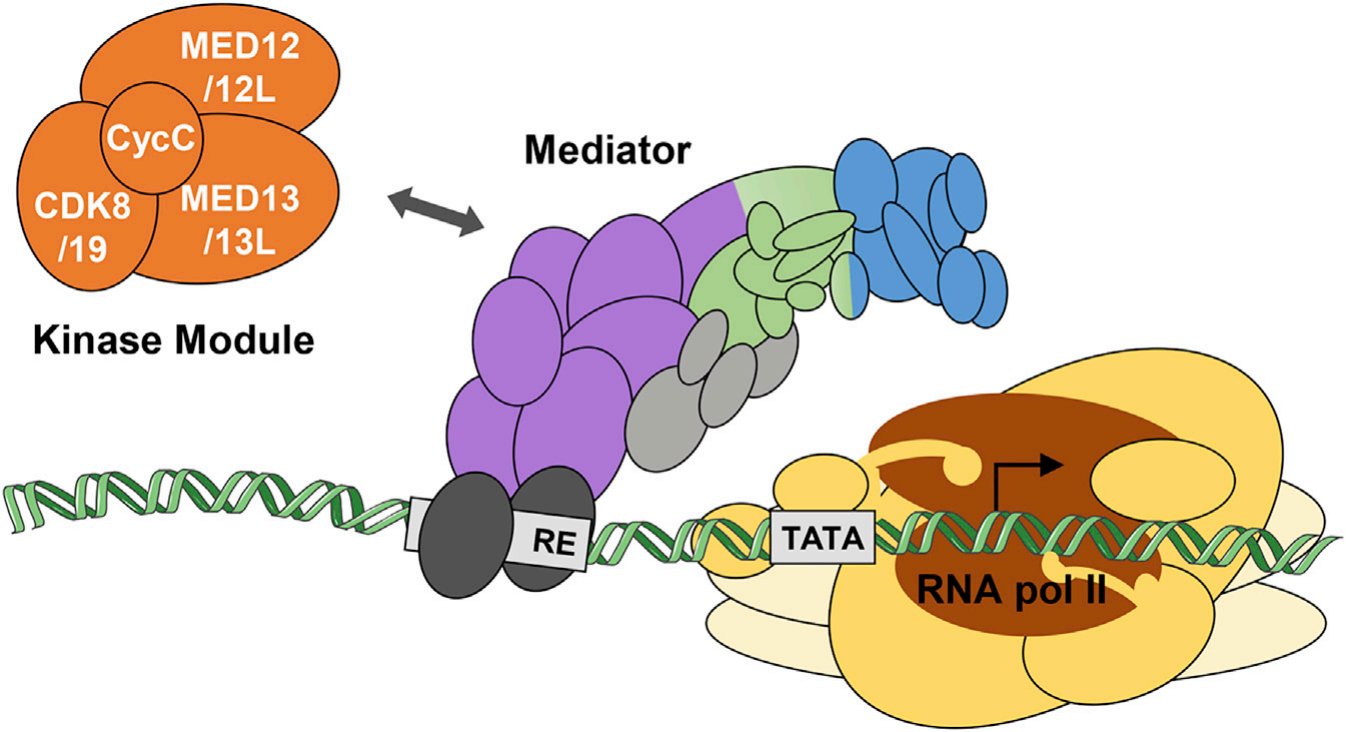
Simplified Illustration of the Mediator Complex and the RNA Pol II Machinery at the Promoter of a Hypothetical Gene The CDK8 or CDK19 kinase Module (orange) reversibly binds the Mediator complex to regulate its activity. The Mediator complex (head in blue, middle in green, and tail in purple; additional subunits in light gray) bridges between gene-specific activators (dark gray) that are bound to regulatory elements (RE) and general transcription machinery that comprises the RNA pol II (brown) and general transcription factors (yellow). The DNA molecule cartoon was obtained from the Servier Medical Art; the representation was adapted from Larivière et al.^3^

The Mediator complex is a large, multi-subunit assembly encoded by 26 genes in humans, and it regulates gene expression in all eukaryotes. The core function of the Mediator is to communicate developmental and physiological signals from DNA-bound transcription factors to RNA polymerase II (the enzyme responsible for the transcription of all protein-coding and most non-coding genes) by contacting the pre-initiation complex.^1^, ^2^ Regulation of Mediator activity is, in part, achieved by the reversible association of a four-subunit kinase module (hereafter referred to as the “Module”) comprising cyclin C (encoded by *CCNC*) and either cyclin-dependent kinase 8 (CDK8), MED12, and MED13 or their respective paralogs CDK19, MED12L, and MED13L (Figure 1). Many targets of phosphorylation by CDK8 have been identified.^4^ These include not only the core Mediator-RNA-polymerase-II complex (Module components [CDK8, cyclin C, MED12, MED13, and MED13L], other Mediator subunits [MED14 and MED26], and the C-terminal domain of RNA polymerase II) but also many other transcriptional regulators (for example, tissue-specific and general transcription factors, pause or release factors, chromatin-remodeling factors, and histone H3; reviewed by Jeronimo and Robert).^2^ Mirroring this, genetic analyses of the Module have uncovered many tissue- and species-specific functions, ranging from environmental responses in yeast to organogenesis and development in the nematode worm, fruit fly, zebrafish, and mouse.^5^

In humans, mutations in the genes (*MED12*, *MED13*, and *MED13L*) encoding subunits of the Module have been implicated in developmental disorders. Mutations in the X-linked *MED12* (MIM: 300188) cause at least three different syndromes: Opitz-Kaveggia syndrome (also known as FG syndrome; MIM: 305450), Lujan-Fryns syndrome (MIM: 309520), and Ohdo syndrome (MIM: 300895). These syndromes have partly overlapping clinical features, including multiple congenital defects, facial dysmorphic features, hypotonia, behavioral problems, and intellectual disability (ID).^6^ Mutations in *MED13L* (MIM: 608771) are associated with a syndromic form of ID (MIM: 616789) characterized by facial dysmorphism, ID, speech impairment, motor developmental delay with muscular hypotonia, cardiac anomalies, and behavioral difficulties.^7^, ^8^ Recently, mutations in *MED13* (MIM: 603808) have been reported as leading to a neurodevelopmental disorder characterized by ID and/or developmental delay, including speech delay; additional features that were present in two or more affected individuals included autism spectrum disorder (ASD), attention deficit hyperactivity disorder (ADHD), optic nerve abnormalities, Duane anomaly, hypotonia, mild congenital heart abnormalities, and dysmorphic features.^9^ Here, using whole-exome or whole-genome sequencing and by international collaboration, we report *de novo* mutations in *CDK8*, which has not been previously associated with a congenital disorder, in 12 unrelated individuals with overlapping phenotypes.

**Figure 2:**
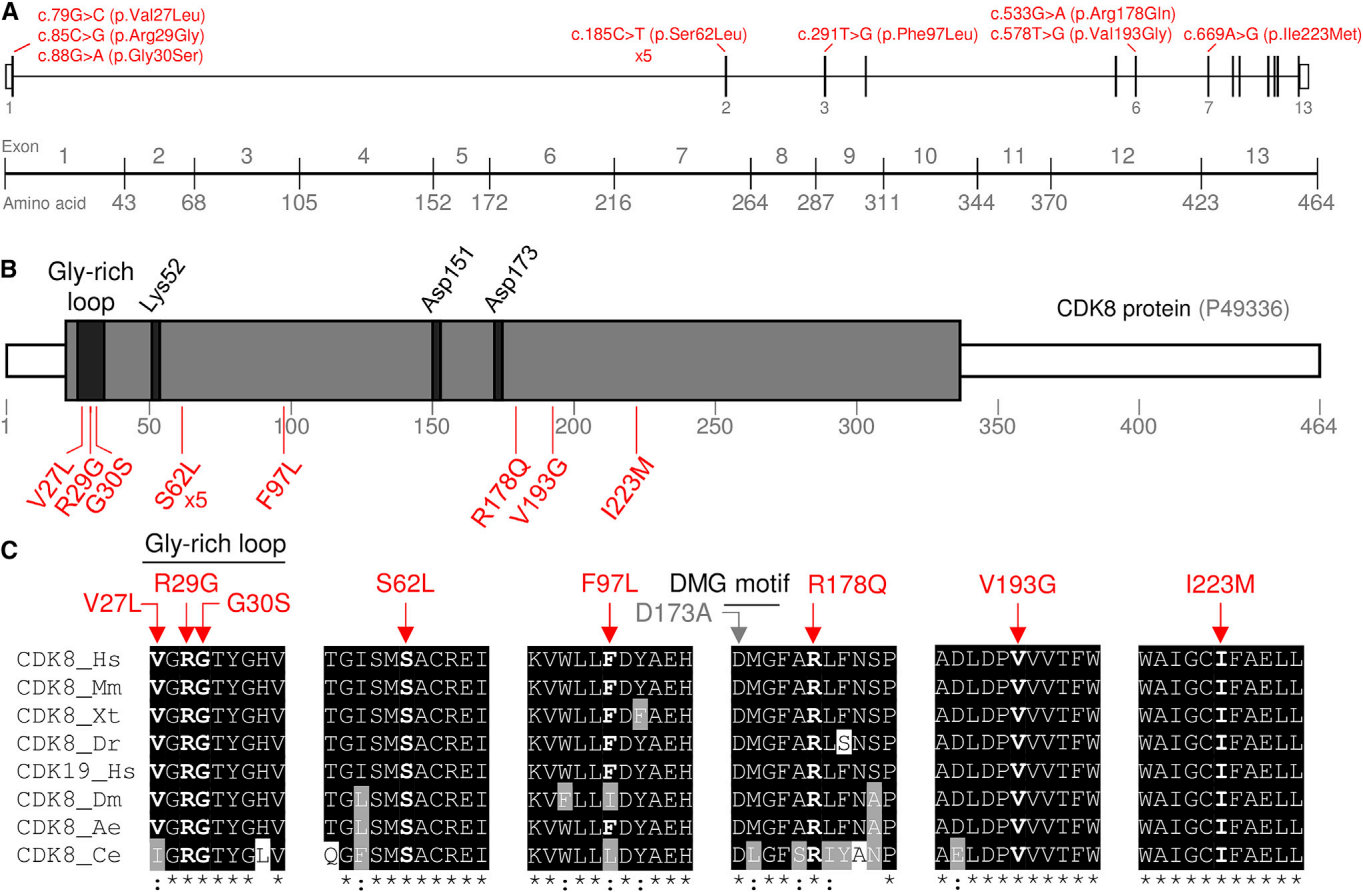
Mutations in *CDK8* and Conservation of Substituted Residues (A) Localization of mutations (in red) in a schematic representation of human *CDK8* (exon numbering on GenBank: NM_001260.2). (B) A cartoon of the human CDK8 (based on UniProt: P49336) showing the location of the protein kinase domain (21–335; gray box) and the eight different CDK8 substitutions identified in this work (below, in red). Note that the p.Ser62Leu substitution was identified in five unrelated subjects. Relevant functional elements are depicted in dark boxes. Note the Gly-rich loop (27–35); Lys52, a catalytic residue that interacts with the triphosphate of ATP in the active site; Asp151 within the conserved His-Arg-Asp (HRD) motif (at the catalytic loop), which is directly involved in catalysis; and Asp173, which is included in the Asp-Met-Gly (DMG) motif (at the activation segment) required for chelation of the magnesium ion involved in the catalysis. Substitution of Asp173 is widely used as a catalytically inactive kinase-dead form of CDK8 (p.Asp173Ala). (C) A multiple-protein sequence alignment of CDK8 and CDK19 at the positions of the identified substitutions in CDK8. Abbreviations are as follows: Hs = human, Mm = mouse, Xt = western clawed frog, Dr = zebrafish, Dm = fruit fly, Ae = yellow fever mosquito, and Ce = nematode worm. The positions of the CDK8 *de novo* missense variants are indicated with a red arrow at the top of the sequences. Black- or gray-shaded amino acids indicate identical or similar residues compared to the human CDK8 sequence, respectively. Below the alignments, an asterisk (^∗^) indicates positions that have a single, fully conserved residue, whereas a colon (:) indicates conservation between groups of similar properties. Additional relevant features present in these sequences (Gly-rich loop, DMG motif, and the Asp173 residue, mutated in the kinase-dead mutant) are also depicted.

The study was initiated as part of a whole-genome sequencing (WGS)-based investigation of craniosynostosis in the Netherlands after approval by the board of the medical ethical committee Rotterdam (MEC-2012-140). Written informed consent was obtained from all participants or their legal guardians. Parent-child trio-based WGS of a proband with metopic synostosis and ID (subject 3 in Table 1) identified a *de novo* c.88G>A transition in *CDK8*; this change leads to the prediction of a heterozygous p.Gly30Ser substitution. The position of this substitution, at an almost invariant residue within the highly conserved glycine-rich loop of the kinase domain,^9^ and in which pathogenic mutations were previously identified in other protein kinases,^22^, ^57^ led us to seek evidence for additional pathogenic variants in *CDK8* through GeneMatcher exchange^10^ and from the Deciphering Developmental Disorders (DDD) research study.^11^ The DDD study has UK research ethics committee (REC) approval (10/H0305/83, granted by the Cambridge South REC, and GEN/284/12 granted by the Republic of Ireland REC). Details of the methodology we used to identify each mutation (whole-exome sequencing [WES] in every instance except the original index subject) are provided in the Supplemental Material and Methods. By these means, we identified a further 11 unrelated individuals harboring eight different nucleotide substitutions within *CDK8*; one substitution (c.185C>T encoding p.Ser62Leu) was present in five subjects (Figure 2A and Table 1). All *CDK8* variants were constitutionally absent from the ExAC and gnomAD databases of common variation (accessed November 2018).^12^

**Table 1:** Clinical Features of Subjects with *CDK8* Mutations

**Subject #**	**1**	**2**	**3**	**4**	**5**	**6**	**7**	**8**	**9**	**10**	**11**	**12**
Gender	f	m	m	f	m	f	m	m	f	m	f	m
Age (years)	9	2.9	8	7.6	0.1	0.8	16.7	12.7	6.8	5.4	12	3.5
Mutation	c.79G>C	c.85C>G	c.88G>A	c.185C>T	c.185C>T	c.185C>T	c.185C>T	c.185C>T	c.291T>G	c.533G>A	c.578T>G	c.669A>G
Substitution	p.Val27Leu	p.Arg29Gly	p.Gly30Ser	p.Ser62Leu	p.Ser62Leu	p.Ser62Leu	p.Ser62Leu	p.Ser62Leu	p.Phe97Leu	p.Arg178Gln	p.Val193Gly	p.Ile223Met
*De novo*	yes	yes	yes	yes	yes	yes	NA	NA	yes	yes	yes	yes
Facial dysmorphism	+	+	+	+	+	+	+	+	−	+	+	+
Hypotonia, motor delay, and/or walking difficulty	+	+ (axial)	+	+	NA	+ (axial)	+ (bilateral *pes planus* surgery)	+	+	+	+	+
Brain MRI or CT	normal	thin corpus callosum	normal	ACC	NA	ACC	NA	ACC	non-specific	NA	normal	NA
Ophthalmic: ptosis	+	−	−	−	−	−	−	−	−	−	+	−
Strabismus	+	+	+	−	−	−	−	+	−	−	−	−
Myopia	+	+	−	+	−	−	+ (severe)	+	−	−	+	−
Impaired vision	+	−	−	−	−	−	+	−	−	−	−	−
Sensorineural hearing loss	moderate	moderate	−	−	NA	−	−	−	severe (unilateral)	NA	(glue ear)	−
Intellectual disability	mild (SEN school)	moderate to severe	moderate to severe (SEN school)	moderate	NA	NA; moderate motor delay	mild to moderate (SEN school)	moderate (SEN school)	moderate	moderate	mild (normal school)	NA; moderate motor delay
Behavioral disorder	ADHD	−	ASD, ADHD, sleep disorder	ADHD, sleep disorder	NA	NA	ASD	ASD, ADHD	ASD	happy disposition	attention seeking, volatile	ASD
Epilepsy	−	−	−	−	−	−	complex partial	−	general and focal	−	−	−
Feeding difficulties	infancy only	gastrostomy-fed	congenital pyloric stenosis	previous gastrostomy	NA	−	reflux	reflux, regurgitation	reflux, episodic vomiting	−	episodic vomiting	−
Congenital heart disease (S indicates age at surgery)	−	−	perimembraneous VSD, double orifice mitral valve	hypoplastic left heart (S: 4 days, 3 months, 4 years)	atrial septal defect, VSD, bicuspid aortic valve, hypoplastic aortic arch (S: unknown)	coarctation of the aorta, subaortic stenosis, mitral stenosis (S: 5 months)	tetralogy of Fallot (S: 9 months)	−	−	−	−	VSD, PFO
Other			metopic synostosis		anterior anus, recto-perianal fistula		undescended testes	rectal mucosal prolapse				

Abbreviations and symbols are as follows: + = present; − = absent; NA = information not available; f = female; m = male; ACC = agenesis of the corpus callosum; ADHD = attention deficit-hyperactivity disorder; ASD = autism spectrum disorder; PFO = patent foramen ovale; SEN = special educational needs; and VSD = ventricular septal defect.

*CDK8* is a gene that comprises 13 exons; it is located at chromosomal region 13q12.13 and encodes a major isoform of 464 amino acids. All seven unique substitutions, and three of five instances of p.Ser62Leu, arose *de novo* from unaffected parents who had correct paternity confirmed either by comparison of trio-WES or WGS data or by microsatellite analysis; paternal samples were unavailable in the remaining two cases (the recurrence of the c.185C>T transition is likely to be explained by its position in a hypermutable CpG dinucleotide). The eight substituted amino acids are all located within the kinase domain, which extends between amino acids 21–335 (Figure 2B), and they are invariant in a wide range of vertebrate CDK8 orthologs, as well as in the human paralog CDK19; in six cases, this invariance extends to the invertebrates *Drosophila, Aedes*, and *Caenorhabditis* (Figure 2C). MutationTaster predicted all eight substitutions to be disease-causing, and SIFT and PolyPhen-2 predicted all to be damaging with the exception of the c.291T>G (p.Phe97Leu) variant (Table S1). *CDK8* is intolerant to missense variants (z = 3.81, observed/expected ratio = 0.34; gnomAD database)^12^ and exhibits high constraint throughout the protein.^13^ On the basis of the DOMINO algorithm, *CDK8* was predicted to be among the top candidate genes for which mutations are likely to manifest with a dominant pattern of inheritance.^14^ The clustering of multiple *de novo* substitutions localized to a single, highly conserved domain of the protein provides a strong pathogenicity signature, as has been described for several other genes including the cyclin-dependent kinase (CDK)-encoding *CDK13*.^11^, ^15^, ^16^

**Figure 3:**
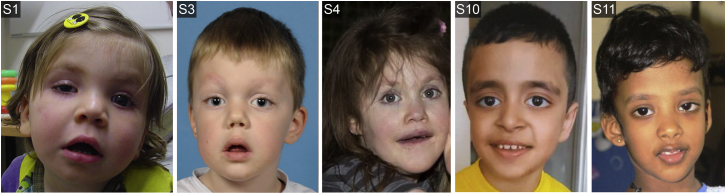
Clinical Pictures of Subjects with *CDK8* Mutations Subjects (left to right): 1 (c.79G>C [p.Val27Leu]) aged 16 months, 3 (c.88G>A [p.Gly30Ser]) aged 4 years (postoperative after surgery to correct metopic synostosis), 4 (c.185C>T [p.Ser62Leu]) aged 9 years, 10 (c.533G>A [p.Arg178Gln]) aged 9 years, and 11 (c.578T>G [p.Val193Gly]) aged 2.5 years.

We assessed the phenotypes of the 12 subjects, seven males and five females aged 0.1–16.7 years at the last clinical assessment, with CDK8 substitutions by using a standardized spreadsheet-based questionnaire (summarized in Table 1; full details can be found in Table S2, and case reports can be found in the Supplemental Note). Most affected children were born after unremarkable pregnancies, at or near term, with a birth weight within the normal range, and they did not require neonatal resuscitation, although around half required hospitalization after birth because of jaundice, seizures, laryngomalacia, or cardiac problems. More consistent difficulties emerged in early infancy: hypotonia (noted in 11/12 subjects) was frequently evident, and it later manifested as motor delay and sometimes persistent problems in walking. Mild to moderate developmental delay was universal, and older children had ID which ranged from mild (two cases) to moderate–severe (two cases); most were in the moderate range (five cases). Most children attended schools for special educational needs, but one had mainstream education. Behavioral symptoms were prominent: seven of ten of the older individuals had formal diagnoses of autism spectrum disorder (ASD) and/or attention deficit hyperactivity disorder (ADHD). The head circumference was normal except in one subject, who had mild macrocephaly (+2.21 standard deviations [SD]); magnetic resonance imaging (MRI) of the brain showed agenesis or thinning of the corpus callosum in four subjects, including the macrocephalic individual. Two individuals had seizures, but brain imaging was either normal or was not available. Three individuals had moderate-severe sensorineural hearing loss; ophthalmological abnormalities were frequent, including myopia (n = 6), eyelid ptosis (n = 2), and/or strabismus (n = 4), and these were associated with marked visual impairment in two children. Congenital heart defects (CHDs) were present in six of the twelve subjects; the defects were classified^17^ as left ventricular obstruction (n = 3), conotruncal defects (n = 1), and other (n = 2). Present congenital gastrointestinal problems were ano-rectal abnormalities (n = 2) and pyloric stenosis (n = 1); two additional infants required feeding via gastrostomy tube. In later childhood, gastro-esophageal reflux or episodic vomiting were significant management issues in four individuals. Facial dysmorphism was identified in 11 subjects; this was not characteristic, although arched eyebrows, epicanthic folds, prominent eyes, a prominent nasal tip or long columella, a long philtrum, wide or open-mouth posture, prognathism, and low set and prominent ears were highlighted in two or more subjects (Figure 3 and Table S2). The subjects’ stature was within the normal range except in one individual, who had mild short stature (−2.53 SD). Apart from the metopic synostosis present in the index individual, no other individuals had craniosynostosis.

**Figure 4:**
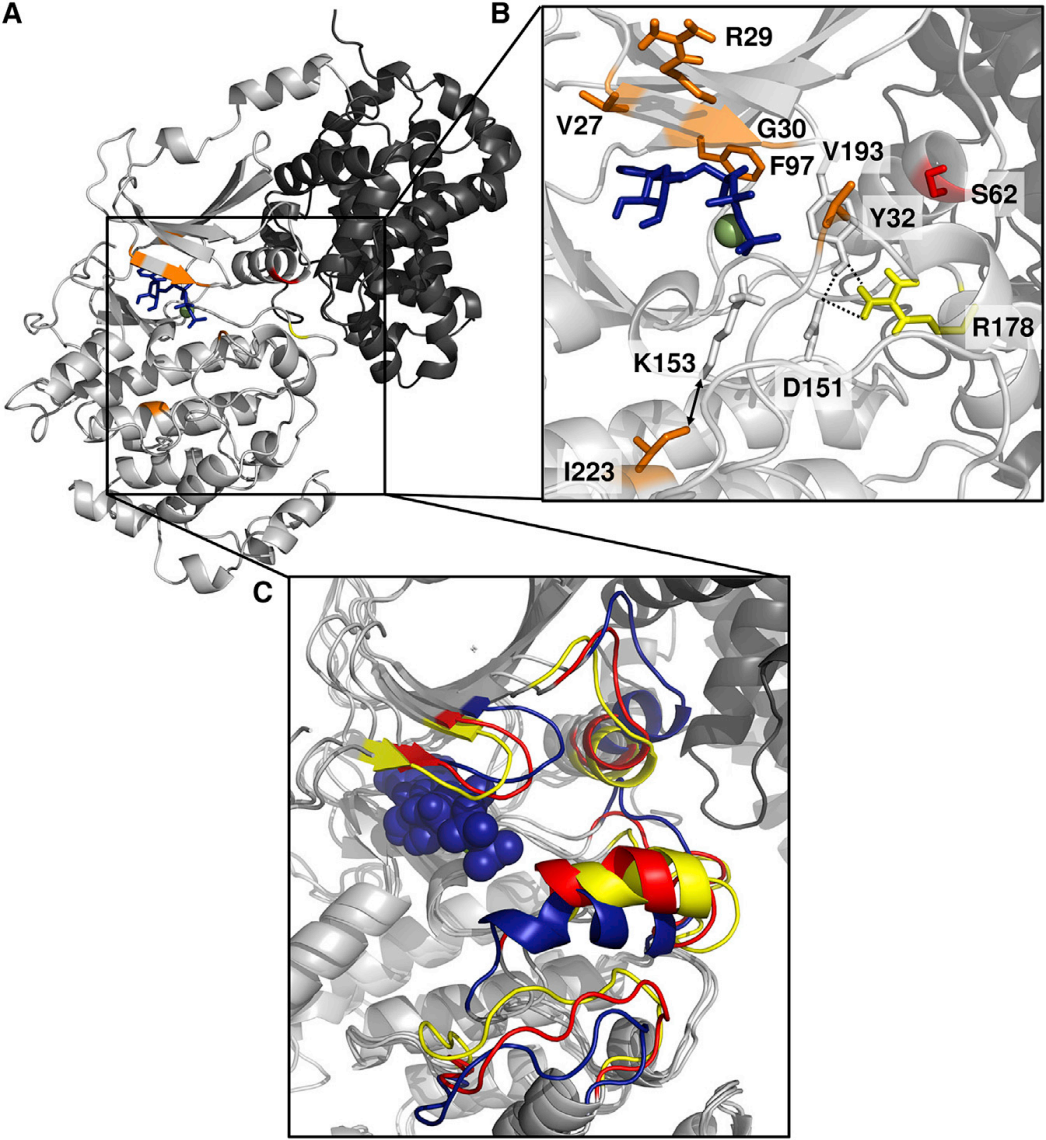
Location and Effect of CDK8 Substitutions on the Protein Structure (A) Overall structure of CDK8 (light gray) in complex with cyclin C (dark gray). ATP (blue) was modeled into the CDK8 DMG-in crystal structure (PDB: 4F7S)^18^ using aligned CDK9 (PDB 3BLQ).^19^ Regions not previously resolved in the crystal structure have been modeled using Molecular Operating Environment (MOE).^20^ (B) A magnified view of the catalytic region of the CDK8 kinase domain. The Mg^2+^ is shown as a green sphere. Amino acids at which mutations have been identified are colored in orange (except Ser62 in red and Arg178 in yellow), and relevant, potential interaction partners in the input structure are shown as sticks. Ile223 is in close proximity (double-headed arrow) to the Lys153 involved in interaction with ATP-phosphate. Arg178 forms a hydrogen bond with Tyr32 (Gly-rich loop) and interacts ionically with the catalytic Asp151. (C) A magnified overlay of the wild-type (WT) and mutant molecular-dynamics simulations of CDK8 (light gray) in complex with cyclin C (dark gray) in the presence of ATP (blue spheres). The areas displaying the most pronounced structural differences within the substrate binding site are colored (blue: WT, red: p.Ser62Leu, and yellow: p.Arg178Gln).

We assessed the probable effects of the eight individual amino acid substitutions on the CDK8 kinase domain by examining available protein structures (Figure 4). Three of the substitutions (c.79G>C [p.Val27Leu], c.85C>G [p.Arg29Gly], and p.Gly30Ser) are clustered in the Gly-rich loop (amino acids 27–35), a highly conserved protein-kinase motif with a critical role in ATP binding and in the phosphoryl-transfer reaction.^21^, ^22^, ^23^ Substitutions within this motif affect kinase activity and, in other kinases, have been described as pathogenic mutations responsible for various congenital disorders.^15^, ^22^, ^24^, ^25^ Within the three-dimensional structure, all CDK8 substitutions either affect amino acids surrounding the ATP-binding pocket (Figure 4B) or are in contact with key functional amino acids; for example, the p.Ile223 residue is not directly part of the ATP-binding site but is within interacting distance of p.Lys153, which directly binds to the ATP phosphate in the input structural model. The recurrently mutated p.Ser62 residue is located in the αC-helix,^26^ the conformation and interactions of which are important for CDK8 activity.^27^ Two substitutions, c.533G>A (p.Arg178Gln) and c.578T>G (p.Val193Gly), affect amino acids located in the activation segment,^26^ which interacts with other key amino acids (Figure 4B). One of the substitutions (p.Phe97Leu) affects the “gatekeeper” p.Phe97 of CDK8. Mutations in the gatekeeper residue of kinases have emerged as a key mechanism by which cancer cells develop resistance to treatment,^28^ highlighting the importance of the gatekeeper residue controlling the back cavity of the ATP site.^29^

To gain further insights into the effects of the mutations, we conducted molecular-dynamics simulations for wild-type (WT) and two selected CDK8 substitutions, p.Ser62Leu, and p.Arg178Gln, in complex with cyclin C and in the presence of ATP. The overall CDK8 protein structure and organization was predicted to be unchanged in the mutant models compared to in the WT. However, we found that in addition to impacting the ATP-binding site, the mutants were also predicted to induce pronounced structural changes in the substrate-binding site (Figure 4C). In particular, the substrate-binding site of the WT CDK8 protein can adopt more open conformations than its mutated counterparts, potentially allowing substrates to bind more closely in the vicinity of ATP in the WT protein.

**Figure 5:**
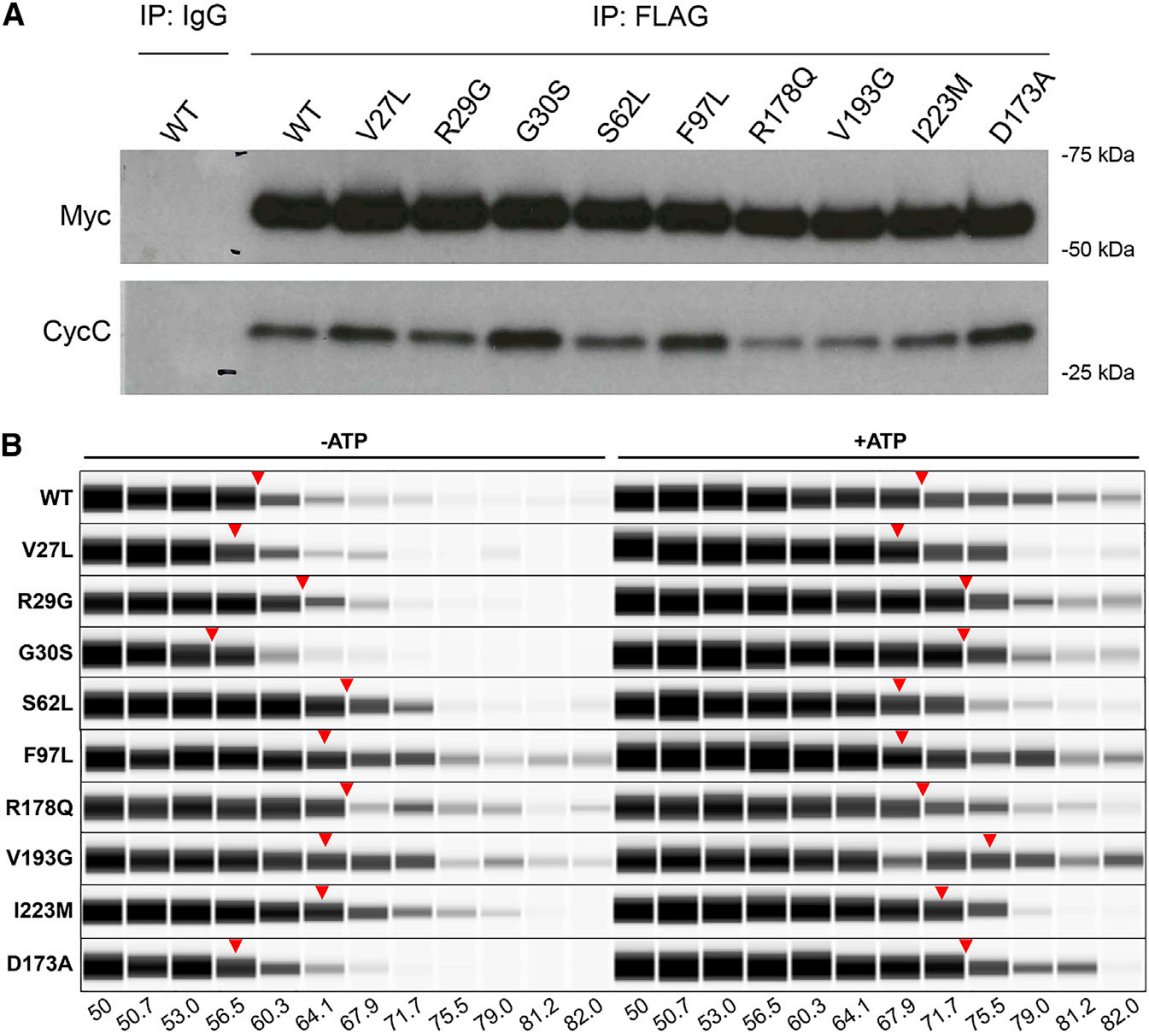
Immunoprecipitation of CDK8 and Thermal Stability Assay (A) Lysates from HEK293T cells transiently transfected with Myc-FLAG-tagged wild-type (WT) or mutant *CDK8* constructs were used for immunoprecipitation with anti-FLAG antibody or mouse IgG isotype control antibody. The immunocomplexes were purified with protein G magnetic beads. CDK8 was released by adding FLAG peptide, and an aliquot of the eluted fraction was saved for immunoblot analysis with anti-Myc or anti-cyclin C (CycC) antibodies. (B) A thermal stability assay of the eluted fractions incubated in the absence or presence of ATP and heated individually at different temperatures (gradient between 50–82°C). Virtual blot views of results for WT and the mutants are shown. For each CDK8 construct, the red arrowhead indicates 50% reduction of the CDK8 signal compared to the signal at the lowest temperature.

To determine whether the CDK8 kinase domain substitutions had caused structural changes and/or affected the ability to bind ATP, we purified CDK8 kinase Modules that were isolated from cells by immunoprecipitation after the expression of WT or mutant-tagged-CDK8 (Figures 5A and S1), and we recorded protein-melting curves in the absence or presence of ATP (Figure 5B). In the absence of ATP, the thermal stability of mutant proteins ranged from indistinguishable to increased in comparison to the WT protein. Moreover, the stability of most mutant proteins was increased by the addition of ATP, in some cases to a greater extent than occurred in the WT protein. Together these results suggest that none of the substitutions cause gross mis-folding of the protein, and most or all are still able to bind ATP.

**Figure 6:**
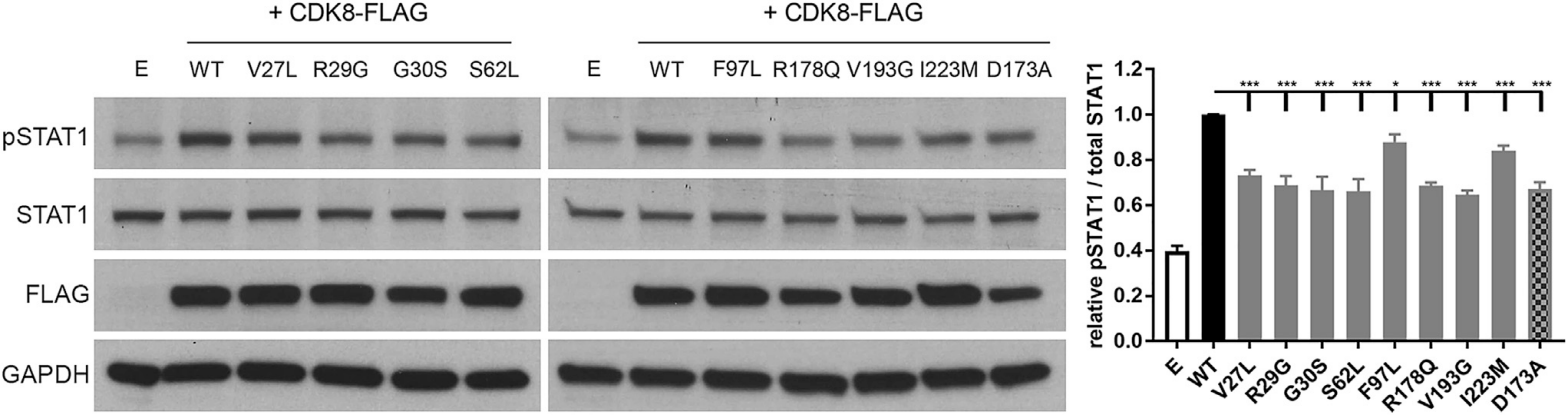
Effect of CDK8 Substitutions on STAT1-Ser727 Phosphorylation SW620 *CDK8* and *CDK19* double-knockout cells were transiently transfected with FLAG-tagged wild-type (WT) or mutant *CDK8* constructs, and the cellular levels of pSTAT1-Ser727 were determined as a readout of CDK8 activity. The CDK8 kinase-dead (p.Asp173Ala) mutant was used as a control for defective kinase activity. Whole-cell lysates from non-transfected cells (E lane) or from transfected cells were collected, and equal amounts of total protein were subjected to immunoblotting with anti-phospho-STAT1-Ser727 and anti-STAT1 (total) antibodies. Anti-FLAG was used to detect the overexpressed CDK8 constructs, and GAPDH was used as loading control. Representative blots are shown in the two left panels. For the quantification (right panel), the relative intensities of the bands were taken as a ratio of the phosphorylated STAT1-Ser727 (pSTAT1) over the total amount of STAT1 (STAT1) and then plotted against the WT, which was normalized to one, for each individual blot. The values shown represent means ± SEM from five independent experiments. The results were compared to WT and analyzed by one-way ANOVA with Dunnett’s multiple-comparisons test; ^∗^ p ≤ 0.05, ^∗∗^ p ≤ 0.01, and ^∗∗∗^ p ≤ 0.001.

Finally, we investigated how the CDK8 substitutions affected the kinase activity by measuring phosphorylation of one of its well-validated targets: p.Ser727, of the human transcription factor STAT1.^30^, ^31^, ^32^, ^33^, ^34^ We used a CRISPR-Cas9-engineered human cell line (SW620 colorectal carcinoma cells) that lacks both CDK8 and CDK19 (M.J. Ortiz-Ruiz et al., Cancer Res., abstract). These cells exhibited baseline STAT1-Ser727 (pSTAT1) phosphorylation that was substantially increased when WT CDK8 was transiently re-expressed (Figure 6). We performed site-directed mutagenesis to introduce the eight observed variants into *CDK8* cDNA (Supplemental Material and Methods), which we transfected into the *CDK8* and *CDK19* CRISPR double-knockout cells. We observed that the cellular levels of pSTAT1 were reduced to a statistically significant extent, compared to WT, in all mutant-transfected cells (Figure 6). For most mutants, the reduction in kinase activity was similar to that in the catalytically inactive CDK8 kinase-dead (p.Asp173Ala) mutant we used as a control, but in two mutants (p.Phe97Leu and [c.669A>G] p.Ile223Met), an intermediate level of phosphorylation (∼63% and ∼51% in comparison to the WT, respectively), was observed.

Collectively, our genetic and functional assessments provide strong evidence for a previously unrecognized (to our knowledge) clinical disorder caused by heterozygous missense substitutions located in the kinase domain of CDK8. All eight distinct mutations were shown to have arisen *de novo* from unaffected parents (Table 1) and exhibited a similar combination of molecular characteristics. All clustered within the kinase domain (Figure 2) and were concentrated around the ATP binding pocket (Figure 4); none was associated with gross protein instability, and all retained the ability to bind ATP (Figure 5). Finally, all exhibited attenuated kinase activity in a previously validated assay of STAT1-phosphorylation, although the magnitude of the effect was diminished for two substitutions (Figure 6), suggesting partial retention of activity in those cases. Of note, the CDK8 mutant proteins maintained their ability to bind cyclin C, similar to the ability of the kinase-dead mutant (Figures 5A and S1, and as previously described).^35^ The CDK8 kinase-dead mutant was previously shown to form a stable Module, although the inactive kinase was unable to phosphorylate its targets.^35^, ^36^

Concordant with these molecular observations was a relatively consistent phenotypic presentation (Figure 3, Table 1, Supplemental Case Reports, and Table S2). Hypotonia was usually evident in infancy and, in children old enough for assessment, learning disability was universal and most commonly classified as moderate; most but not all children were attending schools for special educational needs. Associated behavioral problems, which were frequent and added to the management issues for these children, included formal diagnoses of ASD and ADHD, sleep disorders, and episodic vomiting. Dysmorphic facial features, including arched eyebrows, a bulbous or upturned nose, and hypotonic facies, were frequent; however, these do not constitute a consistent or easily recognizable phenotype. Six of 12 subjects had congenital heart malformations, including at least three requiring corrective surgery. Additional medically significant features are documented in Table 1. The high frequency of diagnosis of ASD, ADHD, and CHD in these children is of note because *CDK8* mutations have not previously been highlighted in genetic screens of those disorders;^17^, ^37^ this might be because the target size for mutations of *CDK8* (missense substitutions surrounding the ATP-binding pocket) is relatively small. Although the mutation in our index subject, who had metopic synostosis, was originally identified in a genetic study of craniosynostosis, none of the 11 other subjects identified had this phenotype. It is unclear whether the co-occurrence of the mutation and craniosynostosis is causally linked (potentially through the adverse effect of the mutation on brain development)^38^ or is coincidental.

In more than two decades since *CDK8* was first identified in independent studies of the yeast *Saccharomyces cerevisiae* (from screens for the stabilization of meiotic mRNAs [*Ume5*]^39^ and suppression of an RNA polymerase II C-terminal domain mutation [*Srb10*]),^40^ a large literature has accumulated about its functions.^5^, ^41^ As described above and illustrated in Figure 1, *CDK8* encodes a kinase component of the Module, a four-subunit complex that binds to the Mediator and regulates its activity. Many fundamental uncertainties remain about its function, for example how the association and dissociation of the Module with the Mediator is regulated, how the Module regulates gene expression both positively and negatively, and the extent to which kinase activity *per se* is required for these functions.^2^ Previous studies of human *CDK8* have mostly focused on its potential as a therapeutic target in cancer, on the basis of the observation that amplifications of *CDK8* are frequent in colon cancer.^42^ Although effective CDK8 inhibitors have been developed, to date they have exhibited unacceptable toxicity.^31^, ^32^ This toxicity might reflect the essential function of CDK8 for normal development, as shown by the pre-implantation lethality of homozygous-null *Cdk8* mutations in the mouse; importantly in relation to the discussion below, heterozygous animals were reported to be normal and fertile.^43^

If one focuses on the developmental role of CDK8, our observations show that although a single WT *CDK8* allele is sufficient for survival, heterozygosity for a mutant allele that encodes a missense substitution of the kinase domain causes pleiotropic developmental abnormalities. Broadly speaking, heterozygous mutations might be associated with abnormal phenotypes through one of three mechanisms: haploinsufficiency, gain of function, and dominant-negative activity.^44^ When haploinsufficiency occurs, many mutations are expected to be either truncating or partial or whole-gene deletions. Deletions involving *CDK8* are very rare, and those recorded involve many other genes, so they are essentially uninformative. Although our gene-matching methods would not detect deletions, they were unbiased with regard to the nature of the intragenic mutation. It was previously reported that 381 of 706 *de novo* mutations in haploinsufficient genes were truncating,^11^ so the finding that 11/11 (excluding the index subject) intragenic nucleotide substitutions encode missense changes diverges significantly from a haploinsufficiency pattern (p = 0.0004). Moreover, human *CDK8* is only moderately constrained to loss of function (probability that the gene is intolerant to a loss of function (LoF) mutation (pLI) = 0.38, gnomAD database); it has six (out of ∼250,000) bona fide truncating alleles listed in gnomAD, suggesting that such alleles exist at low frequency in the normal population. Overall, haploinsufficiency appears unlikely to be the underlying mechanism that explains the *CDK8*-mutation-associated phenotype.

Instead, our experimental observations indicate that the mutations retain ATP binding yet lose or diminish the ability to phosphorylate a well-established substrate, STAT1. This might result from reduced ATP catalysis and phosphoryl-transfer and/or altered substrate binding resulting from reduced accessibility of the substrate-binding site predicted by the dynamic modeling (Figure 4). Notably, CDK8 (together with its paralog CDK19) are the only components of the Module with catalytic activity, so we predict that up to 50% of CDK8 modules could be present in a kinase-inactive, non-productive state. The consequence of this would then depend on the extent to which supply of the catalytically-active Module was rate-limiting to key cellular processes in particular developmental contexts. The notion that the supply of active Modules might frequently be limiting is supported by the observation that all six other genes encoding components of the human Module (*CCNC*, *CDK19*, *MED12*, *MED12L*, *MED13*, and *MED13L*) exhibit very strong constraint against loss-of-function variation (pLI = 1, observed/expected scores 0–0.11), indicating that reduction of any Module component by 50% impairs survival (by contrast, only three of 26 genes encoding other components of the Mediator exhibit pLI = 1; Table S3). Together, these observations currently support a dominant-negative mechanism of pathogenesis for CDK8 substitutions. Similar patterns of localized missense substitutions have been highlighted as a signature of dominant-negative activity, including in the kinase domain of another cyclin-dependent kinase, CDK13.^15^, ^16^ Moreover, overexpression of the kinase-dead mutants of different CDKs (CDK1, CDK2, CDK3, or CDK9) in human cells was previously observed to act by dominant-negative mechanisms.^45^, ^46^, ^47^ We explored the mechanism directly by mixing equal amounts of the WT and p.Ser62Leu CDK8 constructs and measuring STAT1 phosphorylation, but the observed reduction in phosphorylation in mixed samples did not obviously deviate from a linear response (data not shown); hence, further experiments will be required to support or refute the dominant-negative hypothesis.

Previous observations that mutations in *MED12* (X-linked; mostly hemizygous) and *MED13L* (heterozygous) were associated with generally similar phenotypes, including ID, hypotonia, and other congenital anomalies,^6^, ^7^, ^8^, ^48^ led to the coining of a so-called “mediatorpathy” phenotype.^49^ More recently, heterozygous mutations in *MED13* were also considered to fit this pattern.^9^ Interestingly, in the case of *MED12*, it was demonstrated experimentally that the substitutions p.Arg961Trp and p.Asn1007Ser (causing FG and Lujan syndromes, respectively), exhibit impaired recruitment of CDK8 onto GLI3-target gene promoters, leading to hyperactivated GLI3-dependent SHH signaling.^50^ Here, we find that the major clinical features (including moderate ID, hypotonia, ASD, ADHD, and CHD) associated with *CDK8* mutations again show substantial phenotypic overlap, lending further support to the general concept of a Module-related syndrome. By contrast, relatively fewer (to date, up to four replicated: *MED17*, *MED20*, *MED23*, and *MED25*) mutations in the other 26 non-Module components of the Mediator have been described; bi-allelic mutations in *MED17* or *MED20* (both members of the Mediator’s Head) produce progressive cerebral and/or cerebellar atrophy, whereas bi-allelic mutations in *MED23* or *MED25* cause syndromic or non-syndromic ID (summarized in Table S3).

In summary, therefore, our observations support the proposed clustering of developmental disorders related to perturbed function of the Module (“Mediator kinase modulopathy”). Given the pleiotropic actions of the Module, many mechanisms, including defects in key developmental signaling pathways, might lead to these pathologies. For example, the severe developmental defects identified in embryos with *Med12* hypomorphic mutations are produced by impairment of the canonical and Wnt/PCP signaling pathways;^51^ analogously, CDK8 has a pivotal role as a regulator of several additional signaling pathways, including WNT, TGFβ/BMP, STAT1, SHH, and NOTCH.^52^, ^53^ Thus, a disruption of the proper activity of CDK8 could modify any of these developmental signaling networks.

Although the idea of a Mediator kinase modulopathy as a pathogenic entity is attractive, several cautions are necessary. Genetic evidence from *Drosophila* formally demonstrates the non-equivalence of different Module components, on the basis of differences in the respective phenotypes when comparing mosaic knockouts for a *Cdk8*-*Ccnc* pair compared with a *Med12*-*Med13* pair.^54^ This situation is compounded for vertebrates, in which paralogous pairs exist for three of the four Module subunits. Indeed, the single described disruption (caused by a chromosomal inversion) of the *CDK8* paralog *CDK19* was associated with a different phenotype that comprised microcephaly, congenital bilateral falciform retinal folds, nystagmus, and learning disability;^55^ however, the functions of CDK8 and CDK19 are known to be divergent.^56^ Finally, it remains uncertain to what extent, and in which circumstances, Module activity might be kinase-independent because these functions would likely be unaffected by the mutations described here.^2^ Further work on the structure and function of the Mediator will doubtless shed light on these issues.

## Declaration of Interests

A.H., T.K., O.P., M.J.O.R., J.B., and P.A.C. are current or former employees of The Institute of Cancer Research (ICR), which has a commercial interest in the development of WNT pathway inhibitors, and they have received awards-to-inventor payments for the discovery and development of CDK8 and CDK19 inhibitors in partnership with Merck. M.J.O.R. is currently an employee of Merck, and J.B. is currently an employee of Azeria Therapeutics and NeoPhore. K.G.M. and A.T. are employees of GeneDx, a wholly owned subsidiary of OPKO Health.

## Web Resources

Clustal Omega, https://www.ebi.ac.uk/Tools/msa/clustalo/
DECIPHER, https://decipher.sanger.ac.uk/
ExAC Browser, http://exac.broadinstitute.org/
GeneMatcher, https://genematcher.org/
gnomAD Browser, https://gnomad.broadinstitute.org/
MutationTaster, http://www.mutationtaster.org/
OMIM, http://www.omim.org/
PolyPhen-2, http://genetics.bwh.harvard.edu/pph2/
PubMed, https://www.ncbi.nlm.nih.gov/pubmed
RCSB Protein Data Bank (PDB), https://www.rcsb.org/
Servier Medical Art, https://smart.servier.com/
SIFT, http://sift.jcvi.org/
UniProt, https://www.uniprot.org/

## Supplemental Data

Document S1. Supplemental Note, Supplemental Material and Methods, Supplemental References, Figure S1, and Tables S1, S4, and S5

Table S2. Detailed Clinical Information from Standardized Questionnaire on Subjects with *CDK8* Substitutions

Table S3. Mediator Components, Sequence Constraint, and Mendelian Diseases

Document S2. Article plus Supplemental Data
