# Supplementary material for: De Novo Missense Substitutions in the Gene Encoding CDK8, a Regulator of the Mediator Complex, Cause a Syndromic Developmental Disorder
Source: Am J Hum Genet. 2019 Mar 21;104(4):709–20. doi: 10.1016/j.ajhg.2019.02.006 (PMC6451695; doi:10.1016/j.ajhg.2019.02.006)
Supplement: Document S1. Supplemental Note, Supplemental Material and Methods, Supplemental References, Figure S1, and Tables S1, S4, and S5 [file mmc1.pdf]

The American Journal of Human Genetics, Volume *104*

Supplemental Data

De Novo Missense Substitutions in the Gene Encoding CDK8, a Regulator of the Mediator Complex, Cause a Syndromic Developmental Disorder

Eduardo Calpena, Alexia Hervieu, Teresa Kaserer, Sigrid M.A. Swagemakers, Jacqueline A.C. Goos, Olajumoke Popoola, Maria Jesus Ortiz-Ruiz, Tina Barbaro-Dieber, Lucy Bownass, Eva H. Brilstra, Elise Brimble, Nicola Foulds, Theresa A. Grebe, Aster V.E. Harder, Melissa M. Lees, Kristin G. Monaghan, Ruth A. Newbury-Ecob, Kai-Ren Ong, Deborah Osio, Francis Jeshira Reynoso Santos, Maura R.Z. Ruzhnikov, Aida Telegrafi, Ellen van Binsbergen, Marieke F. van Dooren, The Deciphering Developmental Disorders Study, Peter J. van der Spek, Julian Blagg, Stephen R.F. Twigg, Irene M.J. Mathijssen, Paul A. Clarke, and Andrew O.M. Wilkie

Supplemental Note: Case Reports

Subject 1

This girl was born after an uneventful pregnancy. Immediately after birth a ptosis of the right eye was noticed. During the first year of life feeding difficulties, a vertical gaze palsy, hypotonia and motor delay became apparent. Pediatric neurological examination also showed facial weakness and a congenital myasthenic syndrome was suspected. A pyridostigmine treatment trial resulted in a mild, temporary improvement.

Language development was also delayed, with nasal speech and a hoarse voice. She has a sensorineural hearing loss (right: 35 dB, left: 22 dB). Because of a mild intellectual disability (ID), visual impairment and attention deficit hyperactivity disorder (ADHD), she visits a special educational needs school. Behavioral problems include ADHD, tantrums and anxiety. Until the age of 6 yr she has had recurrent febrile episodes.

Array-CGH was normal. Clinical whole exome sequencing (WES; performed in-house as a parent-child trio) did not identify any known pathogenic variants; however the *de novo* c.79G>C (p.Val27Leu) substitution in *CDK8* was highlighted as a variant of unknown significance (VUS) and submitted to GeneMatcher.

An additional *de novo* missense variant was identified in the *CACNA1A* gene (gnomAD: missense Z = 6.19, o/e = 0.55; loss-of-function [LoF] pLI = 1.00, o/e = 0.05). The *CACNA1A* NM_023035.2:c.2570G>A (p.Arg857His) variant is not present in gnomAD. Mutations affecting the *CACNA1A* gene are responsible for the autosomal dominant epileptic encephalopathy, early infantile (MIM: 617106), episodic ataxia, type 2 (MIM: 108500), migraine, familial hemiplegic (MIM: 141500) or spinocerebellar ataxia 6 (MIM: 183086) disorders. This variant has not been identified before in any of these disease cohorts. Although the missense variant is located outside the intervals of the transmembrane domains (I-IV), in a region apparently more tolerant to missense variation,[1] we cataloged this as a VUS.

Additionally, a *de novo* mosaic variant (present in ~20% of cells) was identified in the *HDAC9* gene (gnomAD: missense Z = 2.27, o/e = 0.73; LoF pLI = 1.00, o/e = 0.13).

The *HDAC9* NM_178423.1:c.646C>T (p.Arg216*) variant is not present in gnomAD. Although it was suggested that *HDAC9* deletions could represent a risk factor for autism, ID and schizophrenia associated with incomplete penetrance/variable expressivity,[2] the involvement of *HDAC9* mutations in congenital disorders has not been defined.

Subject 2

This 3-year old male was born at 37+5/7 weeks by forceps-assisted vaginal delivery after an uncomplicated pregnancy. Apgar scores were 7 and 8 at 1 and 5 min, respectively. The neonatal course was significant for respiratory distress thought to be secondary to meconium aspiration, hyperbilirubinemia requiring phototherapy, and feeding difficulty. He did not pass his newborn hearing screen, which was initially thought to be related to antibiotics given as part of sepsis rule-out during his neonatal intensive care unit (NICU) course.

At 7 weeks of age, he was admitted for failure-to-thrive. He also had increased respiratory effort during feeds as well as oral motor difficulties, reflux, and inefficient suck. He was found to have laryngomalacia and a sleep study demonstrated severe obstructive sleep apnea requiring supplemental oxygen. A nasogastric tube was also placed for supplemental nutrition. During this hospitalization he was noted to have low tone and subtle dysmorphic features prompting further work-up for a neurologic and genetic disorder. Metabolic screening (plasma amino acids, urine organic acids, acylcarnitine profile, lactic acid and creatine kinase) was unrevealing, as were a single nucleotide polymorphism (SNP) chromosomal microarray and methylation studies for Prader-Willi and Angelman syndromes. Magnetic resonance imaging (MRI) of the brain at 5 mo showed nonspecific white matter abnormalities including a thin corpus callosum with bilateral posterior supratentorial and perihippocampal white matter volume loss, with possible hypomyelination.

He went on to demonstrate significant delays in his development in all domains. An evaluation at 12-13 mo showed age equivalencies of 3-4 mo for gross and fine motor skills, and 6-7 mo for social development. At the most recent assessment (aged 3 yr) he was able to roll and hold his head up, as well as sit propped up in a chair but not

unsupported. He could reach for toys and grasp with a full hand but had no pincer grasp. He says “mama” and “papa” but no other words, he responds to his name and tracks well. On general examination he is dysmorphic with brachycephaly and a tall and broad forehead, shallow orbits with the appearance of proptosis, low set ears with overfolded superior helices bilaterally and a tented mouth that is held open (low facial tone). His growth parameters are all within the normal range. Neurologic exam showed an alert toddler with no verbal output, good tracking and attention to sound. He was able to follow some simple commands from parents with prompting. He has marked global hypotonia with normal reflexes and no clonus, as well as frequent non-purposeful complex stereotypic movements of his distal extremities.

He is currently followed by multiple specialties, including ophthalmology for nystagmus, high myopia, and optic nerve cupping of both eyes; neurology; audiology for sensorineural hearing loss; and an aerodigestive team for feeding (he now has a G tube in place for nutrition) as well as respiratory concerns. He no longer requires oxygen and has had some improvement in swallowing, however aspiration continues to be a risk. He has had a normal echocardiogram.

As part of his diagnostic work-up, trio whole exome sequencing was performed by GeneDx, which identified a *de novo* missense variant of uncertain significance in the gene *CDK8* (c.85C>G, p.Arg29Gly).

Subject 3

This 9-year old boy was born as the second child, after an uneventful pregnancy of non-consanguineous parents, with a severe trigonocephaly, a perimembranous ventricular septal defect with a double orifice mitral valve, and pyloric hypertrophy for which surgery was required. Measurements of thyroid function were normal. No intracranial anomalies were seen on the preoperative CT scan and an ultrasound of the kidneys was normal. An uneventful fronto-supraorbital reconstruction was performed at 10 mo.

His development was delayed and he started walking at age 2.5 yr and talking at age 4 yr. At 8 yr an IQ of 55 was measured; his social behavior was like a 12- to 18-month old. He had trouble going to sleep for which melatonin was used. The diagnosis of

autism spectrum disorder (ASD) and of ADHD was made; the ADHD worsened in response to Ritalin and was thus stopped. On ophthalmological review he had intermittent exotropia and surso adductorius in both eyes with V-motility. Vision was 0.5 for both eyes but somewhat unreliable due to a lack of concentration.

Whole genome sequencing (WGS), performed on the parent-child trio as part of a research study of craniosynostosis, did not identify any known pathogenic variants. Three *de novo* substitutions were identified, of which the c.88G>A (p.Gly30Ser) mutation in *CDK8* was selected for further investigation.

Two additional *de novo* missense variants were identified in the *TRPM2* (gnomAD: missense Z = -0.21, o/e = 1.02; LoF pLI = 0.00, o/e = 0.89) and *COL3A1* (gnomAD: missense Z = 4.40, o/e = 0.59; LoF pLI = 1.00, o/e = 0.04) genes. The *TRPM2* NM_003307:c.2557G>A (p.Glu853Lys) variant has been observed in gnomAD (6/281772, 2.129e-5). Additionally, in gnomAD there are additional variants affecting the same amino acid (p.Glu853Gln, p.Glu853Asp). We considered the variant unlikely to have clinical significance. The *COL3A1* NM_000090, c.2621T>C (p.Phe874Ser) variant is not present in gnomAD, although there is a variant affecting the same amino acid (p.Phe874Cys). Mutations affecting the *COL3A1* gene are responsible for the autosomal dominant Ehlers-Danlos syndrome, vascular type (MIM: 130050). This variant has not been identified before in Ehlers-Danlos syndrome cohorts. Although this missense variant differs from the typical signature of causative variants identified in Ehlers-Danlos syndrome,[3] we cataloged this as a VUS.

Subject 4

This girl, currently aged 7.6 yr, was born to a 38 year old G2 P1 mother. The pregnancy was complicated by pneumonia, for which she took antibiotics. The prenatal ultrasound revealed a hypoplastic left heart and agenesis of the corpus callosum. She was born by caesarean section at 38 weeks' gestation, and was small for gestational age (birthweight 2.3 kg, length 47 cm). She was cyanotic and hypoxic at birth, and underwent cardiac surgery at 4 days of age via a Norwood procedure. She has subsequently undergone two additional cardiac surgeries, a bi-directional Glenn procedure aged 4 mo and a Fontan procedure aged 4 yr. She had dysphagia requiring a gastrostomy tube aged 7 mo, but now eats completely by mouth. She wears glasses

for myopia and has normal hearing. A brain magnetic resonance imaging (MRI) confirmed agenesis of the corpus callosum and mild ventriculomegaly.

She has a history of failure to thrive and remains small for her age, with her height at -2.53 SD, weight at -1.92 SD, and a normal occipito-frontal circumference (OFC). She is mildly dysmorphic, with bilateral epicanthal folds, prominent eyes, a small nasal tip with a short columella and a broad nasal base, broad thumbs and broad, laterally deviated great toes, hypoplastic distal phalanges of all toes, planovalgus feet, and mild hypotonia.

Her development has been globally delayed. She did not roll or sit until 18 mo, walked at 4 ½ yr, said her first word at 4 yr, and spoke in sentences at 6 yr. She is in special education classes at school and receives speech, physical, occupational, developmental and music therapies. She has ADHD, with a sweet personality, but is sometimes aggressive, and has a severe sleep disorder.

Clinical WES was performed on the parent-child trio by GeneDx and did not identify any known pathogenic variants; however the *de novo* c.185C>T (p.Ser62Leu) substitution in *CDK8* was reported as a VUS and submitted to GeneMatcher.

Subject 5

This individual is a 2-day old boy born after an uneventful pregnancy at 41+0 weeks' gestation via vaginal delivery after induced labor. He is the second child of unrelated parents. At birth he weighed 3120 grams. He was born with an ano-rectal malformation, an atrial septal defect, ventricular septal defect (VSD), bicuspid aortic valve, and a hypoplastic aortic arch. On examination no dysmorphic features were present except for low-set ears, which were slightly rotated backwards. He was lost to follow-up at the age of 6 mo, at which time he was unable to sit independently.

SNP-array showed a paternally inherited duplication at 7q11.21 (chr7:63,352,782-63,856,505; hg19)x3 considered likely to be a benign finding. Clinical WES was performed on the parent-child trio at the same institution as for Subject 1. Although this did not identify any known pathogenic variants, the *de novo* c.185C>T (p.Ser62Leu) substitution in CDK8 was highlighted as being of potential significance.

An additional *de novo* missense variant was identified in the *TRAF6* gene (gnomAD: missense Z = 2.85, o/e = 0.54; LoF pLI = 1.00, o/e = 0.05). The *TRAF6* NM_004620.3: c.1193C>T (p.Pro398Leu) variant is not present in gnomAD. The variant does not affect any of the three zinc finger domains, although is located within a region that is likely involved in protein interactions. The implication of mutations of *TRAF6* in congenital disorders is not well defined so far. A *de novo* LoF (frameshift mutation located in the last exon of the *TRAF6*) was described in one proband with hypohidrotic ectodermal dysplasia,[4] whereas a homozygous deletion affecting the 5′UTR of *TRAF6* was recently identified in a subject with an atypical form of osteopetrosis.[5] We cataloged the identified variant as a VUS.

Subject 6

This individual is a 10 mo female, born to white parents aged 28 yr (mother) and 39 yr (father), and was initially referred for clinical genetics evaluation at the age of 5 mo owing to agenesis of the corpus callosum, aortic coarctation and global developmental delay. She has one healthy older sister. The mother had two previous miscarriages. On examination she had a large head (occipito-frontal circumference [OFC] 47.5 cm, +2.21 SD), with hypertelorism, sparse eyebrows, long columella and downturned corners of the mouth, with axial hypotonia and hypertonia of the lower extremities. Initial workup included a normal karyotype and SNP array. She underwent aortic coarctation repair aged 5 mo with a good result. When she was last evaluated at the age of 10 mo she had made some developmental progress, but was significantly delayed with milestones equivalent to 5 mo (rolling over, sitting with support).

Clinical WES was performed on the parent-child trio by GeneDx and did not identify any known pathogenic variants; however the *de novo* c.185C>T (p.Ser62Leu) substitution in *CDK8* was highlighted as a VUS and submitted to GeneMatcher.

Subject 7

This male subject is the only child of his parents. At birth he was noted to have low set ears, redundant skin in the neck, lymphedema of the hands and feet, undescended testes, and echocardiography showed tetralogy of Fallot. An initial diagnosis of

Noonan Syndrome was proposed. He underwent cardiac surgery at 9 mo. Assessment at 7 mo demonstrated mild delay of motor development and he continued to have low muscle tone throughout childhood. By 8 yr he had developed complex partial seizures and migraines, both improved with valproic acid. He had mild-moderate ID and was in special educational classes.

Genetic testing including array-comparative genomic hybridization (array-CGH), *CHD7* and *KAT6B* did not reveal any pathogenic mutations.

Assessment at the age of 16 yr demonstrated persistence of low muscle tone, including low oral muscle tone with dribbling. He had an abnormal narrow palate with marked dental decay. He has had bilateral lateral column lengthening and serial plaster casting for *pes planus* and can walk for approximately 2 miles. He is registered as partially sighted due to a severe myopia. He attended school for children with special educational needs. At the age of 22 yr he was given a diagnosis of childhood autism.

He was enrolled as a singleton into the Deciphering Developmental Disorders (DDD) WES project, which did not identify any pathogenic variants of known clinical significance. The heterozygous c.185C>T (p.Ser62Leu) variant in *CDK8* was identified through DDD CAP#144 and subsequently confirmed on dideoxy-sequencing to be present in the subject and absent in his mother. The father was unavailable for analysis.

Subject 8

This child is the second of two boys born to unrelated parents. The older brother has epilepsy but no other medical problems. The father (who was not available for genetic testing) is well; the mother, who has moderate learning difficulties, tested negative for the *CDK8* c.185C>T variant identified in her son; there is no other family history of learning problems.

He was born after an uneventful pregnancy at 41 weeks' gestation with a birth weight of 4.3 kg. No neonatal problems were reported. He was seen by a pediatrician at 5 mo because of developmental delay, marked hypotonia and brachycephaly with marked flattening over the right occiput. At almost 2 yr, he had marked head lag when lifted

from supine and could roll over. He was dysmorphic with down-slanting palpebral fissures, slightly low set right ear and alternating divergent squint. Growth measurements were normal. Past investigations included normal karyotype, Fragile X and basic metabolic screening. He had a brain MRI which showed agenesis of the corpus callosum.

At the age of almost 4 yr he had generalized hypotonia (deep tendon reflexes in the upper and the lower limbs were not elicited; plantars down going) and positional kyphosis. He had limited communication skills (single words only). He was mainly transported in a buggy but was able to use a standing frame for about 45 minutes a day. Since early childhood he had an anemia of unknown origin, which improved with iron supplements.

At the age of 5.5 yr he was reviewed in the genetics clinic. He still had mild plagiocephaly. Hypotonia was improving; he had significant generalized joint laxity. Aged 7 yr he had a microarray which showed a maternally inherited duplication at Xq21.31 (chrX:86315153-86578362; hg18) containing no RefSeq genes within the minimum region and considered to be an innocent finding.

At the last genetic review aged 12.7 yr his problems were global developmental impairment, ASD and ADHD, dysmorphic features and plagiocephaly, absent corpus callosum, generalized hypotonia, gastro-esophageal reflux with food regurgitation, recurrent loose stool and intermittent rectal mucosal prolapse, strabismus (wears corrective glasses). His height and weight were between the 2nd and 9th centile. He is regularly seen by the child adolescent mental health service. He attends a SEN school and due to social issues he is a child in care.

He was enrolled as a singleton into the DDD project, which did not identify any pathogenic variants of known clinical significance. The heterozygous c.185C>T (p.Ser62Leu) variant in *CDK8* was identified through DDD CAP#144 and subsequently confirmed on dideoxy-sequencing to be present in the subject and absent in his mother.

Subject 9

She is the 5th child of healthy, unrelated parents and was born at term following a normal pregnancy. She first came to medical attention at a few days of age with paroxysmal events consisting of eye rolling and limb jerking. These events were captured during an electroencephalogram (EEG) recording and were not felt to be epileptic. She had failed her neonatal hearing screen and was diagnosed with sensorineural hearing loss; profound on the right and moderate on the left. MRI brain examination at the time was normal other than some hippocampal asymmetry.

Developmental delay was evident from 4 mo. A diagnosis of a seizure disorder was made by one year of age and Lamotrigine treatment started. A repeat MRI scan showed no progression of the previously observed changes but noted incomplete myelination. An additional diagnosis of a movement disorder consisting of tremor and dystonia was made by 4 yr. She continues with epileptic and non-epileptic paroxysmal events that are exacerbated by pyrexia. Investigations into her movement disorder have shown a low cerebrospinal fluid (CSF) homovanillic acid level, the significance of which is unclear. At 8 yr she has moderate delay in all developmental areas and a diagnosis of autism. She is well grown (OFC 25th centile), normally proportioned and has a facial appearance in keeping with that of her family. She exhibits abnormal posturing of all four limbs, an overt tremor of her upper limbs, but has normal power and deep tendon reflexes. She has mild peripheral joint hypermobility, but normal skin texture and elasticity.

Array-CGH was normal. She was enrolled with her parents into the DDD and 100,000 Genomes (WGS) projects, neither of which identified pathogenic variants of known clinical significance. The *de novo* c.291T>G (p.Phe97Leu) variant in *CDK8* was identified through DDD CAP#144.

Subject 10

He was born at term following an unremarkable pregnancy. He is the third child of healthy unrelated parents. He had mild hyperbilirubinemia as a baby that did not require any treatment. There were no developmental concerns prior to the age of 1 year.

He was first assessed by the clinical genetics team at 5 yr, by which time he had global developmental delay and acquired microcephaly (OFC centiles at 3 mo on the 50th, at 2 yr on the 25th and at 5 yr on the 2nd). He walked from around 2 yr, but remained unsteady and had a few single words of speech only with no ability to use Makaton or PECS cards. His receptive language abilities were a little better than this – assessed at the 24 mo level. His interactive and social skills had been assessed as at the 36 mo level. Other than his head circumference his general growth was in keeping with that of his family. He had generally been healthy, had a normal sleep pattern and there were no concerns regarding his hearing or vision.

On examination he was well-proportioned with an unsteady broad-based gait. He was very active and had a friendly personality. He had a normal skull shape, his eyes were large and he had an open mouthed expression with mild prognathism and wide-spaced teeth. His hands, feet and genitalia were normal. Skull radiographs did not show any evidence of craniosynostosis.

Array-CGH showed a 220 kb deletion on chromosome 21q22.3 (chr21:44779709-45001192, hg18) which was not felt to be of clinical significance. Methylation-specific analysis excluded common mechanisms of Angelman syndrome. DDD WES (performed as a singleton) identified three heterozygous variants in Developmental Disorders Genotype-to-Phenotype Database (DDG2P) genes (*AFF* c.1757G>A, p.Ser586Asn; *LRP5* c.2900C>T, p.Pro967Leu; and *SETBP1* c.3022C>A, p.Arg1008Ser), all of which were shown to have been inherited from one or other parent and not considered to be significant. The c.533G>A (p.Arg178Gln) variant in *CDK8* was identified through DDD CAP#144, and confirmed by dideoxy-sequencing to be present in the subject and absent in both parents. Correct sample relationships were confirmed by analysis of 13 microsatellite markers.

Subject 11

This girl is the first child born to healthy, unrelated Sri Lankan parents. There was no family history of note. The pregnancy was uncomplicated. Birth weight was 2.6 kg at 38 weeks' gestation. She was readmitted at 1 week of age because of hyperbilirubinemia, which needed treatment with phototherapy and an exchange transfusion. This was attributed at the time to breast milk jaundice. She was noted to

have a laryngeal stridor early in life; this resolved spontaneously after 1-2 yr. She was delayed in reaching her milestones, sitting at around 9-10 mo, and walking at around 18 mo. She learned to ride a bike with stabilizers at the age of 6 yr.

At the age of 3 yr she developed cyclical vomiting. This problem has persisted, and aged 12 yr she continues to be treated with flunarazine. She also takes salbutamol as required for treatment of asthma. She has had grommets inserted on two occasions for bilateral glue ear.

Academically she is making reasonable progress, with a particular strength in reading. She has an excellent memory. At school she is good at history, geography and English literature, but struggles in maths. She does have some specific learning difficulties; at the age of 12 yr she is unable to draw a straight line using a ruler, and has difficulties drawing a table or graph. These skills are felt to be around 5 yr behind her chronological age.

She is described as having a volatile personality. She can become easily disappointed if her requests are not met, and can be verbally aggressive at times; this tends to occur at home and be directed towards immediate family. At other times she is described as being sweet and charming. She can be overtalkative; non-verbal clues are often not understood. Anxiety can be an issue. Whilst she is not particularly hyperactive, and her attention span is reasonable when undertaking leisure activities such as reading, she is described as having some features compatible with ADHD. When she was younger her sleep was very restless, but she now sleeps well, although struggles to wake in the morning.

Socially, she has several long term friends. She loves small babies and wants to be a kindergarten teacher.

She was enrolled with her parents into the DDD (WES) project, which did not identify any pathogenic variants of known clinical significance. The *de novo* c.578T>G (p.Val193Gly) variant in *CDK8* was identified through DDD CAP#144.

Subject 12

This boy, a 3.5-year-old Armenian/Caucasian male, was referred for clinical genetics assessment secondary to history of hypotonia. His parents initially became concerned during the first year of life owing to delay in achieving his gross motor milestones. As an infant he was bottle-fed because of inability to latch onto the breast, but otherwise he had no feeding difficulties and weight gain was satisfactory.

He had been followed by the cardiology team secondary to a VSD, which has not required any surgical repair. His last echocardiogram, performed at the age of 6 mo showed one small 2 mm mid-muscular VSD and a second tiny anterior muscular VSD. He has passed his vision screening in his primary care physician's office, and his newborn hearing screen. A more recent hearing evaluation was non-contributory owing to lack of co-operation.

He was diagnosed with autism aged 3 yr by his school psychologist and is currently enrolled in a special needs academic program at the pre-kindergarten level. He receives physical therapy, speech therapy, and occupational therapy. His growth measurements are normal. He is mildly dysmorphic, with a broad mouth, long philtrum which is well-defined, broad nasal tip, slightly down-slanted palpebral fissures and prominent ears; in addition, persistent fetal finger pads bilaterally and slight internal rotation of the medial malleoli were noted. He wears ankle-foot orthoses, which help with ambulation. He demonstrates a positive Gower maneuver when moving from a seated to standing position. Serum creatine kinase was normal. Fragile X and SNP chromosome microarray testing was normal. Clinical WES was performed on the parent-child trio by GeneDx and did not identify any known pathogenic variants; however the *de novo* c.669A>G (p.Ile223Met) substitution in *CDK8* was highlighted as a VUS.

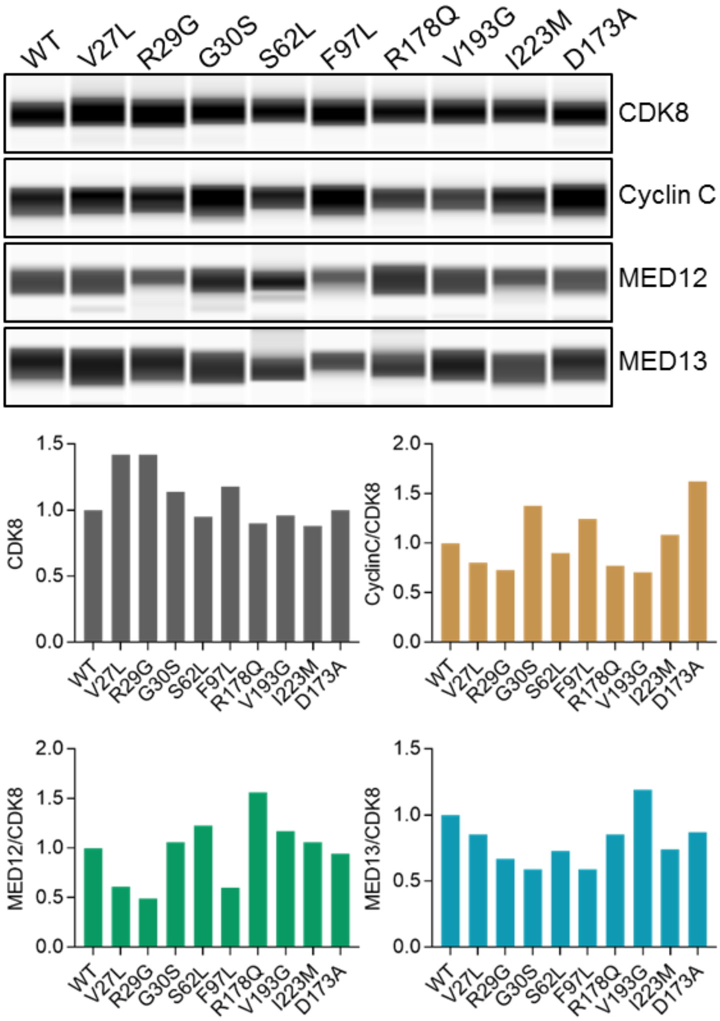

Figure S1. Detection of CDK8, cyclin C, MED12 and MED13 in the purified CDK8 Modules. Lysates from HEK293T cells transiently transfected with Myc-FLAG-tagged WT or mutant CDK8 constructs were used for immunoprecipitation using anti-FLAG antibody or mouse IgG isotype control antibody. The immunocomplexes were purified using magnetic beads with protein G. CDK8 was released by adding FLAG peptide and an aliquot of the eluted fraction was analyzed by automated capillary immunoassay system to detect CDK8, cyclin C, MED12 and MED13 (virtual blots at the top; relative quantifications from this experiment below).

	Subject 1	Subject 2	Subject 3	Subjects 4-8	Subject 9	Subject 10	Subjects 11	Subject 12
**Genomic coordinate**	chr13:26254720	chr13:26254726	chr13:26254729	chr13:26337623	chr13:26349158	chr13:26385229	chr13:26385274	chr13:26393389
**DNA variant**	c.79G>C	c.85C>G	c.88G>A	c.185C>T	c.291T>G	c.533G>A	c.578T>G	c.669A>G
**Protein change**	p.Val27Leu	p.Arg29Gly	p.Gly30Ser	p.Ser62Leu	p.Phe97Leu	p.Arg178Gln	p.Val193Gly	p.Ile223Met
**Inheritance**	*de novo*	*de novo*	*de novo*	*de novo*[a]	*de novo*	*de novo*	*de novo*	*de novo*
**MutationTaster**	disease causing	disease causing	disease causing	disease causing	disease causing	disease causing	disease causing	disease causing
**SIFT**	damaging	damaging	damaging	damaging	tolerated	damaging	damaging	damaging
**Polyphen-2**	possibly damaging	probably damaging	possibly damaging	probably damaging	benign	probably damaging	probably damaging	probably damaging

Genomic coordinates from GRCh38. Nomenclature is based on the NM_001260.2 transcript. [a]This variant was demonstrated *de novo* in subjects 4-6 and was negative in the maternal samples of individuals 7 and 8, for whom paternal samples were unavailable.

Table S1. Summary of *CDK8* mutations and analyses of pathogenicity

Primer	Sequence (5'-3')	Variants to screen
CDK8-gDNA-Exon1-F	CCTCAGAGGCTGTGACAATGG	c.79G>C (p.V27L), c.85C>G (p.R29G), c.88G>A (p.G30S)
CDK8-gDNA-Exon1-R	GCGTCGAGGAAGTCAGAGCC	
CDK8-gDNA-Exon2-F	ATCAAATAATGGGACTGGACTAGG	c.185C>T (p.S62L)
CDK8-gDNA-Exon2-R	ACAATACAGAGCCTTATATAGTTGG	
CDK8-gDNA-Exon3-F	TTTACCTGTGGGTTTGTATTGCC	c.291T>G (p.F97L)
CDK8-gDNA-Exon3-R	AATACTCTAGCCTTCGTCATGCC	
CDK8-gDNA-Exon6-F	GTAACAGTAATTTGAAGAGAATGG	c.533G>A (R178Q), c.578T>G (p.V193G)
CDK8-gDNA-Exon6-R	TTAATTAGTTCTACTCACCAATAGC	
CDK8-gDNA-Exon7-F[a]	cacgacgttgtaaaacgacGGGTGGAGGGCATGGGAATA	c.669A>G (p.I223M)
CDK8-gDNA-Exon7-R[a]	ggataacaatttcacacaggACCTCTCCAACGAAGACATCTAAGT	

[a]Primers for the exon 7 amplification include universal M13-tails (lower case).

Table S4. Primers used for validation of the genomic variants in *CDK8*

Primers for the directed mutagenesis	Forward (5'-3')	Reverse (5'-3')
CDK8-SDM-V27L	GGGCTGCAAAcTTGGCCGAGG	TCGTATTCAAACAGGTCCTC
CDK8-SDM-R29G	CAAAGTTGGCgGAGGCACTTATG	CAGCCCTCGTATTCAAAC
CDK8-SDM-G30S	AGTTGGCCGAaGCACTTATGG	TTGCAGCCCTCGTATTCAAAC
CDK8-SDM-S62L	ATCTCTATGTtGGCATGTAGAG	CCCAGTTCCTTCTATTTG
CDK8-SDM-F97L	GGCTTCTGTTgGACTATGCTG	ACACCTTCCTATCAGCATG
CDK8-SDM-R178Q	GGCTTTGCCCaATTATTTAATTC	CATGTCAGCAATTTTTACTC
CDK8-SDM-V193G	TTGGATCCAGgGGTTGTTACA	ATCTGCTAAAGGCTTCAAAG
CDK8-SDM-I223M	TAGGGTGTATgTTTGCAGAAC	TAGCCCAAATATCAATAGC
CDK8-SDM-D173A	AAAATTGCTGcCATGGGCTTTG	TACTCTTCCTCGCTCAGG
**Primers for sequencing**	**Sequence (5'-3')**	
CDK8-R1	TCGCTCAGGACCTTCACCC	
CDK8-F2	GAAGCCAGTTCAGTTACCTCG	
CDK8-F3	TGCAGCCTTATCAAGTATATGG	

Table S5. Mutagenesis and sequencing primers

Supplemental Material and Methods

Exome/genome sequencing and validation

Trio whole genome sequencing of Subject 3 was performed by Complete Genomics (Mountain View, CA, USA), as described by Drmanac et al.[6] Data were analyzed using cga tools version 1.6.0.43. A *de novo* disease model was tested, using the 'calldiff script' (Python script kindly provided by Complete Genomics) as described by Gilissen et al.[7] Prioritization of variants was based on the minimal somatic score and manual curation of the variants.

Trio exome sequencing of Subjects 1 and 5 (Utrecht cases) was performed as follows: exomes were enriched using the SureSelect XT Human All Exon V5 kit (Agilent) and sequenced in rapid run mode on the HiSeq2500 sequencing system (Illumina) at a mean target depth of 100x. The target was defined as all coding exons of UCSC and Ensembl +/- 20 bp intron flanks. At this depth ~95% of the target was covered at least 15x. Reads were aligned to hg19 using BWA (BWA-MEM v0.7.5a) and variants were called using the GATK haplotype caller (v2.7-2). Detected variants were annotated, filtered and prioritized using the Bench NGS Lab platform (Cartagenia, Leuven, Belgium). Analysis was based upon a tiered approach. The first tier analyzed known intellectual disability genes. The second tier filtered for *de novo* variants and the last tier filtered for recessive variants.

Trio exome sequencing of Subjects 2, 4, 6 and 12 (GeneDx cases) was performed as follows. Using genomic DNA from the proband and parents, the exonic regions and flanking splice junctions of the genome were captured using the Clinical Research Exome kit (Agilent Technologies, Santa Clara, CA) or the IDT xGen Exome Research Panel v1.0. Massively parallel sequencing was done on an Illumina system with 100 bp or greater paired-end reads. Reads were aligned to human genome build GRCh37/hg19, and analyzed for sequence variants using a custom-developed analysis tool. Additional details of the sequencing technology and variant interpretation protocol have been described previously.[8] The general assertion criteria for variant classification are publicly available on the GeneDx ClinVar submission page.

Exome sequencing of Subjects 7, 8 and 10 (as singletons), and 9 and 11 (as trios) was performed as part of the DDD research study.[9] Variant data were obtained as part of the approved Complementary Analysis Project #144 from Datafreeze 3, comprising 7,833 trios and 1,792 singletons with undiagnosed developmental disorders, primarily developmental delay/learning disability.

All the *CDK8* variants were validated by dideoxy-sequencing of the probands, and all available parental samples were analyzed by exome/genome and/or dideoxy-sequencing to assess inheritance of the *CDK8* mutations. In subject 10, in whom the *CDK8* variant was identified in a sample sequenced as a singleton, correct sample relationships of the trio were checked by demonstrating consistent inheritance of 13 microsatellite *loci* (*D1S2868*, *D3S1311*, *D4S403*, *D5S2027*, *D6S1610*, *D7S519*, *D9S158*, *D10S548*, *D11S898*, *D13S1265*, *D14S280*, *D16S415* and *D18S474*). The primers used for the PCR and dideoxy-sequencing validations of the genomic variants are listed in the Table S4.

Protein alignment

CDK8 protein sequences (FASTA format) from *H. sapiens* (human; P49336), *M. musculus* (mouse; Q8R3L8), *X. tropicalis* (western clawed frog; Q6P3N6), *D. rerio* (zebrafish; Q8JH47), *D. melanogaster* (fruit fly; Q9VT57), *A. aegypti* (mosquito; Q17IE8), *C. elegans* (nematode worm; P90866), and the human CDK19 protein sequence (Q9BWU1), the vertebrate paralogue of CDK8,[10] were downloaded from Uniprot and a multiple protein sequence alignment was conducted by using Clustal Omega to evaluate the conservation of the CDK8 residues mutated in the subjects of this study.

Structure preparation

A homology model of CDK8 and cyclin C was generated in MOE 2015.10[11] using the CDK8-cyclin C complex structure (PDB entry 4F7S)[12] as template and the Uniprot[13] sequence identifier P49336 (CDK8 isoform 1) and P24863 (cyclin C isoform 1) to fill in gaps in CDK8 and remove artificial N-terminal residues in cyclin C in the input structure for molecular dynamics simulation. The homology model was aligned to the

CDK9 structure (PDB entry 3BLQ)[14] and ATP and Mg^{2+} were copied into the CDK8 structure. The CDK8 complex (referring to the structure of CDK8 + ATP + Mg^{2+} + cyclin C) was prepared using the default settings of the protein preparation wizard in Maestro release 2017-2.[15] Mutations were introduced using MOE.

Simulation

ATP topologies and parameters were generated using CGenFF webserver and cgenff_charmm2gmx.py python script (MacKerell lab webserver). The protein, solvent and ions were parameterized using the July 2017 update of the CHARMM36 force field[16] in GROMACS version 2016.4.[17-23] The simulations were performed in a cubic box with a minimum distance to the box boundaries of 10 Å, solvated with TIP3P[24] waters and neutralized with chloride ions. After minimization, the system was equilibrated with 100 ps runs using both NVT and NPT ensemble where N = 138057 (CDK8 complex WT), 138059 (CDK8 complex p.Ser62Leu), 138055 (CDK8 complex p.Arg178Gln); T= 300 K; and P = 1 bar. Production simulations were run at least twice for 50 ns. If the variation between runs was large, a third run was included. Time steps of 2 fs were used and frames were saved every 10 ps. Temperature and pressure were maintained at 300 K and 1 bar and controlled by modified Berendsen thermostat[25] and the Parrinello-Rahman[26] barostat. The LINCS[27] and Particle-Mesh Ewald[28] algorithms were applied to define bond parameters and long-range electrostatics. A radius cut-off of 12 Å was applied to short-range van der Waals and electrostatic terms. The analysis was performed using the GROMACS analysis tools (rms, distance, hbond, covar, and anaeig) and figures were created using PyMOL.[29]

Expression constructs

The pCMV6-CDK8-Myc-FLAG (Origene, RC212592), containing the human cDNA open reading frame of human *CDK8* (CDK8 WT) with C-terminal Myc-FLAG tags, was used for the functional analysis. The mutations in *CDK8* were generated by mutagenesis PCR using the primers listed in the Table S5 and following the Q5-Mutagenesis kit protocol (New England Biolabs). The previously described catalytically inactive CDK8 kinase-dead (p.Asp173Ala) mutant was used as control.[30-

[32] All constructs were verified by dideoxy-sequencing (R1, F2 and F3 primers in Table S5).

Cell culture, transfection and immunoblotting

The generation and full characterization of the SW620 *CDK8*/*CDK19* double-knockout cells will be described in detail elsewhere.[33] SW620 *CDK8*/*CDK19* double knockout and HEK293T cells were cultured in DMEM supplemented with 10% fetal bovine serum (FBS), L-glutamine, and penicillin-streptomycin at 37°C under 5% CO_2. For the functional assays transfections were performed using Lipofectamine 2000 (Thermo Fisher Scientific) according to the manufacturer's instructions and cells were processed after 24 h.

For immunoblotting, cells were collected and lysed in Cellytic M lysis buffer (Sigma-Aldrich) containing protease (Complete, Sigma-Aldrich) and phosphatase (PhosStop, Sigma-Aldrich) inhibitors. The lysates were clarified by centrifugation at 15,000 *g* for 15 min at 4°C and protein concentration was determined with the BCA Protein Assay Kit (Pierce, Thermo Scientific). All lysates were resolved on SDS-PAGE (Mini-Protean TGX 4-15% gradient gels, Biorad) and analyzed by Western blotting (WB) with specific antibodies. Primary antibodies used were anti-FLAG (F1804, Sigma-Aldrich), anti-CDK8 (4106S, Cell Signaling), anti-phospho-STAT1-Ser727 (8826, Cell Signaling), anti-STAT1 (total) (9176, Cell Signaling), anti-cyclin C (A301-989A-M, Bethyl Cambridge Bioscience) and anti-Myc (2278T, Cell Signaling). GAPDH was detected as a loading control using the HRP-conjugated anti-GAPDH (3683, Cell Signaling). For anti-phospho antibodies, the blocking reagent was 5% BSA in Tris-buffered saline, 0.1% Tween 20 (T-TBS) instead of non-fat milk. For quantitative analysis, captured bands were analyzed with the Image J software (National Institutes of Health, Bethesda, MD).

Immunoprecipitation, elution and thermal stability assay

We previously showed that ligand binding to the Mediator kinases determined by biochemical assays with recombinant CDK8/CDK19 and cyclin C is not always recapitulated in intact cells, suggesting that the other components of the kinase

module can influence CDK8 ligand-binding and activity.[30] Therefore we determined whether the CDK8 kinase domain substitutions had caused structural changes and/or affected the ability to bind ATP using whole kinase Module complexes isolated from cells. HEK293T cells were transiently transfected, harvested after 24 h, washed with phosphate-buffered saline (PBS) and lysed in 220 µl of immunoprecipitation lysis buffer (50 mM HEPES [pH 7.3], 150 mM NaCl, 1 mM $MgCl_2$, 1% Triton X100 [TX100]) containing protease (Complete, Sigma-Aldrich) and phosphatase (PhosStop, Sigma-Aldrich) inhibitors. The lysates were centrifuged at 15,000 *g* for 15 min at 4°C and the supernatant was transferred to a new tube. An aliquot of 20 µl was saved from the supernatant to analyze (by WB) the total cell lysate fraction (TCL). The remaining lysates (200 µl) were processed for immunoprecipitation. All the incubations and washes were done in a rotator at 4°C. Lysates were incubated with 1 µg of anti-FLAG or the mAb IgG1 isotype control (5415S, Cell Signaling) antibody for 4 h. Then, 50 µl of Dynabeads Protein G (Invitrogen) were added and incubated for 2 h. Beads containing the immunocomplexes were washed three times with washing buffer (20 mM HEPES [pH 7.3], 150 mM NaCl, 1 mM $MgCl_2$, 0.2% TX100, supplemented with protease and phosphatase inhibitors). The immunoprecipitates were released from the magnetic beads by adding 200 µl of washing buffer containing 200 µg/mL of the FLAG peptide (F3290, Sigma-Aldrich) and saved as eluted fraction. An aliquot of the eluted fraction was evaluated by WB to demonstrate the presence of the immunoprecipitated CDK8 protein and its specific interactor cyclin C. The remaining volume of the eluted fractions was used for the thermal stability assay. Each elution was diluted with kinase buffer (Cell Signaling Technology) and with or without ATP at 2 mM (Thermo Fisher Scientific). Each sample (with or without ATP) was divided into 12 equal aliquots and heated individually at different temperatures determined between 50 to 82°C for 3 min, using a gradient thermal cycler, and followed by 3 min cooling at 21°C. The samples were then centrifuged for 20 min at 4°C at 18,000 *g* to separate the soluble proteins from the protein precipitated/degraded. The supernatant was collected and the protein content was analyzed by capillary immunoassay following the manufacturer's protocol. The following antibodies were used when the capillary immunoassay system was employed: anti-CDK8 (4106S, Cell Signaling), anti-cyclin C (A301-989A, Bethyl Cambridge Bioscience), anti-MED12 (SC-5372, Santa-Cruz) and anti-MED13 (Abcam, Ab76923).

Statistical analysis

Graphs and statistical analyses were conducted with Prism 5 (GraphPad). The quantification of immunoblot analysis was performed from five independent experiments. P-values were calculated using one-way ANOVA with Dunnett's *post hoc* analysis. Values shown represent means ± standard error of the mean (SEM). In all figures, *$p \leq 0.05$, **$p \leq 0.01$, ***$p \leq 0.001$.

Web Resources

CGenFF webserver, https://cgenff.umaryland.edu/

GeneDx ClinVar submission page, http://www.ncbi.nlm.nih.gov/clinvar/submitters/26957/

MacKerell lab webserver, http://mackerell.umaryland.edu/

Supplemental References

1. Luo, X., Rosenfeld, J.A., Yamamoto, S., Harel, T., Zuo, Z., Hall, M., Wierenga, K.J., Pastore, M.T., Bartholomew, D., Delgado, M.R., et al. (2017). Clinically severe *CACNA1A* alleles affect synaptic function and neurodegeneration differentially. Plos Genet. *13*, e1006905.
2. Pinto, D., Delaby, E., Merico, D., Barbosa, M., Merikangas, A., Klei, L., Thiruvahindrapuram, B., Xu, X., Ziman, R., Wang, Z.Z., et al. (2014). Convergence of genes and cellular pathways dysregulated in autism spectrum disorders. Am. J. Hum. Genet. *94*, 677-694.
3. Frank, M., Albuisson, J., Ranque, B., Golmard, L., Mazzella, J.M., Bal-Theoleyre, L., Fauret, A.L., Mirault, T., Denarie, N., Mousseaux, E., et al. (2015). The type of variants at the *COL3A1* gene associates with the phenotype and severity of vascular Ehlers-Danlos syndrome. Eur. J. Hum. Genet. *23*, 1657-1664.
4. Wisniewski, S.A., and Trzeciak, W.H. (2012). A rare heterozygous *TRAF6* variant is associated with hypohidrotic ectodermal dysplasia. Brit. J. Dermatol. *166*, 1353-1356.
5. Hubshman, M.W., Basel-Vanagaite, L., Krauss, A., Konen, O., Levy, Y., Garty, B.Z., Smirin-Yosef, P., Maya, I., Lagovsky, I., Taub, E., et al. (2017). Homozygous deletion of *RAG1*, *RAG2* and 5' region *TRAF6* causes severe immune suppression and atypical osteopetrosis. Clin. Genet. *91*, 902-907.
6. Drmanac, R., Sparks, A.B., Callow, M.J., Halpern, A.L., Burns, N.L., Kermani, B.G., Carnevali, P., Nazarenko, I., Nilsen, G.B., Yeung, G., et al. (2010). Human genome sequencing using unchained base reads on self-assembling DNA nanoarrays. Science *327*, 78-81.
7. Gilissen, C., Hehir-Kwa, J.Y., Thung, D.T., van de Vorst, M., van Bon, B.W., Willemsen, M.H., Kwint, M., Janssen, I.M., Hoischen, A., Schenck, A., et al. (2014). Genome sequencing identifies major causes of severe intellectual disability. Nature *511*, 344-347.
8. Retterer, K., Juusola, J., Cho, M.T., Vitazka, P., Millan, F., Gibellini, F., Vertino-Bell, A., Smaoui, N., Neidich, J., Monaghan, K.G., et al. (2016). Clinical application of whole-exome sequencing across clinical indications. Genet. Med. *18*, 696-704.
9. Mcrae, J.F., Clayton, S., Fitzgerald, T.W., Kaplanis, J., Prigmore, E., Rajan, D., Sifrim, A., Aitken, S., Akawi, N., Alvi, M., et al. (2017). Prevalence and architecture of *de novo* mutations in developmental disorders. Nature *542*, 433-438.
10. Galbraith, M.D., Donner, A.J., and Espinosa, J.M. (2010). CDK8: a positive regulator of transcription. Transcription *1*, 4-12.
11. Molecular Operating Environment (MOE), 2015.10; Chemical Computing Group ULC, 1010 Sherbooke St. West, Suite #910, Montreal, QC, Canada, H3A 2R7, 2018.
12. Schneider, E.V., Bottcher, J., Huber, R., Maskos, K., and Neumann, L. (2013). Structure-kinetic relationship study of CDK8/CycC specific compounds. Proc. Natl. Acad. Sci. USA *110*, 8081-8086.

13. Bateman, A., Martin, M.J., O'Donovan, C., Magrane, M., Alpi, E., Antunes, R., Bely, B., Bingley, M., Bonilla, C., Britto, R., et al. (2017). UniProt: the universal protein knowledgebase. Nucleic Acids Res. *45*, D158-D169.
14. Baumli, S., Lolli, G., Lowe, E.D., Troiani, S., Rusconi, L., Bullock, A.N., Debreczeni, J.E., Knapp, S., and Johnson, L.N. (2008). The structure of P-TEFb (CDK9/cyclin T1), its complex with flavopiridol and regulation by phosphorylation. EMBO J. *27*, 1907-1918.
15. Schrödinger Release 2017-2: Maestro, Schrödinger, LLC, New York, NY, 2018.
16. Huang, J., Rauscher, S., Nawrocki, G., Ran, T., Feig, M., de Groot, B.L., Grubmuller, H., and MacKerell, A.D. (2017). CHARMM36m: an improved force field for folded and intrinsically disordered proteins. Nat. Methods *14*, 71-73.
17. Berendsen, H.J.C., Vanderspoel, D., and Vandrunen, R. (1995). GROMACS - a message-passing parallel molecular-dynamics implementation. Comput. Phys. Commun. *91*, 43-56.
18. Lindahl, E., Hess, B., and van der Spoel, D. (2001). GROMACS 3.0: a package for molecular simulation and trajectory analysis. J. Mol. Model. *7*, 306-317.
19. Van der Spoel, D., Lindahl, E., Hess, B., Groenhof, G., Mark, A.E., and Berendsen, H.J.C. (2005). GROMACS: fast, flexible, and free. J. Comput. Chem. *26*, 1701-1718.
20. Hess, B., Kutzner, C., van der Spoel, D., and Lindahl, E. (2008). GROMACS 4: algorithms for highly efficient, load-balanced, and scalable molecular simulation. J. Chem. Theory Comput. *4*, 435-447.
21. Pronk, S., Pall, S., Schulz, R., Larsson, P., Bjelkmar, P., Apostolov, R., Shirts, M.R., Smith, J.C., Kasson, P.M., van der Spoel, D., et al. (2013). GROMACS 4.5: a high-throughput and highly parallel open source molecular simulation toolkit. Bioinformatics *29*, 845-854.
22. Pall, S., Abraham, M.J., Kutzner, C., Hess, B., and Lindahl, E. (2015). Tackling exascale software challenges in molecular dynamics simulations with GROMACS. Lect. Notes Comput. Sci. *8759*, 3-27.
23. Abraham, M.J., Murtola, T., Schulz, R., Páll, S., Smith, J.C., Hess, B., and Lindahl, E. (2015). GROMACS: high performance molecular simulations through multi-level parallelism from laptops to supercomputers. SoftwareX *1-2*, 19-25.
24. Jorgensen, W.L., Chandrasekhar, J., Madura, J.D., Impey, R.W., and Klein, M.L. (1983). Comparison of simple potential functions for simulating liquid water. J. Chem. Phys. *79*, 926-935.
25. Berendsen, H.J.C., Postma, J.P.M., Vangunsteren, W.F., Dinola, A., and Haak, J.R. (1984). Molecular-Dynamics with coupling to an external bath. J. Chem. Phys. *81*, 3684-3690.
26. Parrinello, M., and Rahman, A. (1981). Polymorphic transitions in single-crystals - a new molecular-dynamics method. J. Appl. Phys. *52*, 7182-7190.
27. Hess, B., Bekker, H., Berendsen, H.J.C., and Fraaije, J.G.E.M. (1997). LINCS: a linear constraint solver for molecular simulations. J. Comput. Chem. *18*, 1463-1472.
28. Darden, T., York, D., and Pedersen, L. (1993). Particle mesh Ewald - An N Log(N) method for Ewald sums in large systems. J. Chem. Phys. *98*, 10089-10092.
29. PyMOL: the PyMOL molecular graphics system, Version 1.7.2.3 Schrödinger, LLC, New York, NY, 2018.
30. Dale, T., Clarke, P.A., Esdar, C., Waalboer, D., Adeniji-Popoola, O., Ortiz-Ruiz, M.J., Mallinger, A., Samant, R.S., Czodrowski, P., Musil, D., et al. (2015). A

selective chemical probe for exploring the role of CDK8 and CDK19 in human disease. Nat. Chem. Biol. *11*, 973-980.

31. Firestein, R., Bass, A.J., Kim, S.Y., Dunn, I.F., Silver, S.J., Guney, I., Freed, E., Ligon, A.H., Vena, N., Ogino, S., et al. (2008). *CDK8* is a colorectal cancer oncogene that regulates beta-catenin activity. Nature *455*, 547-551.
32. Gold, M.O., and Rice, A.P. (1998). Targeting of CDK8 to a promoter-proximal RNA element demonstrates catalysis-dependent activation of gene expression. Nucleic Acids Res. *26*, 3784-3788.
33. Ruiz, M.J.O., Popoola, O., Mallinger, A., Gowan, S., Court, W., Box, G., Valenti, M., Brandon, A.D.H., Te-Poele, R., Workman, P., et al. (2016). Elucidation of the different roles of CDK8 and CDK19 in colorectal cancer (CRC) using CRISPR gene editing technology. Cancer Res. *76(14 Suppl)*, Abstract nr 4355.
34. Guna, A., Butcher, N.J., and Bassett, A.S. (2015). Comparative mapping of the 22q11.2 deletion region and the potential of simple model organisms. J. Neurodev. Disord. *7*, 18.
35. Graham, J.M., Jr., and Schwartz, C.E. (2013). *MED12* related disorders. Am. J. Med. Genet. *161A*, 2734-2740.
36. Asadollahi, R., Zweier, M., Gogoll, L., Schiffmann, R., Sticht, H., Steindl, K., and Rauch, A. (2017). Genotype-phenotype evaluation of *MED13L* defects in the light of a novel truncating and a recurrent missense mutation. Eur. J. Med. Genet. *60*, 451-464.
37. Mukhopadhyay, A., Kramer, J.M., Merkx, G., Lugtenberg, D., Smeets, D.F., Oortveld, M.A.W., Blokland, E.A.W., Agrawal, J., Schenck, A., van Bokhoven, H., et al. (2010). *CDK19* is disrupted in a female patient with bilateral congenital retinal folds, microcephaly and mild mental retardation. Hum. Genet. *128*, 281-291.
38. Snijders Blok, L., Hiatt, S.M., Bowling, K.M., Prokop, J.W., Engel, K.L., Cochran, J.N., Bebin, E.M., Bijlsma, E.K., Ruivenkamp, C.A.L., Terhal, P., et al. (2018). *De novo* mutations in *MED13*, a component of the Mediator complex, are associated with a novel neurodevelopmental disorder. Hum. Genet. *137*, 375-388.
39. Vodopiutz, J., Schmook, M.T., Konstantopoulou, V., Plecko, B., Greber-Platzer, S., Creus, M., Seidl, R., and Janecke, A.R. (2015). *MED20* mutation associated with infantile basal ganglia degeneration and brain atrophy. Eur. J. Pediatr. *174*, 113-118.
40. Hashimoto, S., Boissel, S., Zarhrate, M., Rio, M., Munnich, A., Egly, J.M., and Colleaux, L. (2011). *MED23* mutation links intellectual disability to dysregulation of immediate early gene expression. Science *333*, 1161-1163.
41. Trehan, A., Brady, J.M., Maduro, V., Bone, W.P., Huang, Y., Golas, G.A., Kane, M.S., Lee, P.R., Thurm, A., Gropman, A.L., et al. (2015). *MED23*-associated intellectual disability in a non-consanguineous family. Am. J. Med. Genet. *167A*, 1374-1380.
42. Lionel, A.C., Monfared, N., Scherer, S.W., Marshall, C.R., and Mercimek-Mahmutoglu, S. (2016). *MED23*-associated refractory epilepsy successfully treated with the ketogenic diet. Am. J. Med. Genet. *170A*, 2421-2425.
43. Figueiredo, T., Melo, U.S., Pessoa, A.L.S., Nobrega, P.R., Kitajima, J.P., Correa, I., Zatz, M., Kok, F., and Santos, S. (2015). Homozygous missense mutation in *MED25* segregates with syndromic intellectual disability in a large consanguineous family. J. Med. Genet. *52*, 123-127.

44. Basel-Vanagaite, L., Smirin-Yosef, P., Essakow, J.L., Tzur, S., Lagovsky, I., Maya, I., Pasmanik-Chor, M., Yeheskel, A., Konen, O., Orenstein, N., et al. (2015). Homozygous *MED25* mutation implicated in eye-intellectual disability syndrome. Hum. Genet. *134*, 577-587.
45. Leal, A., Huehne, K., Bauer, F., Sticht, H., Berger, P., Suter, U., Morera, B., Del Valle, G., Lupski, J.R., Ekici, A., et al. (2009). Identification of the variant Ala335Val of MED25 as responsible for CMT2B2: molecular data, functional studies of the SH3 recognition motif and correlation between wild-type MED25 and PMP22 RNA levels in CMT1A animal models. Neurogenetics *10*, 275-287.
46. Leal, A., Bogantes-Ledezma, S., Ekici, A.B., Uebe, S., Thiel, C.T., Sticht, H., Berghoff, M., Berghoff, C., Morera, B., Meisterernst, M., et al. (2018). The polynucleotide kinase 3'-phosphatase gene (PNKP) is involved in Charcot-Marie-Tooth disease (CMT2B2) previously related to MED25. Neurogenetics *19*, 215–225.
47. Kaufmann, R., Straussberg, R., Mandel, H., Fattal-Valevski, A., Ben-Zeev, B., Naamati, A., Shaag, A., Zenvirt, S., Konen, O., Mimouni-Bloch, A., et al. (2010). Infantile cerebral and cerebellar atrophy is associated with a mutation in the *MED17* subunit of the transcription preinitiation Mediator complex. Am. J. Hum. Genet. *87*, 667-670.
48. Hirabayashi, S., Saitsu, H., and Matsumoto, N. (2016). Distinct but milder phenotypes with choreiform movements in siblings with compound heterozygous mutations in the transcription preinitiation Mediator complex subunit 17 (MED17). Brain Dev. *38*, 118-123.
49. Jeronimo, C., and Robert, F. (2017). The Mediator complex: at the nexus of DNA polymerase II transcription. Trends Cell Biol. *27*, 765-783.
