# Supplementary material for: De Novo Missense Substitutions in the Gene Encoding CDK8, a Regulator of the Mediator Complex, Cause a Syndromic Developmental Disorder
Source: Am J Hum Genet. 2019 Mar 21;104(4):709–20. doi: 10.1016/j.ajhg.2019.02.006 (PMC6451695; doi:10.1016/j.ajhg.2019.02.006)
Supplement: Document S2. Article plus Supplemental Data [file mmc4.pdf]

De Novo Missense Substitutions in the Gene Encoding CDK8, a Regulator of the Mediator Complex, Cause a Syndromic Developmental Disorder

Eduardo Calpena,[1] Alexia Hervieu,[2,18] Teresa Kaserer,[2,18] Sigrid M.A. Swagemakers,[3,18] Jacqueline A.C. Goos,[4,18] Olajumoke Popoola,[2] Maria Jesus Ortiz-Ruiz,[2] Tina Barbaro-Dieber,[5] Lucy Bownass,[6] Eva H. Brilstra,[7] Elise Brimble,[8] Nicola Foulds,[9] Theresa A. Grebe,[10] Aster V.E. Harder,[7] Melissa M. Lees,[11] Kristin G. Monaghan,[12] Ruth A. Newbury-Ecob,[6] Kai-Ren Ong,[13] Deborah Osio,[13] Francis Jeshira Reynoso Santos,[14,15] Maura R.Z. Ruzhnikov,[8] Aida Telegrafi,[12] Ellen van Binsbergen,[7] Marieke F. van Dooren,[16] The Deciphering Developmental Disorders Study,[17] Peter J. van der Spek,[3] Julian Blagg,[2] Stephen R.F. Twigg,[1] Irene M.J. Mathijssen,[4] Paul A. Clarke,[2,*] and Andrew O.M. Wilkie[1,*]

The Mediator is an evolutionarily conserved, multi-subunit complex that regulates multiple steps of transcription. Mediator activity is regulated by the reversible association of a four-subunit module comprising CDK8 or CDK19 kinases, together with cyclin C, MED12 or MED12L, and MED13 or MED13L. Mutations in *MED12*, *MED13,* and *MED13L* were previously identified in syndromic developmental disorders with overlapping phenotypes. Here, we report *CDK8* mutations (located at 13q12.13) that cause a phenotypically related disorder. Using whole-exome or whole-genome sequencing, and by international collaboration, we identified eight different heterozygous missense *CDK8* substitutions, including 10 shown to have arisen *de novo*, in 12 unrelated subjects; a recurrent mutation, c.185C>T (p.Ser62Leu), was present in five individuals. All predicted substitutions localize to the ATP-binding pocket of the kinase domain. Affected individuals have overlapping phenotypes characterized by hypotonia, mild to moderate intellectual disability, behavioral disorders, and variable facial dysmorphism. Congenital heart disease occurred in six subjects; additional features present in multiple individuals included agenesis of the corpus callosum, ano-rectal malformations, seizures, and hearing or visual impairments. To evaluate the functional impact of the mutations, we measured phosphorylation at STAT1-Ser727, a known CDK8 substrate, in a *CDK8* and *CDK19* CRISPR double-knockout cell line transfected with wild-type (WT) or mutant *CDK8* constructs. These experiments demonstrated a reduction in STAT1 phosphorylation by all mutants, in most cases to a similar extent as in a kinase-dead control. We conclude that missense mutations in *CDK8* cause a developmental disorder that has phenotypic similarity to syndromes associated with mutations in other subunits of the Mediator kinase module, indicating probable overlap in pathogenic mechanisms.

The Mediator complex is a large, multi-subunit assembly encoded by 26 genes in humans, and it regulates gene expression in all eukaryotes. The core function of the Mediator is to communicate developmental and physiological signals from DNA-bound transcription factors to RNA polymerase II (the enzyme responsible for the transcription of all protein-coding and most non-coding genes) by contacting the pre-initiation complex.[1,2] Regulation of Mediator activity is, in part, achieved by the reversible association of a four-subunit kinase module (hereafter referred to as the "Module") comprising cyclin C (encoded by *CCNC*) and either cyclin-dependent kinase 8 (CDK8), MED12, and MED13 or their respective paralogs CDK19, MED12L, and MED13L (Figure 1). Many targets of phosphorylation by CDK8 have been identified.[4] These include not only the core Mediator-RNA-polymerase-II complex (Module components [CDK8, cyclin C, MED12, MED13, and MED13L], other Mediator subunits [MED14 and MED26], and the C-terminal domain of RNA polymerase II) but also many other transcriptional regulators (for example, tissue-specific and general transcription factors, pause or release factors, chromatin-remodeling factors, and histone H3; reviewed by Jeronimo and Robert).[2] Mirroring this, genetic analyses of the Module have uncovered

[1]Clinical Genetics Group, MRC Weatherall Institute of Molecular Medicine, University of Oxford, Oxford OX3 9DS, UK; [2]Cancer Research UK Cancer Therapeutics Unit, the Institute of Cancer Research, London SM2 5NG, UK; [3]Department of Pathology and Department of Bioinformatics, Erasmus University Medical Center, University Medical Center Rotterdam, PO Box 2040, 3000 CA, Rotterdam, the Netherlands; [4]Department of Plastic and Reconstructive Surgery, Erasmus MC, University Medical Center Rotterdam, PO Box 2040, 3000 CA, Rotterdam, the Netherlands; [5]Genetics Division, Cook Children's Medical Center, Fort Worth, TX 76102, USA; [6]Department of Clinical Genetics, University Hospitals Bristol NHS Foundation Trust, St. Michael's Hospital, Bristol BS2 8EG, UK; [7]Department of Genetics, University Medical Center Utrecht, Utrecht University, 3508 AB Utrecht, the Netherlands; [8]Department of Neurology and Neurological Sciences, Stanford University School of Medicine, Stanford, CA 94305, USA; [9]Wessex Clinical Genetics Services, University Hospital Southampton, Southampton SO16 5YA, UK; [10]Department of Child Health, University of Arizona College of Medicine, Division of Genetics and Metabolism, Phoenix Children's Hospital, Phoenix, AZ 85016, USA; [11]North Thames Regional Genetics Service, Great Ormond Street Hospital for Children NHS Foundation Trust, London WC1N 3EH, UK; [12]GeneDx, Gaithersburg, MD 20877, USA; [13]Department of Clinical Genetics, Birmingham Women's and Children's NHS Foundation Trust, Birmingham B15 2TG, UK; [14]Genetics Division, Joe DiMaggio Children's Hospital, Hollywood, FL 33021, USA; [15]Charles E. Schmidt College of Medicine, Florida Atlantic University, Hollywood, FL 33021, USA; [16]Department of Clinical Genetics, Erasmus MC University Medical Center Rotterdam, PO Box 2040, 3000 CA, Rotterdam, the Netherlands; [17]Deciphering Developmental Disorders Study, Wellcome Sanger Institute, Cambridge CB10 1SA, UK
[18]These authors contributed equally to this work
*Correspondence: paul.clarke@icr.ac.uk (P.A.C.), andrew.wilkie@imm.ox.ac.uk (A.O.M.W.)
https://doi.org/10.1016/j.ajhg.2019.02.006.
© 2019 The Author(s). This is an open access article under the CC BY license (http://creativecommons.org/licenses/by/4.0/).

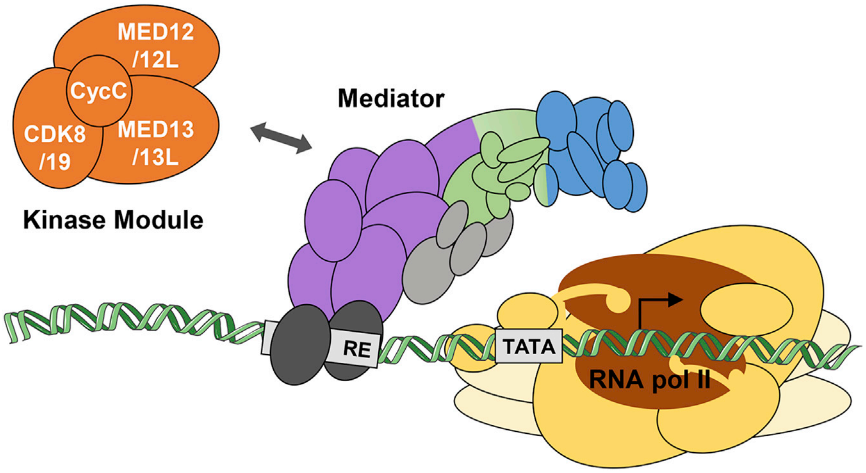

Figure 1. Simplified Illustration of the Mediator Complex and the RNA Pol II Machinery at the Promoter of a Hypothetical Gene

The CDK8 or CDK19 kinase Module (orange) reversibly binds the Mediator complex to regulate its activity. The Mediator complex (head in blue, middle in green, and tail in purple; additional subunits in light gray) bridges between gene-specific activators (dark gray) that are bound to regulatory elements (RE) and general transcription machinery that comprises the RNA pol II (brown) and general transcription factors (yellow). The DNA molecule cartoon was obtained from the Servier Medical Art; the representation was adapted from Larivière et al.[3]

many tissue- and species-specific functions, ranging from environmental responses in yeast to organogenesis and development in the nematode worm, fruit fly, zebrafish, and mouse.[5]

In humans, mutations in the genes (*MED12*, *MED13*, and *MED13L*) encoding subunits of the Module have been implicated in developmental disorders. Mutations in the X-linked *MED12* (MIM: 300188) cause at least three different syndromes: Opitz-Kaveggia syndrome (also known as FG syndrome; MIM: 305450), Lujan-Fryns syndrome (MIM: 309520), and Ohdo syndrome (MIM: 300895). These syndromes have partly overlapping clinical features, including multiple congenital defects, facial dysmorphic features, hypotonia, behavioral problems, and intellectual disability (ID).[6] Mutations in *MED13L* (MIM: 608771) are associated with a syndromic form of ID (MIM: 616789) characterized by facial dysmorphism, ID, speech impairment, motor developmental delay with muscular hypotonia, cardiac anomalies, and behavioral difficulties.[7,8] Recently, mutations in *MED13* (MIM: 603808) have been reported as leading to a neurodevelopmental disorder characterized by ID and/or developmental delay, including speech delay; additional features that were present in two or more affected individuals included autism spectrum disorder (ASD), attention deficit hyperactivity disorder (ADHD), optic nerve abnormalities, Duane anomaly, hypotonia, mild congenital heart abnormalities, and dysmorphic features.[9] Here, using whole-exome or whole-genome sequencing and by international collaboration, we report *de novo* mutations in *CDK8*, which has not been previously associated with a congenital disorder, in 12 unrelated individuals with overlapping phenotypes.

The study was initiated as part of a whole-genome sequencing (WGS)-based investigation of craniosynostosis in the Netherlands after approval by the board of the medical ethical committee Rotterdam (MEC-2012-140). Written informed consent was obtained from all participants or their legal guardians. Parent-child trio-based WGS of a proband with metopic synostosis and ID (subject 3 in Table 1) identified a *de novo* c.88G>A transition in *CDK8*; this change leads to the prediction of a heterozygous p.Gly30Ser substitution. The position of this substitution, at an almost invariant residue within the highly conserved glycine-rich loop of the kinase domain,[9] and in which pathogenic mutations were previously identified in other protein kinases,[22,57] led us to seek evidence for additional pathogenic variants in *CDK8* through GeneMatcher exchange[10] and from the Deciphering Developmental Disorders (DDD) research study.[11] The DDD study has UK research ethics committee (REC) approval (10/H0305/83, granted by the Cambridge South REC, and GEN/284/12 granted by the Republic of Ireland REC). Details of the methodology we used to identify each mutation (whole-exome sequencing [WES] in every instance except the original index subject) are provided in the Supplemental Material and Methods. By these means, we identified a further 11 unrelated individuals harboring eight different nucleotide substitutions within *CDK8*; one substitution (c.185C>T encoding p.Ser62Leu) was present in five subjects (Figure 2A and Table 1). All *CDK8* variants were constitutionally absent from the ExAC and gnomAD databases of common variation (accessed November 2018).[12]

CDK8 is a gene that comprises 13 exons; it is located at chromosomal region 13q12.13 and encodes a major isoform of 464 amino acids. All seven unique substitutions, and three of five instances of p.Ser62Leu, arose *de novo* from unaffected parents who had correct paternity confirmed either by comparison of trio-WES or WGS data or by microsatellite analysis; paternal samples were unavailable in the remaining two cases (the recurrence of the c.185C>T transition is likely to be explained by its position in a hypermutable CpG dinucleotide). The eight substituted amino acids are all located within the kinase domain, which extends between amino acids 21–335 (Figure 2B), and they are invariant in a wide range of vertebrate CDK8 orthologs, as well as in the human paralog CDK19; in six cases, this invariance extends to the invertebrates *Drosophila, Aedes*, and *Caenorhabditis* (Figure 2C). MutationTaster predicted all eight substitutions to be disease-causing, and SIFT and PolyPhen-2 predicted all to

Table 1. Clinical Features of Subjects with *CDK8* Mutations

Subject #	1	2	3	4	5	6	7	8	9	10	11	12
Gender	f	m	m	f	m	f	m	m	f	m	f	m
Age (years)	9	2.9	8	7.6	0.1	0.8	16.7	12.7	6.8	5.4	12	3.5
Mutation	c.79G>C	c.85C>G	c.88G>A	c.185C>T	c.185C>T	c.185C>T	c.185C>T	c.185C>T	c.291T>G	c.533G>A	c.578T>G	c.669A>G
Substitution	p.Val27Leu	p.Arg29Gly	p.Gly30Ser	p.Ser62Leu	p.Ser62Leu	p.Ser62Leu	p.Ser62Leu	p.Ser62Leu	p.Phe97Leu	p.Arg178Gln	p.Val193Gly	p.Ile223Met
De novo	yes	yes	yes	yes	yes	yes	NA	NA	yes	yes	yes	yes
Facial dysmorphism	+	+	+	+	+	+	+	+	–	+	+	+
Hypotonia, motor delay, and/or walking difficulty	+	+ (axial)	+	+	NA	+ (axial)	+ (bilateral *pes planus* surgery)	+	+	+	+	+
Brain MRI or CT	normal	thin corpus callosum	normal	ACC	NA	ACC	NA	ACC	non-specific	NA	normal	NA
Ophthalmic: ptosis	+	–	–	–	–	–	–	–	–	–	+	–
Strabismus	+	+	+	–	–	–	–	+	–	–	–	–
Myopia	+	+	–	+	–	–	+ (severe)	+	–	–	+	–
Impaired vision	+	–	–	–	–	–	+	–	–	–	–	–
Sensorineural hearing loss	moderate	moderate	–	–	NA	–	–	–	severe (unilateral)	NA	(glue ear)	–
Intellectual disability	mild (SEN school)	moderate to severe	moderate to severe (SEN school)	moderate	NA	NA; moderate motor delay	mild to moderate (SEN school)	moderate (SEN school)	moderate	moderate	mild (normal school)	NA; moderate motor delay
Behavioral disorder	ADHD	–	ASD, ADHD, sleep disorder	ADHD, sleep disorder	NA	NA	ASD	ASD, ADHD	ASD	happy disposition	attention seeking, volatile	ASD
Epilepsy	–	–	–	–	–	–	complex partial	–	general and focal	–	–	–
Feeding difficulties	infancy only	gastrostomy-fed	congenital pyloric stenosis	previous gastrostomy	NA	–	reflux	reflux, regurgitation	reflux, episodic vomiting	–	episodic vomiting	–

(*Continued on next page*)

Table 1. *Continued*

Subject #	1	2	3	4	5	6	7	8	9	10	11	12
Congenital heart disease (S indicates age at surgery)	–	–	perimembraneous VSD, double orifice mitral valve	hypoplastic left heart (S: 4 days, 3 months, 4 years)	atrial septal defect, VSD, bicuspid aortic valve, hypoplastic aortic arch (S: unknown)	coarctation of the aorta, subaortic stenosis, mitral stenosis (S: 5 months)	tetralogy of Fallot (S: 9 months)	–	–	–	–	VSD, PFO
Other			metopic synostosis		anterior anus, recto-perianal fistula		undescended testes	rectal mucosal prolapse				

Abbreviations and symbols are as follows: + = present; – = absent; NA = information not available; f = female; m = male; ACC = agenesis of the corpus callosum; ADHD = attention deficit-hyperactivity disorder; ASD = autism spectrum disorder; PFO = patent foramen ovale; SEN = special educational needs; and VSD = ventricular septal defect.

be damaging with the exception of the c.291T>G (p.Phe97Leu) variant (Table S1). *CDK8* is intolerant to missense variants (z = 3.81, observed/expected ratio = 0.34; gnomAD database)[12] and exhibits high constraint throughout the protein.[13] On the basis of the DOMINO algorithm, *CDK8* was predicted to be among the top candidate genes for which mutations are likely to manifest with a dominant pattern of inheritance.[14] The clustering of multiple *de novo* substitutions localized to a single, highly conserved domain of the protein provides a strong pathogenicity signature, as has been described for several other genes including the cyclin-dependent kinase (CDK)-encoding *CDK13*.[11,15,16]

We assessed the phenotypes of the 12 subjects, seven males and five females aged 0.1–16.7 years at the last clinical assessment, with CDK8 substitutions by using a standardized spreadsheet-based questionnaire (summarized in Table 1; full details can be found in Table S2, and case reports can be found in the Supplemental Note). Most affected children were born after unremarkable pregnancies, at or near term, with a birth weight within the normal range, and they did not require neonatal resuscitation, although around half required hospitalization after birth because of jaundice, seizures, laryngomalacia, or cardiac problems. More consistent difficulties emerged in early infancy: hypotonia (noted in 11/12 subjects) was frequently evident, and it later manifested as motor delay and sometimes persistent problems in walking. Mild to moderate developmental delay was universal, and older children had ID which ranged from mild (two cases) to moderate–severe (two cases); most were in the moderate range (five cases). Most children attended schools for special educational needs, but one had mainstream education. Behavioral symptoms were prominent: seven of ten of the older individuals had formal diagnoses of autism spectrum disorder (ASD) and/or attention deficit hyperactivity disorder (ADHD). The head circumference was normal except in one subject, who had mild macrocephaly (+2.21 standard deviations [SD]); magnetic resonance imaging (MRI) of the brain showed agenesis or thinning of the corpus callosum in four subjects, including the macrocephalic individual. Two individuals had seizures, but brain imaging was either normal or was not available. Three individuals had moderate-severe sensorineural hearing loss; ophthalmological abnormalities were frequent, including myopia (n = 6), eyelid ptosis (n = 2), and/or strabismus (n = 4), and these were associated with marked visual impairment in two children. Congenital heart defects (CHDs) were present in six of the twelve subjects; the defects were classified[17] as left ventricular obstruction (n = 3), conotruncal defects (n = 1), and other (n = 2). Present congenital gastrointestinal problems were ano-rectal abnormalities (n = 2) and pyloric stenosis (n = 1); two additional infants required feeding via gastrostomy tube. In later childhood, gastroesophageal reflux or episodic vomiting were significant management issues in four individuals. Facial dysmorphism was identified in 11 subjects; this was not

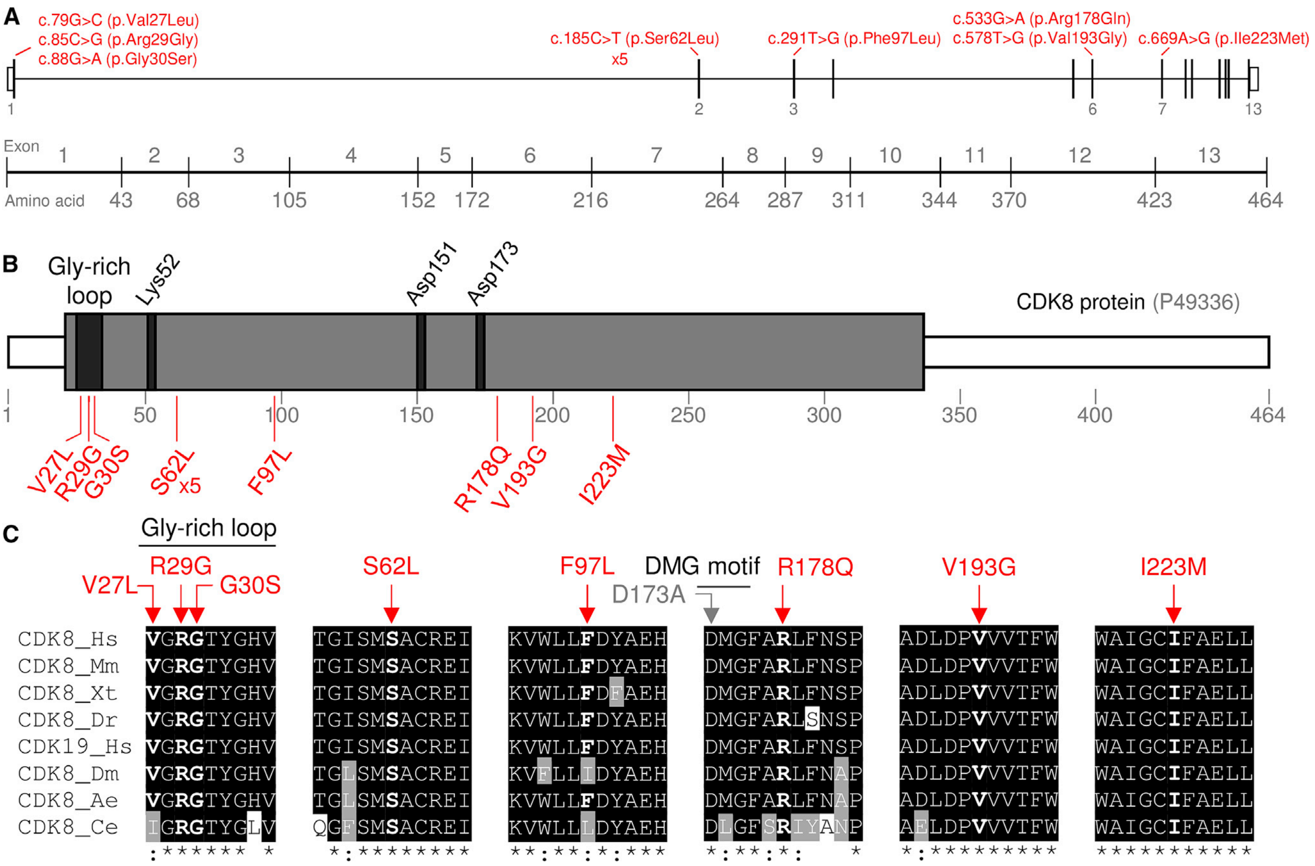

Figure 2. Mutations in *CDK8* and Conservation of Substituted Residues

(A) Localization of mutations (in red) in a schematic representation of human *CDK8* (exon numbering on GenBank: NM_001260.2).
(B) A cartoon of the human CDK8 (based on UniProt: P49336) showing the location of the protein kinase domain (21–335; gray box) and the eight different CDK8 substitutions identified in this work (below, in red). Note that the p.Ser62Leu substitution was identified in five unrelated subjects. Relevant functional elements are depicted in dark boxes. Note the Gly-rich loop (27–35); Lys52, a catalytic residue that interacts with the triphosphate of ATP in the active site; Asp151 within the conserved His-Arg-Asp (HRD) motif (at the catalytic loop), which is directly involved in catalysis; and Asp173, which is included in the Asp-Met-Gly (DMG) motif (at the activation segment) required for chelation of the magnesium ion involved in the catalysis. Substitution of Asp173 is widely used as a catalytically inactive kinase-dead form of CDK8 (p.Asp173Ala).
(C) A multiple-protein sequence alignment of CDK8 and CDK19 at the positions of the identified substitutions in CDK8. Abbreviations are as follows: Hs = human, Mm = mouse, Xt = western clawed frog, Dr = zebrafish, Dm = fruit fly, Ae = yellow fever mosquito, and Ce = nematode worm. The positions of the CDK8 *de novo* missense variants are indicated with a red arrow at the top of the sequences. Black- or gray-shaded amino acids indicate identical or similar residues compared to the human CDK8 sequence, respectively. Below the alignments, an asterisk (*) indicates positions that have a single, fully conserved residue, whereas a colon (:) indicates conservation between groups of similar properties. Additional relevant features present in these sequences (Gly-rich loop, DMG motif, and the Asp173 residue, mutated in the kinase-dead mutant) are also depicted.

characteristic, although arched eyebrows, epicanthic folds, prominent eyes, a prominent nasal tip or long columella, a long philtrum, wide or open-mouth posture, prognathism, and low set and prominent ears were highlighted in two or more subjects (Figure 3 and Table S2). The subjects' stature was within the normal range except in one individual, who had mild short stature (−2.53 SD). Apart from the metopic synostosis present in the index individual, no other individuals had craniosynostosis.

We assessed the probable effects of the eight individual amino acid substitutions on the CDK8 kinase domain by examining available protein structures (Figure 4). Three of the substitutions (c.79G>C [p.Val27Leu], c.85C>G [p.Arg29Gly], and p.Gly30Ser) are clustered in the Gly-rich loop (amino acids 27–35), a highly conserved protein-kinase motif with a critical role in ATP binding and in the phosphoryl-transfer reaction.[21–23] Substitutions within this motif affect kinase activity and, in other kinases, have been described as pathogenic mutations responsible for various congenital disorders.[15,22,24,25] Within the three-dimensional structure, all CDK8 substitutions either affect amino acids surrounding the ATP-binding pocket (Figure 4B) or are in contact with key functional amino acids; for example, the p.Ile223 residue is not directly part of the ATP-binding site but is within interacting distance of p.Lys153, which directly binds to the ATP phosphate in the input structural model. The recurrently mutated p.Ser62 residue is located in the αC-helix,[26] the conformation and interactions of which are important for CDK8 activity.[27] Two substitutions, c.533G>A

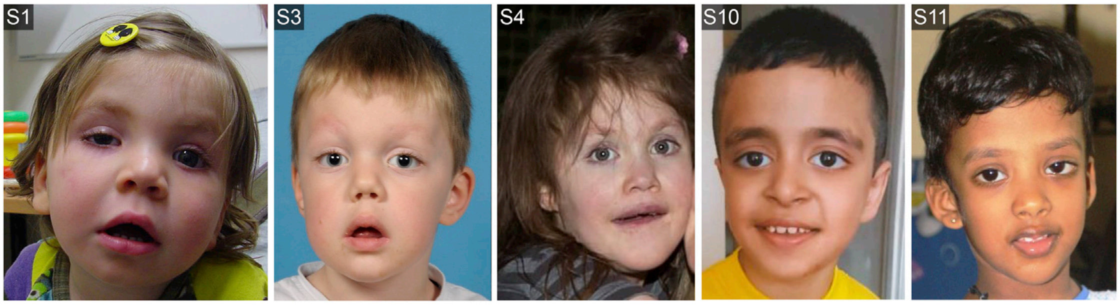

Figure 3. Clinical Pictures of Subjects with *CDK8* Mutations
Subjects (left to right): 1 (c.79G>C [p.Val27Leu]) aged 16 months, 3 (c.88G>A [p.Gly30Ser]) aged 4 years (postoperative after surgery to correct metopic synostosis), 4 (c.185C>T [p.Ser62Leu]) aged 9 years, 10 (c.533G>A [p.Arg178Gln]) aged 9 years, and 11 (c.578T>G [p.Val193Gly]) aged 2.5 years.

(p.Arg178Gln) and c.578T>G (p.Val193Gly), affect amino acids located in the activation segment,[26] which interacts with other key amino acids (Figure 4B). One of the substitutions (p.Phe97Leu) affects the "gatekeeper" p.Phe97 of CDK8. Mutations in the gatekeeper residue of kinases have emerged as a key mechanism by which cancer cells develop resistance to treatment,[28] highlighting the importance of the gatekeeper residue controlling the back cavity of the ATP site.[29]

To gain further insights into the effects of the mutations, we conducted molecular-dynamics simulations for wild-type (WT) and two selected CDK8 substitutions, p.Ser62Leu, and p.Arg178Gln, in complex with cyclin C and in the presence of ATP. The overall CDK8 protein structure and organization was predicted to be unchanged in the mutant models compared to in the WT. However, we found that in addition to impacting the ATP-binding site, the mutants were also predicted to induce pronounced structural changes in the substrate-binding site (Figure 4C). In particular, the substrate-binding site of the WT CDK8 protein can adopt more open conformations than its mutated counterparts, potentially allowing substrates to bind more closely in the vicinity of ATP in the WT protein.

To determine whether the CDK8 kinase domain substitutions had caused structural changes and/or affected the ability to bind ATP, we purified CDK8 kinase Modules that were isolated from cells by immunoprecipitation after the expression of WT or mutant-tagged-CDK8 (Figures 5A and S1), and we recorded protein-melting curves in the absence or presence of ATP (Figure 5B). In the absence of ATP, the thermal stability of mutant proteins ranged from indistinguishable to increased in comparison to the WT protein. Moreover, the stability of most mutant proteins was increased by the addition of ATP, in some cases to a greater extent than occurred in the WT protein. Together these results suggest that none of the substitutions cause gross mis-folding of the protein, and most or all are still able to bind ATP.

Finally, we investigated how the CDK8 substitutions affected the kinase activity by measuring phosphorylation of one of its well-validated targets: p.Ser727, of the human transcription factor STAT1.[30–34] We used a CRISPR-Cas9-engineered human cell line (SW620 colorectal carcinoma cells) that lacks both CDK8 and CDK19 (M.J. Ortiz-Ruiz et al., Cancer Res., abstract). These cells exhibited baseline STAT1-Ser727 (pSTAT1) phosphorylation that was substantially increased when WT CDK8 was transiently re-expressed (Figure 6). We performed site-directed mutagenesis to introduce the eight observed variants into *CDK8* cDNA (Supplemental Material and Methods), which we transfected into the *CDK8* and *CDK19* CRISPR double-knockout cells. We observed that the cellular levels of pSTAT1 were reduced to a statistically significant extent, compared to WT, in all mutant-transfected cells (Figure 6). For most mutants, the reduction in kinase activity was similar to that in the catalytically inactive CDK8 kinase-dead (p.Asp173Ala) mutant we used as a control, but in two mutants (p.Phe97-Leu and [c.669A>G] p.Ile223Met), an intermediate level of phosphorylation (~63% and ~51% in comparison to the WT, respectively), was observed.

Collectively, our genetic and functional assessments provide strong evidence for a previously unrecognized (to our knowledge) clinical disorder caused by heterozygous missense substitutions located in the kinase domain of CDK8. All eight distinct mutations were shown to have arisen *de novo* from unaffected parents (Table 1) and exhibited a similar combination of molecular characteristics. All clustered within the kinase domain (Figure 2) and were concentrated around the ATP binding pocket (Figure 4); none was associated with gross protein instability, and all retained the ability to bind ATP (Figure 5). Finally, all exhibited attenuated kinase activity in a previously validated assay of STAT1-phosphorylation, although the magnitude of the effect was diminished for two substitutions (Figure 6), suggesting partial retention of activity in those cases. Of note, the CDK8 mutant proteins maintained their ability to bind cyclin C, similar to the ability of the kinase-dead mutant (Figures 5A and S1, and as previously described).[35] The CDK8 kinase-dead mutant was previously shown to form a stable Module, although the inactive kinase was unable to phosphorylate its targets.[35,36]

Concordant with these molecular observations was a relatively consistent phenotypic presentation (Figure 3,

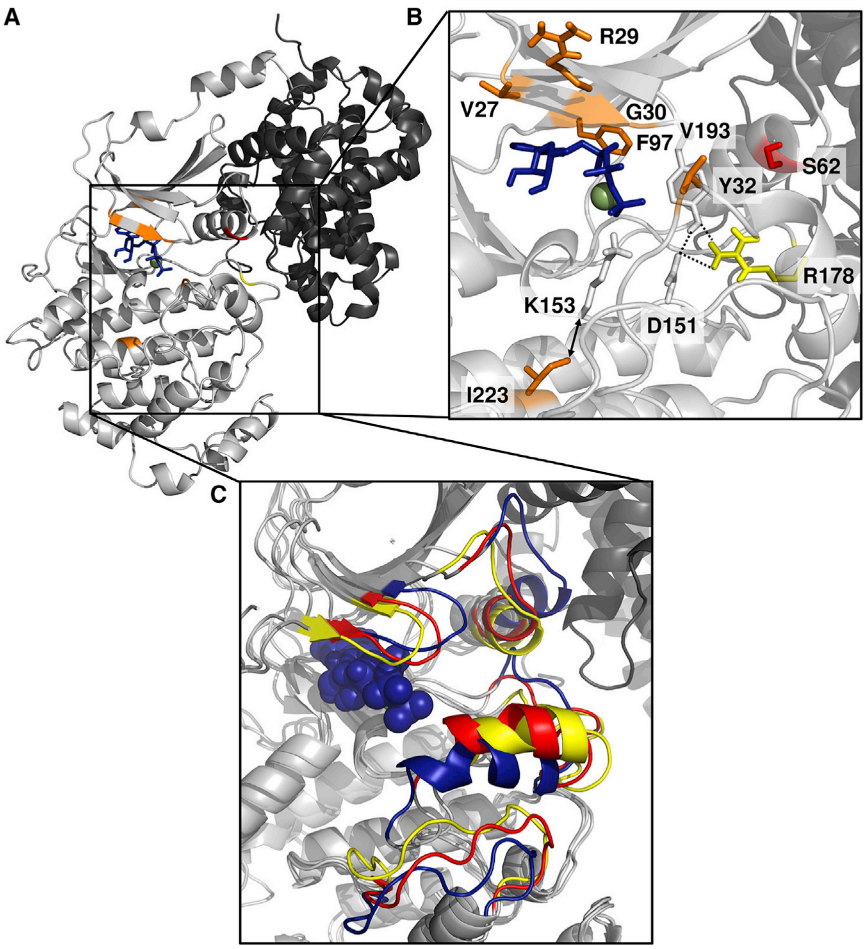

Figure 4. Location and Effect of CDK8 Substitutions on the Protein Structure
(A) Overall structure of CDK8 (light gray) in complex with cyclin C (dark gray). ATP (blue) was modeled into the CDK8 DMG-in crystal structure (PDB: 4F7S)[18] using aligned CDK9 (PDB 3BLQ).[19] Regions not previously resolved in the crystal structure have been modeled using Molecular Operating Environment (MOE).[20]
(B) A magnified view of the catalytic region of the CDK8 kinase domain. The Mg^{2+} is shown as a green sphere. Amino acids at which mutations have been identified are colored in orange (except Ser62 in red and Arg178 in yellow), and relevant, potential interaction partners in the input structure are shown as sticks. Ile223 is in close proximity (double-headed arrow) to the Lys153 involved in interaction with ATP-phosphate. Arg178 forms a hydrogen bond with Tyr32 (Gly-rich loop) and interacts ionically with the catalytic Asp151.
(C) A magnified overlay of the wild-type (WT) and mutant molecular-dynamics simulations of CDK8 (light gray) in complex with cyclin C (dark gray) in the presence of ATP (blue spheres). The areas displaying the most pronounced structural differences within the substrate binding site are colored (blue: WT, red: p.Ser62Leu, and yellow: p.Arg178Gln).

Table 1, Supplemental Case Reports, and Table S2). Hypotonia was usually evident in infancy and, in children old enough for assessment, learning disability was universal and most commonly classified as moderate; most but not all children were attending schools for special educational needs. Associated behavioral problems, which were frequent and added to the management issues for these children, included formal diagnoses of ASD and ADHD, sleep disorders, and episodic vomiting. Dysmorphic facial features, including arched eyebrows, a bulbous or upturned nose, and hypotonic facies, were frequent; however, these do not constitute a consistent or easily recognizable phenotype. Six of 12 subjects had congenital heart malformations, including at least three requiring corrective surgery. Additional medically significant features are documented in Table 1. The high frequency of diagnosis of ASD, ADHD, and CHD in these children is of note because *CDK8* mutations have not previously been highlighted in genetic screens of those disorders;[17,37] this might be because the target size for mutations of *CDK8* (missense substitutions surrounding the ATP-binding pocket) is relatively small. Although the mutation in our index subject, who had metopic synostosis, was originally identified in a genetic study of craniosynostosis, none of the 11 other subjects identified had this phenotype. It is unclear whether the co-occurrence of the mutation and craniosynostosis is causally linked (potentially through the adverse effect of the mutation on brain development)[38] or is coincidental.

In more than two decades since *CDK8* was first identified in independent studies of the yeast *Saccharomyces cerevisiae* (from screens for the stabilization of meiotic mRNAs [*Ume5*][39] and suppression of an RNA polymerase II C-terminal domain mutation [*Srb10*]),[40] a large literature has accumulated about its functions.[5,41] As described above and illustrated in Figure 1, *CDK8* encodes a kinase component of the Module, a four-subunit complex that binds to the Mediator and regulates its activity. Many fundamental uncertainties remain about its function, for example how the association and dissociation of the Module with the Mediator is regulated, how the Module regulates gene expression both positively and negatively, and the extent to which kinase activity *per se* is required for these functions.[2] Previous studies of human *CDK8* have mostly focused on its potential as a therapeutic target in cancer, on the basis of the observation that amplifications of *CDK8* are frequent in colon cancer.[42] Although effective CDK8 inhibitors have been developed, to date they have exhibited unacceptable toxicity.[31,32] This toxicity might reflect the essential function of CDK8 for normal development, as shown by the pre-implantation lethality of homozygous-null *Cdk8* mutations in the mouse; importantly

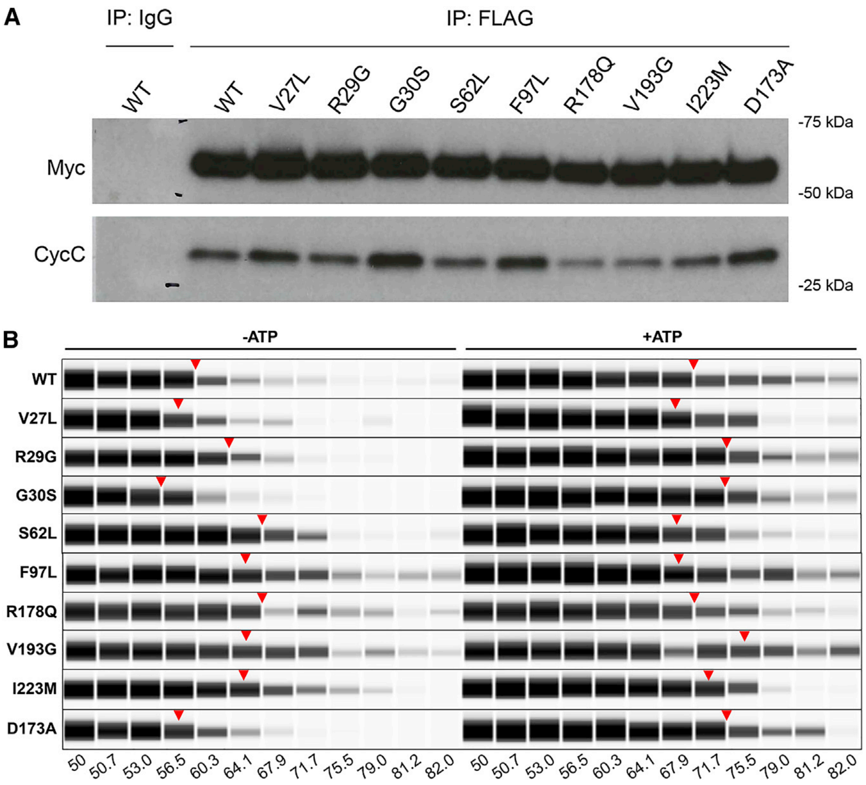

Figure 5. Immunoprecipitation of CDK8 and Thermal Stability Assay

(A) Lysates from HEK293T cells transiently transfected with Myc-FLAG-tagged wild-type (WT) or mutant *CDK8* constructs were used for immunoprecipitation with anti-FLAG antibody or mouse IgG isotype control antibody. The immunocomplexes were purified with protein G magnetic beads. CDK8 was released by adding FLAG peptide, and an aliquot of the eluted fraction was saved for immunoblot analysis with anti-Myc or anti-cyclin C (CycC) antibodies.

(B) A thermal stability assay of the eluted fractions incubated in the absence or presence of ATP and heated individually at different temperatures (gradient between 50–82°C). Virtual blot views of results for WT and the mutants are shown. For each CDK8 construct, the red arrowhead indicates 50% reduction of the CDK8 signal compared to the signal at the lowest temperature.

in relation to the discussion below, heterozygous animals were reported to be normal and fertile.[43]

If one focuses on the developmental role of CDK8, our observations show that although a single WT *CDK8* allele is sufficient for survival, heterozygosity for a mutant allele that encodes a missense substitution of the kinase domain causes pleiotropic developmental abnormalities. Broadly speaking, heterozygous mutations might be associated with abnormal phenotypes through one of three mechanisms: haploinsufficiency, gain of function, and dominant-negative activity.[44] When haploinsufficiency occurs, many mutations are expected to be either truncating or partial or whole-gene deletions. Deletions involving *CDK8* are very rare, and those recorded involve many other genes, so they are essentially uninformative. Although our gene-matching methods would not detect deletions, they were unbiased with regard to the nature of the intragenic mutation. It was previously reported that 381 of 706 *de novo* mutations in haploinsufficient genes were truncating,[11] so the finding that 11/11 (excluding the index subject) intragenic nucleotide substitutions encode missense changes diverges significantly from a haploinsufficiency pattern ($p = 0.0004$). Moreover, human *CDK8* is only moderately constrained to loss of function (probability that the gene is intolerant to a loss of function (LoF) mutation (pLI) = 0.38, gnomAD database); it has six (out of ~250,000) bona fide truncating alleles listed in gnomAD, suggesting that such alleles exist at low frequency in the normal population. Overall, haploinsufficiency appears unlikely to be the underlying mechanism that explains the *CDK8*-mutation-associated phenotype.

Instead, our experimental observations indicate that the mutations retain ATP binding yet lose or diminish the ability to phosphorylate a well-established substrate, STAT1. This might result from reduced ATP catalysis and phosphoryl-transfer and/or altered substrate binding resulting from reduced accessibility of the substrate-binding site predicted by the dynamic modeling (Figure 4). Notably, CDK8 (together with its paralog CDK19) are the only components of the Module with catalytic activity, so we predict that up to 50% of CDK8 modules could be present in a kinase-inactive, non-productive state. The consequence of this would then depend on the extent to which supply of the catalytically-active Module was rate-limiting to key cellular processes in particular developmental contexts. The notion that the supply of active Modules might frequently be limiting is supported by the observation that all six other genes encoding components of the human Module (*CCNC*, *CDK19*, *MED12*, *MED12L*, *MED13*, and *MED13L*) exhibit very strong constraint against loss-of-function variation (pLI = 1, observed/expected scores 0–0.11), indicating that reduction of any Module component by 50% impairs survival (by contrast, only three of 26 genes encoding other components of the Mediator exhibit pLI = 1; Table S3). Together, these observations currently support a dominant-negative mechanism of pathogenesis for CDK8 substitutions. Similar patterns of localized missense substitutions have been highlighted as a signature of dominant-negative activity, including in the kinase domain of another cyclin-dependent kinase, CDK13.[15,16] Moreover, overexpression of the kinase-dead mutants of different CDKs (CDK1, CDK2, CDK3, or CDK9) in human

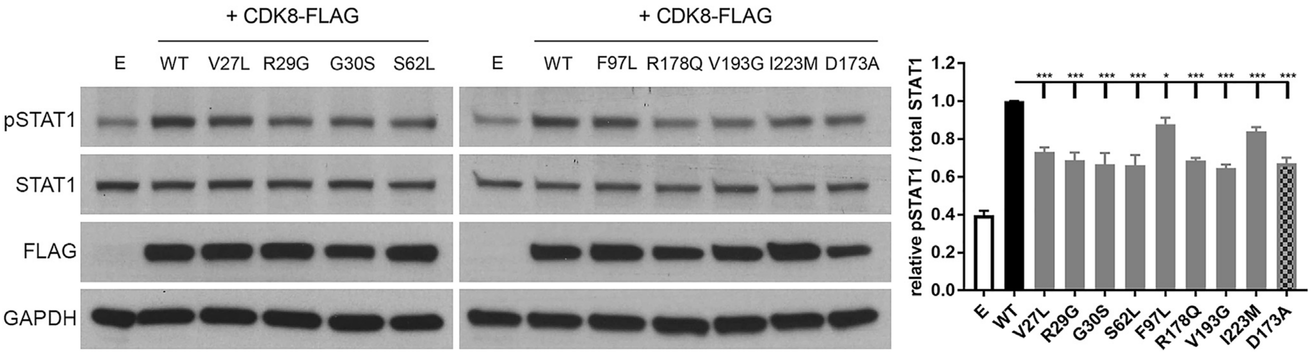

Figure 6. Effect of CDK8 Substitutions on STAT1-Ser727 Phosphorylation
SW620 *CDK8* and *CDK19* double-knockout cells were transiently transfected with FLAG-tagged wild-type (WT) or mutant *CDK8* constructs, and the cellular levels of pSTAT1-Ser727 were determined as a readout of CDK8 activity. The CDK8 kinase-dead (p.Asp173Ala) mutant was used as a control for defective kinase activity. Whole-cell lysates from non-transfected cells (E lane) or from transfected cells were collected, and equal amounts of total protein were subjected to immunoblotting with anti-phospho-STAT1-Ser727 and anti-STAT1 (total) antibodies. Anti-FLAG was used to detect the overexpressed CDK8 constructs, and GAPDH was used as loading control. Representative blots are shown in the two left panels. For the quantification (right panel), the relative intensities of the bands were taken as a ratio of the phosphorylated STAT1-Ser727 (pSTAT1) over the total amount of STAT1 (STAT1) and then plotted against the WT, which was normalized to one, for each individual blot. The values shown represent means ± SEM from five independent experiments. The results were compared to WT and analyzed by one-way ANOVA with Dunnett's multiple-comparisons test; * $p \leq 0.05$, ** $p \leq 0.01$, and *** $p \leq 0.001$.

cells was previously observed to act by dominant-negative mechanisms.[45–47] We explored the mechanism directly by mixing equal amounts of the WT and p.Ser62Leu CDK8 constructs and measuring STAT1 phosphorylation, but the observed reduction in phosphorylation in mixed samples did not obviously deviate from a linear response (data not shown); hence, further experiments will be required to support or refute the dominant-negative hypothesis.

Previous observations that mutations in *MED12* (X-linked; mostly hemizygous) and *MED13L* (heterozygous) were associated with generally similar phenotypes, including ID, hypotonia, and other congenital anomalies,[6–8,48] led to the coining of a so-called "mediatorpathy" phenotype.[49] More recently, heterozygous mutations in *MED13* were also considered to fit this pattern.[9] Interestingly, in the case of *MED12*, it was demonstrated experimentally that the substitutions p.Arg961Trp and p.Asn1007Ser (causing FG and Lujan syndromes, respectively), exhibit impaired recruitment of CDK8 onto GLI3-target gene promoters, leading to hyperactivated GLI3-dependent SHH signaling.[50] Here, we find that the major clinical features (including moderate ID, hypotonia, ASD, ADHD, and CHD) associated with *CDK8* mutations again show substantial phenotypic overlap, lending further support to the general concept of a Module-related syndrome. By contrast, relatively fewer (to date, up to four replicated: *MED17*, *MED20*, *MED23*, and *MED25*) mutations in the other 26 non-Module components of the Mediator have been described; bi-allelic mutations in *MED17* or *MED20* (both members of the Mediator's Head) produce progressive cerebral and/or cerebellar atrophy, whereas bi-allelic mutations in *MED23* or *MED25* cause syndromic or non-syndromic ID (summarized in Table S3).

In summary, therefore, our observations support the proposed clustering of developmental disorders related to perturbed function of the Module ("Mediator kinase modulopathy"). Given the pleiotropic actions of the Module, many mechanisms, including defects in key developmental signaling pathways, might lead to these pathologies. For example, the severe developmental defects identified in embryos with *Med12* hypomorphic mutations are produced by impairment of the canonical and Wnt/PCP signaling pathways;[51] analogously, CDK8 has a pivotal role as a regulator of several additional signaling pathways, including WNT, TGFβ/BMP, STAT1, SHH, and NOTCH.[52,53] Thus, a disruption of the proper activity of CDK8 could modify any of these developmental signaling networks.

Although the idea of a Mediator kinase modulopathy as a pathogenic entity is attractive, several cautions are necessary. Genetic evidence from *Drosophila* formally demonstrates the non-equivalence of different Module components, on the basis of differences in the respective phenotypes when comparing mosaic knockouts for a *Cdk8-Ccnc* pair compared with a *Med12-Med13* pair.[54] This situation is compounded for vertebrates, in which paralogous pairs exist for three of the four Module subunits. Indeed, the single described disruption (caused by a chromosomal inversion) of the *CDK8* paralog *CDK19* was associated with a different phenotype that comprised microcephaly, congenital bilateral falciform retinal folds, nystagmus, and learning disability;[55] however, the functions of CDK8 and CDK19 are known to be divergent.[56] Finally, it remains uncertain to what extent, and in which circumstances, Module activity might be kinase-independent because these functions would likely be unaffected by the mutations described here.[2] Further work on the structure and function of the Mediator will doubtless shed light on these issues.

Supplemental Data

Supplemental Data can be found with this article online at https://doi.org/10.1016/j.ajhg.2019.02.006.

Acknowledgments

We are very grateful to all the families for their participation in this study. We thank Sue Butler for cell culture, Michael Carter for technical support, staff at the Medical Research Council (MRC) Weatherall Institute of Molecular Medicine (WIMM) Sequencing facility for DNA sequencing, and Kirsty McWalter and Erin Torti (GeneDx) for their help with connecting clinical cases.

The Deciphering Developmental Disorders (DDD) study presents independent research commissioned by the Health Innovation Challenge Fund (grant number HICF-1009-003), a parallel funding partnership between Wellcome and the Department of Health, and the Wellcome Sanger Institute [grant number WT098051]. The research team acknowledges the support of the National Institute for Health Research (NIHR), through the Comprehensive Clinical Research Network. This study makes use of DECIPHER, which is funded by the Wellcome Trust. T.K. was funded by the Cancer Research UK Accelerator Award C1362/A20263. M.J.O.R., J.B., and P.A.C. acknowledge support from Cancer Research UK; A.H. for post-doctoral funding from the Institute of Cancer Research (ICR); and O.P. for funding from Merck. A.O.M.W. was supported by the NIHR Oxford Biomedical Research Centre Programme and the Wellcome Investigator Award 102731. The views expressed in this publication are those of the authors and not necessarily those of Wellcome, NIHR, or the Department of Health.

Declaration of Interests

A.H., T.K., O.P., M.J.O.R., J.B., and P.A.C. are current or former employees of The Institute of Cancer Research (ICR), which has a commercial interest in the development of WNT pathway inhibitors, and they have received awards-to-inventor payments for the discovery and development of CDK8 and CDK19 inhibitors in partnership with Merck. M.J.O.R. is currently an employee of Merck, and J.B. is currently an employee of Azeria Therapeutics and NeoPhore. K.G.M. and A.T. are employees of GeneDx, a wholly owned subsidiary of OPKO Health.

Received: December 7, 2018
Accepted: February 4, 2019
Published: March 21, 2019

Web Resources

Clustal Omega, https://www.ebi.ac.uk/Tools/msa/clustalo/
DECIPHER, https://decipher.sanger.ac.uk/
ExAC Browser, http://exac.broadinstitute.org/
GeneMatcher, https://genematcher.org/
gnomAD Browser, https://gnomad.broadinstitute.org/
MutationTaster, http://www.mutationtaster.org/
OMIM, http://www.omim.org/
PolyPhen-2, http://genetics.bwh.harvard.edu/pph2/
PubMed, https://www.ncbi.nlm.nih.gov/pubmed
RCSB Protein Data Bank (PDB), https://www.rcsb.org/
Servier Medical Art, https://smart.servier.com/
SIFT, http://sift.jcvi.org/
UniProt, https://www.uniprot.org/

References

1. Harper, T.M., and Taatjes, D.J. (2018). The complex structure and function of Mediator. J. Biol. Chem. *293*, 13778–13785.
2. Jeronimo, C., and Robert, F. (2017). The Mediator complex: At the nexus of DNA polymerase II transcription. Trends Cell Biol. *27*, 765–783.
3. Larivière, L., Seizl, M., and Cramer, P. (2012). A structural perspective on Mediator function. Curr. Opin. Cell Biol. *24*, 305–313.
4. Poss, Z.C., Ebmeier, C.C., Odell, A.T., Tangpeerachaikul, A., Lee, T., Pelish, H.E., Shair, M.D., Dowell, R.D., Old, W.M., and Taatjes, D.J. (2016). Identification of mediator kinase substrates in human cells using Cortistatin A and quantitative phosphoproteomics. Cell Rep. *15*, 436–450.
5. Clark, A.D., Oldenbroek, M., and Boyer, T.G. (2015). Mediator kinase module and human tumorigenesis. Crit. Rev. Biochem. Mol. Biol. *50*, 393–426.
6. Graham, J.M., Jr., and Schwartz, C.E. (2013). *MED12* related disorders. Am. J. Med. Genet. A. *161A*, 2734–2740.
7. Adegbola, A., Musante, L., Callewaert, B., Maciel, P., Hu, H., Isidor, B., Picker-Minh, S., Le Caignec, C., Delle Chiaie, B., Vanakker, O., et al. (2015). Redefining the MED13L syndrome. Eur. J. Hum. Genet. *23*, 1308–1317.
8. Asadollahi, R., Zweier, M., Gogoll, L., Schiffmann, R., Sticht, H., Steindl, K., and Rauch, A. (2017). Genotype-phenotype evaluation of *MED13L* defects in the light of a novel truncating and a recurrent missense mutation. Eur. J. Med. Genet. *60*, 451–464.
9. Snijders Blok, L., Hiatt, S.M., Bowling, K.M., Prokop, J.W., Engel, K.L., Cochran, J.N., Bebin, E.M., Bijlsma, E.K., Ruivenkamp, C.A.L., Terhal, P., et al.; DDD study (2018). *De novo* mutations in *MED13*, a component of the Mediator complex, are associated with a novel neurodevelopmental disorder. Hum. Genet. *137*, 375–388.
10. Sobreira, N., Schiettecatte, F., Valle, D., and Hamosh, A. (2015). GeneMatcher: A matching tool for connecting investigators with an interest in the same gene. Hum. Mutat. *36*, 928–930.
11. Mcrae, J.F., Clayton, S., Fitzgerald, T.W., Kaplanis, J., Prigmore, E., Rajan, D., Sifrim, A., Aitken, S., Akawi, N., Alvi, M., et al.; Deciphering Developmental Disorders Study (2017). Prevalence and architecture of *de novo* mutations in developmental disorders. Nature *542*, 433–438.
12. Lek, M., Karczewski, K.J., Minikel, E.V., Samocha, K.E., Banks, E., Fennell, T., O'Donnell-Luria, A.H., Ware, J.S., Hill, A.J., Cummings, B.B., et al.; Exome Aggregation Consortium (2016). Analysis of protein-coding genetic variation in 60,706 humans. Nature *536*, 285–291.
13. Samocha, K.E., Kosmicki, J.A., Karczewski, K.J., Donnell-Luria, A.H., Pierce-Hoffman, E., MacArthur, D.G., Neale, B.M., and Daly, M.J. (2017). Regional missense constraint improves variant deleteriousness prediction. bioRxiv *148353*, org/10.1101/148353.
14. Quinodoz, M., Royer-Bertrand, B., Cisarova, K., Di Gioia, S.A., Superti-Furga, A., and Rivolta, C. (2017). DOMINO: Using machine learning to predict genes associated with dominant disorders. Am. J. Hum. Genet. *101*, 623–629.
15. Hamilton, M.J., Caswell, R.C., Canham, N., Cole, T., Firth, H.V., Foulds, N., Heimdal, K., Hobson, E., Houge, G., Joss, S., et al. (2018). Heterozygous mutations affecting the protein kinase domain of *CDK13* cause a syndromic form of

developmental delay and intellectual disability. J. Med. Genet. *55*, 28–38.

16. Lelieveld, S.H., Wiel, L., Venselaar, H., Pfundt, R., Vriend, G., Veltman, J.A., Brunner, H.G., Vissers, L.E.L.M., and Gilissen, C. (2017). Spatial clustering of *de novo* missense mutations identifies candidate neurodevelopmental disorder-associated genes. Am. J. Hum. Genet. *101*, 478–484.
17. Jin, S.C., Homsy, J., Zaidi, S., Lu, Q., Morton, S., DePalma, S.R., Zeng, X., Qi, H., Chang, W., Sierant, M.C., et al. (2017). Contribution of rare inherited and *de novo* variants in 2,871 congenital heart disease probands. Nat. Genet. *49*, 1593–1601.
18. Schneider, E.V., Böttcher, J., Huber, R., Maskos, K., and Neumann, L. (2013). Structure-kinetic relationship study of CDK8/CycC specific compounds. Proc. Natl. Acad. Sci. USA *110*, 8081–8086.
19. Baumli, S., Lolli, G., Lowe, E.D., Troiani, S., Rusconi, L., Bullock, A.N., Debreczeni, J.E., Knapp, S., and Johnson, L.N. (2008). The structure of P-TEFb (CDK9/cyclin T1), its complex with flavopiridol and regulation by phosphorylation. EMBO J. *27*, 1907–1918.
20. Chemical Computing Group (2018). Molecular Operating Environment (MOE) 2015.10.
21. Hanks, S.K., and Hunter, T. (1995). Protein kinases 6. The eukaryotic protein kinase superfamily: kinase (catalytic) domain structure and classification. FASEB J. *9*, 576–596.
22. Torkamani, A., Kannan, N., Taylor, S.S., and Schork, N.J. (2008). Congenital disease SNPs target lineage specific structural elements in protein kinases. Proc. Natl. Acad. Sci. USA *105*, 9011–9016.
23. Hemmer, W., McGlone, M., Tsigelny, I., and Taylor, S.S. (1997). Role of the glycine triad in the ATP-binding site of cAMP-dependent protein kinase. J. Biol. Chem. *272*, 16946–16954.
24. Grant, B.D., Hemmer, W., Tsigelny, I., Adams, J.A., and Taylor, S.S. (1998). Kinetic analyses of mutations in the glycine-rich loop of cAMP-dependent protein kinase. Biochemistry *37*, 7708–7715.
25. Okur, V., Cho, M.T., Henderson, L., Retterer, K., Schneider, M., Sattler, S., Niyazov, D., Azage, M., Smith, S., Picker, J., et al. (2016). *De novo* mutations in *CSNK2A1* are associated with neurodevelopmental abnormalities and dysmorphic features. Hum. Genet. *135*, 699–705.
26. Schneider, E.V., Böttcher, J., Blaesse, M., Neumann, L., Huber, R., and Maskos, K. (2011). The structure of CDK8/CycC implicates specificity in the CDK/cyclin family and reveals interaction with a deep pocket binder. J. Mol. Biol. *412*, 251–266.
27. Cholko, T., Chen, W., Tang, Z., and Chang, C.A. (2018). A molecular dynamics investigation of CDK8/CycC and ligand binding: Conformational flexibility and implication in drug discovery. J. Comput. Aided Mol. Des. *32*, 671–685.
28. Mondal, J., Tiwary, P., and Berne, B.J. (2016). How a kinase inhibitor withstands gatekeeper residue mutations. J. Am. Chem. Soc. *138*, 4608–4615.
29. Zuccotto, F., Ardini, E., Casale, E., and Angiolini, M. (2010). Through the “gatekeeper door”: Exploiting the active kinase conformation. J. Med. Chem. *53*, 2681–2694.
30. Bancerek, J., Poss, Z.C., Steinparzer, I., Sedlyarov, V., Pfaffenwimmer, T., Mikulic, I., Dölken, L., Strobl, B., Müller, M., Taatjes, D.J., and Kovarik, P. (2013). CDK8 kinase phosphorylates transcription factor STAT1 to selectively regulate the interferon response. Immunity *38*, 250–262.
31. Clarke, P.A., Ortiz-Ruiz, M.J., TePoele, R., Adeniji-Popoola, O., Box, G., Court, W., Czasch, S., El Bawab, S., Esdar, C., Ewan, K., et al. (2016). Assessing the mechanism and therapeutic potential of modulators of the human Mediator complex-associated protein kinases. eLife *5*, e20722.
32. Dale, T., Clarke, P.A., Esdar, C., Waalboer, D., Adeniji-Popoola, O., Ortiz-Ruiz, M.J., Mallinger, A., Samant, R.S., Czodrowski, P., Musil, D., et al. (2015). A selective chemical probe for exploring the role of CDK8 and CDK19 in human disease. Nat. Chem. Biol. *11*, 973–980.
33. Koehler, M.F.T., Bergeron, P., Blackwood, E.M., Bowman, K., Clark, K.R., Firestein, R., Kiefer, J.R., Maskos, K., McCleland, M.L., Orren, L., et al. (2016). Development of a potent, specific CDK8 kinase inhibitor which phenocopies *CDK8/19* knockout cells. ACS Med. Chem. Lett. *7*, 223–228.
34. Putz, E.M., Gotthardt, D., Hoermann, G., Csiszar, A., Wirth, S., Berger, A., Straka, E., Rigler, D., Wallner, B., Jamieson, A.M., et al. (2013). CDK8-mediated STAT1-S727 phosphorylation restrains NK cell cytotoxicity and tumor surveillance. Cell Rep. *4*, 437–444.
35. Knuesel, M.T., Meyer, K.D., Donner, A.J., Espinosa, J.M., and Taatjes, D.J. (2009). The human CDK8 subcomplex is a histone kinase that requires Med12 for activity and can function independently of mediator. Mol. Cell. Biol. *29*, 650–661.
36. Knuesel, M.T., Meyer, K.D., Bernecky, C., and Taatjes, D.J. (2009). The human CDK8 subcomplex is a molecular switch that controls Mediator coactivator function. Genes Dev. *23*, 439–451.
37. Brandler, W.M., and Sebat, J. (2015). From *de novo* mutations to personalized therapeutic interventions in autism. Annu. Rev. Med. *66*, 487–507.
38. Twigg, S.R.F., and Wilkie, A.O.M. (2015). A genetic-pathophysiological framework for craniosynostosis. Am. J. Hum. Genet. *97*, 359–377.
39. Surosky, R.T., Strich, R., and Esposito, R.E. (1994). The yeast UME5 gene regulates the stability of meiotic mRNAs in response to glucose. Mol. Cell. Biol. *14*, 3446–3458.
40. Liao, S.M., Zhang, J., Jeffery, D.A., Koleske, A.J., Thompson, C.M., Chao, D.M., Viljoen, M., van Vuuren, H.J., and Young, R.A. (1995). A kinase-cyclin pair in the RNA polymerase II holoenzyme. Nature *374*, 193–196.
41. Allen, B.L., and Taatjes, D.J. (2015). The Mediator complex: A central integrator of transcription. Nat. Rev. Mol. Cell Biol. *16*, 155–166.
42. Firestein, R., Bass, A.J., Kim, S.Y., Dunn, I.F., Silver, S.J., Guney, I., Freed, E., Ligon, A.H., Vena, N., Ogino, S., et al. (2008). *CDK8* is a colorectal cancer oncogene that regulates beta-catenin activity. Nature *455*, 547–551.
43. Westerling, T., Kuuluvainen, E., and Mäkelä, T.P. (2007). *Cdk8* is essential for preimplantation mouse development. Mol. Cell. Biol. *27*, 6177–6182.
44. Wilkie, A.O.M. (1994). The molecular basis of genetic dominance. J. Med. Genet. *31*, 89–98.
45. Meikrantz, W., and Schlegel, R. (1996). Suppression of apoptosis by dominant negative mutants of cyclin-dependent protein kinases. J. Biol. Chem. *271*, 10205–10209.
46. Salerno, D., Hasham, M.G., Marshall, R., Garriga, J., Tsygankov, A.Y., and Graña, X. (2007). Direct inhibition of CDK9 blocks HIV-1 replication without preventing T-cell activation in primary human peripheral blood lymphocytes. Gene *405*, 65–78.

47. van den Heuvel, S., and Harlow, E. (1993). Distinct roles for cyclin-dependent kinases in cell cycle control. Science *262*, 2050–2054.
48. Asadollahi, R., Oneda, B., Sheth, F., Azzarello-Burri, S., Baldinger, R., Joset, P., Latal, B., Knirsch, W., Desai, S., Baumer, A., et al. (2013). Dosage changes of *MED13L* further delineate its role in congenital heart defects and intellectual disability. Eur. J. Hum. Genet. *21*, 1100–1104.
49. Caro-Llopis, A., Rosello, M., Orellana, C., Oltra, S., Monfort, S., Mayo, S., and Martinez, F. (2016). *De novo* mutations in genes of mediator complex causing syndromic intellectual disability: Mediatorpathy or transcriptomopathy? Pediatr. Res. *80*, 809–815.
50. Zhou, H., Spaeth, J.M., Kim, N.H., Xu, X., Friez, M.J., Schwartz, C.E., and Boyer, T.G. (2012). *MED12* mutations link intellectual disability syndromes with dysregulated GLI3-dependent Sonic Hedgehog signaling. Proc. Natl. Acad. Sci. USA *109*, 19763–19768.
51. Rocha, P.P., Scholze, M., Bleiss, W., and Schrewe, H. (2010). Med12 is essential for early mouse development and for canonical Wnt and Wnt/PCP signaling. Development *137*, 2723–2731.
52. Philip, S., Kumarasiri, M., Teo, T., Yu, M., and Wang, S. (2018). Cyclin-Dependent Kinase 8: A new hope in targeted cancer therapy? J. Med. Chem. *61*, 5073–5092.
53. Poss, Z.C., Ebmeier, C.C., and Taatjes, D.J. (2013). The Mediator complex and transcription regulation. Crit. Rev. Biochem. Mol. Biol. *48*, 575–608.
54. Loncle, N., Boube, M., Joulia, L., Boschiero, C., Werner, M., Cribbs, D.L., and Bourbon, H.M. (2007). Distinct roles for Mediator Cdk8 module subunits in *Drosophila* development. EMBO J. *26*, 1045–1054.
55. Mukhopadhyay, A., Kramer, J.M., Merkx, G., Lugtenberg, D., Smeets, D.F., Oortveld, M.A.W., Blokland, E.A.W., Agrawal, J., Schenck, A., van Bokhoven, H., et al. (2010). *CDK19* is disrupted in a female patient with bilateral congenital retinal folds, microcephaly and mild mental retardation. Hum. Genet. *128*, 281–291.
56. Tsutsui, T., Fukasawa, R., Tanaka, A., Hirose, Y., and Ohkuma, Y. (2011). Identification of target genes for the CDK subunits of the Mediator complex. Genes Cells *16*, 1208–1218.
57. Lahiry, P., Torkamani, A., Schork, N.J., and Hegele, R.A. (2010). Kinase mutations in human disease: interpreting genotype-phenotype relationships. Nat. Rev. Genet. *11*, 60–74.

The American Journal of Human Genetics, Volume *104*

Supplemental Data

De Novo Missense Substitutions in the Gene Encoding CDK8, a Regulator of the Mediator Complex, Cause a Syndromic Developmental Disorder

Eduardo Calpena, Alexia Hervieu, Teresa Kaserer, Sigrid M.A. Swagemakers, Jacqueline A.C. Goos, Olajumoke Popoola, Maria Jesus Ortiz-Ruiz, Tina Barbaro-Dieber, Lucy Bownass, Eva H. Brilstra, Elise Brimble, Nicola Foulds, Theresa A. Grebe, Aster V.E. Harder, Melissa M. Lees, Kristin G. Monaghan, Ruth A. Newbury-Ecob, Kai-Ren Ong, Deborah Osio, Francis Jeshira Reynoso Santos, Maura R.Z. Ruzhnikov, Aida Telegrafi, Ellen van Binsbergen, Marieke F. van Dooren, The Deciphering Developmental Disorders Study, Peter J. van der Spek, Julian Blagg, Stephen R.F. Twigg, Irene M.J. Mathijssen, Paul A. Clarke, and Andrew O.M. Wilkie

Supplemental Note: Case Reports

Subject 1

This girl was born after an uneventful pregnancy. Immediately after birth a ptosis of the right eye was noticed. During the first year of life feeding difficulties, a vertical gaze palsy, hypotonia and motor delay became apparent. Pediatric neurological examination also showed facial weakness and a congenital myasthenic syndrome was suspected. A pyridostigmine treatment trial resulted in a mild, temporary improvement.

Language development was also delayed, with nasal speech and a hoarse voice. She has a sensorineural hearing loss (right: 35 dB, left: 22 dB). Because of a mild intellectual disability (ID), visual impairment and attention deficit hyperactivity disorder (ADHD), she visits a special educational needs school. Behavioral problems include ADHD, tantrums and anxiety. Until the age of 6 yr she has had recurrent febrile episodes.

Array-CGH was normal. Clinical whole exome sequencing (WES; performed in-house as a parent-child trio) did not identify any known pathogenic variants; however the *de novo* c.79G>C (p.Val27Leu) substitution in *CDK8* was highlighted as a variant of unknown significance (VUS) and submitted to GeneMatcher.

An additional *de novo* missense variant was identified in the *CACNA1A* gene (gnomAD: missense Z = 6.19, o/e = 0.55; loss-of-function [LoF] pLI = 1.00, o/e = 0.05). The *CACNA1A* NM_023035.2:c.2570G>A (p.Arg857His) variant is not present in gnomAD. Mutations affecting the *CACNA1A* gene are responsible for the autosomal dominant epileptic encephalopathy, early infantile (MIM: 617106), episodic ataxia, type 2 (MIM: 108500), migraine, familial hemiplegic (MIM: 141500) or spinocerebellar ataxia 6 (MIM: 183086) disorders. This variant has not been identified before in any of these disease cohorts. Although the missense variant is located outside the intervals of the transmembrane domains (I-IV), in a region apparently more tolerant to missense variation,[1] we cataloged this as a VUS.

Additionally, a *de novo* mosaic variant (present in ~20% of cells) was identified in the *HDAC9* gene (gnomAD: missense Z = 2.27, o/e = 0.73; LoF pLI = 1.00, o/e = 0.13).

The *HDAC9* NM_178423.1:c.646C>T (p.Arg216*) variant is not present in gnomAD. Although it was suggested that *HDAC9* deletions could represent a risk factor for autism, ID and schizophrenia associated with incomplete penetrance/variable expressivity,[2] the involvement of *HDAC9* mutations in congenital disorders has not been defined.

Subject 2

This 3-year old male was born at 37+5/7 weeks by forceps-assisted vaginal delivery after an uncomplicated pregnancy. Apgar scores were 7 and 8 at 1 and 5 min, respectively. The neonatal course was significant for respiratory distress thought to be secondary to meconium aspiration, hyperbilirubinemia requiring phototherapy, and feeding difficulty. He did not pass his newborn hearing screen, which was initially thought to be related to antibiotics given as part of sepsis rule-out during his neonatal intensive care unit (NICU) course.

At 7 weeks of age, he was admitted for failure-to-thrive. He also had increased respiratory effort during feeds as well as oral motor difficulties, reflux, and inefficient suck. He was found to have laryngomalacia and a sleep study demonstrated severe obstructive sleep apnea requiring supplemental oxygen. A nasogastric tube was also placed for supplemental nutrition. During this hospitalization he was noted to have low tone and subtle dysmorphic features prompting further work-up for a neurologic and genetic disorder. Metabolic screening (plasma amino acids, urine organic acids, acylcarnitine profile, lactic acid and creatine kinase) was unrevealing, as were a single nucleotide polymorphism (SNP) chromosomal microarray and methylation studies for Prader-Willi and Angelman syndromes. Magnetic resonance imaging (MRI) of the brain at 5 mo showed nonspecific white matter abnormalities including a thin corpus callosum with bilateral posterior supratentorial and perihippocampal white matter volume loss, with possible hypomyelination.

He went on to demonstrate significant delays in his development in all domains. An evaluation at 12-13 mo showed age equivalencies of 3-4 mo for gross and fine motor skills, and 6-7 mo for social development. At the most recent assessment (aged 3 yr) he was able to roll and hold his head up, as well as sit propped up in a chair but not

unsupported. He could reach for toys and grasp with a full hand but had no pincer grasp. He says “mama” and “papa” but no other words, he responds to his name and tracks well. On general examination he is dysmorphic with brachycephaly and a tall and broad forehead, shallow orbits with the appearance of proptosis, low set ears with overfolded superior helices bilaterally and a tented mouth that is held open (low facial tone). His growth parameters are all within the normal range. Neurologic exam showed an alert toddler with no verbal output, good tracking and attention to sound. He was able to follow some simple commands from parents with prompting. He has marked global hypotonia with normal reflexes and no clonus, as well as frequent non-purposeful complex stereotypic movements of his distal extremities.

He is currently followed by multiple specialties, including ophthalmology for nystagmus, high myopia, and optic nerve cupping of both eyes; neurology; audiology for sensorineural hearing loss; and an aerodigestive team for feeding (he now has a G tube in place for nutrition) as well as respiratory concerns. He no longer requires oxygen and has had some improvement in swallowing, however aspiration continues to be a risk. He has had a normal echocardiogram.

As part of his diagnostic work-up, trio whole exome sequencing was performed by GeneDx, which identified a *de novo* missense variant of uncertain significance in the gene *CDK8* (c.85C>G, p.Arg29Gly).

Subject 3

This 9-year old boy was born as the second child, after an uneventful pregnancy of non-consanguineous parents, with a severe trigonocephaly, a perimembranous ventricular septal defect with a double orifice mitral valve, and pyloric hypertrophy for which surgery was required. Measurements of thyroid function were normal. No intracranial anomalies were seen on the preoperative CT scan and an ultrasound of the kidneys was normal. An uneventful fronto-supraorbital reconstruction was performed at 10 mo.

His development was delayed and he started walking at age 2.5 yr and talking at age 4 yr. At 8 yr an IQ of 55 was measured; his social behavior was like a 12- to 18-month old. He had trouble going to sleep for which melatonin was used. The diagnosis of

autism spectrum disorder (ASD) and of ADHD was made; the ADHD worsened in response to Ritalin and was thus stopped. On ophthalmological review he had intermittent exotropia and surso adductorius in both eyes with V-motility. Vision was 0.5 for both eyes but somewhat unreliable due to a lack of concentration.

Whole genome sequencing (WGS), performed on the parent-child trio as part of a research study of craniosynostosis, did not identify any known pathogenic variants. Three *de novo* substitutions were identified, of which the c.88G>A (p.Gly30Ser) mutation in *CDK8* was selected for further investigation.

Two additional *de novo* missense variants were identified in the *TRPM2* (gnomAD: missense Z = -0.21, o/e = 1.02; LoF pLI = 0.00, o/e = 0.89) and *COL3A1* (gnomAD: missense Z = 4.40, o/e = 0.59; LoF pLI = 1.00, o/e = 0.04) genes. The *TRPM2* NM_003307:c.2557G>A (p.Glu853Lys) variant has been observed in gnomAD (6/281772, 2.129e-5). Additionally, in gnomAD there are additional variants affecting the same amino acid (p.Glu853Gln, p.Glu853Asp). We considered the variant unlikely to have clinical significance. The *COL3A1* NM_000090, c.2621T>C (p.Phe874Ser) variant is not present in gnomAD, although there is a variant affecting the same amino acid (p.Phe874Cys). Mutations affecting the *COL3A1* gene are responsible for the autosomal dominant Ehlers-Danlos syndrome, vascular type (MIM: 130050). This variant has not been identified before in Ehlers-Danlos syndrome cohorts. Although this missense variant differs from the typical signature of causative variants identified in Ehlers-Danlos syndrome,[3] we cataloged this as a VUS.

Subject 4

This girl, currently aged 7.6 yr, was born to a 38 year old G2 P1 mother. The pregnancy was complicated by pneumonia, for which she took antibiotics. The prenatal ultrasound revealed a hypoplastic left heart and agenesis of the corpus callosum. She was born by caesarean section at 38 weeks’ gestation, and was small for gestational age (birthweight 2.3 kg, length 47 cm). She was cyanotic and hypoxic at birth, and underwent cardiac surgery at 4 days of age via a Norwood procedure. She has subsequently undergone two additional cardiac surgeries, a bi-directional Glenn procedure aged 4 mo and a Fontan procedure aged 4 yr. She had dysphagia requiring a gastrostomy tube aged 7 mo, but now eats completely by mouth. She wears glasses

for myopia and has normal hearing. A brain magnetic resonance imaging (MRI) confirmed agenesis of the corpus callosum and mild ventriculomegaly.

She has a history of failure to thrive and remains small for her age, with her height at -2.53 SD, weight at -1.92 SD, and a normal occipito-frontal circumference (OFC). She is mildly dysmorphic, with bilateral epicanthal folds, prominent eyes, a small nasal tip with a short columella and a broad nasal base, broad thumbs and broad, laterally deviated great toes, hypoplastic distal phalanges of all toes, planovalgus feet, and mild hypotonia.

Her development has been globally delayed. She did not roll or sit until 18 mo, walked at 4 ½ yr, said her first word at 4 yr, and spoke in sentences at 6 yr. She is in special education classes at school and receives speech, physical, occupational, developmental and music therapies. She has ADHD, with a sweet personality, but is sometimes aggressive, and has a severe sleep disorder.

Clinical WES was performed on the parent-child trio by GeneDx and did not identify any known pathogenic variants; however the *de novo* c.185C>T (p.Ser62Leu) substitution in *CDK8* was reported as a VUS and submitted to GeneMatcher.

Subject 5

This individual is a 2-day old boy born after an uneventful pregnancy at 41+0 weeks' gestation via vaginal delivery after induced labor. He is the second child of unrelated parents. At birth he weighed 3120 grams. He was born with an ano-rectal malformation, an atrial septal defect, ventricular septal defect (VSD), bicuspid aortic valve, and a hypoplastic aortic arch. On examination no dysmorphic features were present except for low-set ears, which were slightly rotated backwards. He was lost to follow-up at the age of 6 mo, at which time he was unable to sit independently.

SNP-array showed a paternally inherited duplication at 7q11.21 (chr7:63,352,782-63,856,505; hg19)x3 considered likely to be a benign finding. Clinical WES was performed on the parent-child trio at the same institution as for Subject 1. Although this did not identify any known pathogenic variants, the *de novo* c.185C>T (p.Ser62Leu) substitution in CDK8 was highlighted as being of potential significance.

An additional *de novo* missense variant was identified in the *TRAF6* gene (gnomAD: missense Z = 2.85, o/e = 0.54; LoF pLI = 1.00, o/e = 0.05). The *TRAF6* NM_004620.3: c.1193C>T (p.Pro398Leu) variant is not present in gnomAD. The variant does not affect any of the three zinc finger domains, although is located within a region that is likely involved in protein interactions. The implication of mutations of *TRAF6* in congenital disorders is not well defined so far. A *de novo* LoF (frameshift mutation located in the last exon of the *TRAF6*) was described in one proband with hypohidrotic ectodermal dysplasia,[4] whereas a homozygous deletion affecting the 5′UTR of *TRAF6* was recently identified in a subject with an atypical form of osteopetrosis.[5] We cataloged the identified variant as a VUS.

Subject 6

This individual is a 10 mo female, born to white parents aged 28 yr (mother) and 39 yr (father), and was initially referred for clinical genetics evaluation at the age of 5 mo owing to agenesis of the corpus callosum, aortic coarctation and global developmental delay. She has one healthy older sister. The mother had two previous miscarriages. On examination she had a large head (occipito-frontal circumference [OFC] 47.5 cm, +2.21 SD), with hypertelorism, sparse eyebrows, long columella and downturned corners of the mouth, with axial hypotonia and hypertonia of the lower extremities. Initial workup included a normal karyotype and SNP array. She underwent aortic coarctation repair aged 5 mo with a good result. When she was last evaluated at the age of 10 mo she had made some developmental progress, but was significantly delayed with milestones equivalent to 5 mo (rolling over, sitting with support).

Clinical WES was performed on the parent-child trio by GeneDx and did not identify any known pathogenic variants; however the *de novo* c.185C>T (p.Ser62Leu) substitution in *CDK8* was highlighted as a VUS and submitted to GeneMatcher.

Subject 7

This male subject is the only child of his parents. At birth he was noted to have low set ears, redundant skin in the neck, lymphedema of the hands and feet, undescended testes, and echocardiography showed tetralogy of Fallot. An initial diagnosis of

Noonan Syndrome was proposed. He underwent cardiac surgery at 9 mo. Assessment at 7 mo demonstrated mild delay of motor development and he continued to have low muscle tone throughout childhood. By 8 yr he had developed complex partial seizures and migraines, both improved with valproic acid. He had mild-moderate ID and was in special educational classes.

Genetic testing including array-comparative genomic hybridization (array-CGH), *CHD7* and *KAT6B* did not reveal any pathogenic mutations.

Assessment at the age of 16 yr demonstrated persistence of low muscle tone, including low oral muscle tone with dribbling. He had an abnormal narrow palate with marked dental decay. He has had bilateral lateral column lengthening and serial plaster casting for *pes planus* and can walk for approximately 2 miles. He is registered as partially sighted due to a severe myopia. He attended school for children with special educational needs. At the age of 22 yr he was given a diagnosis of childhood autism.

He was enrolled as a singleton into the Deciphering Developmental Disorders (DDD) WES project, which did not identify any pathogenic variants of known clinical significance. The heterozygous c.185C>T (p.Ser62Leu) variant in *CDK8* was identified through DDD CAP#144 and subsequently confirmed on dideoxy-sequencing to be present in the subject and absent in his mother. The father was unavailable for analysis.

Subject 8

This child is the second of two boys born to unrelated parents. The older brother has epilepsy but no other medical problems. The father (who was not available for genetic testing) is well; the mother, who has moderate learning difficulties, tested negative for the *CDK8* c.185C>T variant identified in her son; there is no other family history of learning problems.

He was born after an uneventful pregnancy at 41 weeks' gestation with a birth weight of 4.3 kg. No neonatal problems were reported. He was seen by a pediatrician at 5 mo because of developmental delay, marked hypotonia and brachycephaly with marked flattening over the right occiput. At almost 2 yr, he had marked head lag when lifted

from supine and could roll over. He was dysmorphic with down-slanting palpebral fissures, slightly low set right ear and alternating divergent squint. Growth measurements were normal. Past investigations included normal karyotype, Fragile X and basic metabolic screening. He had a brain MRI which showed agenesis of the corpus callosum.

At the age of almost 4 yr he had generalized hypotonia (deep tendon reflexes in the upper and the lower limbs were not elicited; plantars down going) and positional kyphosis. He had limited communication skills (single words only). He was mainly transported in a buggy but was able to use a standing frame for about 45 minutes a day. Since early childhood he had an anemia of unknown origin, which improved with iron supplements.

At the age of 5.5 yr he was reviewed in the genetics clinic. He still had mild plagiocephaly. Hypotonia was improving; he had significant generalized joint laxity. Aged 7 yr he had a microarray which showed a maternally inherited duplication at Xq21.31 (chrX:86315153-86578362; hg18) containing no RefSeq genes within the minimum region and considered to be an innocent finding.

At the last genetic review aged 12.7 yr his problems were global developmental impairment, ASD and ADHD, dysmorphic features and plagiocephaly, absent corpus callosum, generalized hypotonia, gastro-esophageal reflux with food regurgitation, recurrent loose stool and intermittent rectal mucosal prolapse, strabismus (wears corrective glasses). His height and weight were between the 2nd and 9th centile. He is regularly seen by the child adolescent mental health service. He attends a SEN school and due to social issues he is a child in care.

He was enrolled as a singleton into the DDD project, which did not identify any pathogenic variants of known clinical significance. The heterozygous c.185C>T (p.Ser62Leu) variant in *CDK8* was identified through DDD CAP#144 and subsequently confirmed on dideoxy-sequencing to be present in the subject and absent in his mother.

Subject 9

She is the 5th child of healthy, unrelated parents and was born at term following a normal pregnancy. She first came to medical attention at a few days of age with paroxysmal events consisting of eye rolling and limb jerking. These events were captured during an electroencephalogram (EEG) recording and were not felt to be epileptic. She had failed her neonatal hearing screen and was diagnosed with sensorineural hearing loss; profound on the right and moderate on the left. MRI brain examination at the time was normal other than some hippocampal asymmetry.

Developmental delay was evident from 4 mo. A diagnosis of a seizure disorder was made by one year of age and Lamotrigine treatment started. A repeat MRI scan showed no progression of the previously observed changes but noted incomplete myelination. An additional diagnosis of a movement disorder consisting of tremor and dystonia was made by 4 yr. She continues with epileptic and non-epileptic paroxysmal events that are exacerbated by pyrexia. Investigations into her movement disorder have shown a low cerebrospinal fluid (CSF) homovanillic acid level, the significance of which is unclear. At 8 yr she has moderate delay in all developmental areas and a diagnosis of autism. She is well grown (OFC 25th centile), normally proportioned and has a facial appearance in keeping with that of her family. She exhibits abnormal posturing of all four limbs, an overt tremor of her upper limbs, but has normal power and deep tendon reflexes. She has mild peripheral joint hypermobility, but normal skin texture and elasticity.

Array-CGH was normal. She was enrolled with her parents into the DDD and 100,000 Genomes (WGS) projects, neither of which identified pathogenic variants of known clinical significance. The *de novo* c.291T>G (p.Phe97Leu) variant in *CDK8* was identified through DDD CAP#144.

Subject 10

He was born at term following an unremarkable pregnancy. He is the third child of healthy unrelated parents. He had mild hyperbilirubinemia as a baby that did not require any treatment. There were no developmental concerns prior to the age of 1 year.

He was first assessed by the clinical genetics team at 5 yr, by which time he had global developmental delay and acquired microcephaly (OFC centiles at 3 mo on the 50th, at 2 yr on the 25th and at 5 yr on the 2nd). He walked from around 2 yr, but remained unsteady and had a few single words of speech only with no ability to use Makaton or PECS cards. His receptive language abilities were a little better than this – assessed at the 24 mo level. His interactive and social skills had been assessed as at the 36 mo level. Other than his head circumference his general growth was in keeping with that of his family. He had generally been healthy, had a normal sleep pattern and there were no concerns regarding his hearing or vision.

On examination he was well-proportioned with an unsteady broad-based gait. He was very active and had a friendly personality. He had a normal skull shape, his eyes were large and he had an open mouthed expression with mild prognathism and wide-spaced teeth. His hands, feet and genitalia were normal. Skull radiographs did not show any evidence of craniosynostosis.

Array-CGH showed a 220 kb deletion on chromosome 21q22.3 (chr21:44779709-45001192, hg18) which was not felt to be of clinical significance. Methylation-specific analysis excluded common mechanisms of Angelman syndrome. DDD WES (performed as a singleton) identified three heterozygous variants in Developmental Disorders Genotype-to-Phenotype Database (DDG2P) genes (*AFF* c.1757G>A, p.Ser586Asn; *LRP5* c.2900C>T, p.Pro967Leu; and *SETBP1* c.3022C>A, p.Arg1008Ser), all of which were shown to have been inherited from one or other parent and not considered to be significant. The c.533G>A (p.Arg178Gln) variant in *CDK8* was identified through DDD CAP#144, and confirmed by dideoxy-sequencing to be present in the subject and absent in both parents. Correct sample relationships were confirmed by analysis of 13 microsatellite markers.

Subject 11

This girl is the first child born to healthy, unrelated Sri Lankan parents. There was no family history of note. The pregnancy was uncomplicated. Birth weight was 2.6 kg at 38 weeks' gestation. She was readmitted at 1 week of age because of hyperbilirubinemia, which needed treatment with phototherapy and an exchange transfusion. This was attributed at the time to breast milk jaundice. She was noted to

have a laryngeal stridor early in life; this resolved spontaneously after 1-2 yr. She was delayed in reaching her milestones, sitting at around 9-10 mo, and walking at around 18 mo. She learned to ride a bike with stabilizers at the age of 6 yr.

At the age of 3 yr she developed cyclical vomiting. This problem has persisted, and aged 12 yr she continues to be treated with flunarazine. She also takes salbutamol as required for treatment of asthma. She has had grommets inserted on two occasions for bilateral glue ear.

Academically she is making reasonable progress, with a particular strength in reading. She has an excellent memory. At school she is good at history, geography and English literature, but struggles in maths. She does have some specific learning difficulties; at the age of 12 yr she is unable to draw a straight line using a ruler, and has difficulties drawing a table or graph. These skills are felt to be around 5 yr behind her chronological age.

She is described as having a volatile personality. She can become easily disappointed if her requests are not met, and can be verbally aggressive at times; this tends to occur at home and be directed towards immediate family. At other times she is described as being sweet and charming. She can be overtalkative; non-verbal clues are often not understood. Anxiety can be an issue. Whilst she is not particularly hyperactive, and her attention span is reasonable when undertaking leisure activities such as reading, she is described as having some features compatible with ADHD. When she was younger her sleep was very restless, but she now sleeps well, although struggles to wake in the morning.

Socially, she has several long term friends. She loves small babies and wants to be a kindergarten teacher.

She was enrolled with her parents into the DDD (WES) project, which did not identify any pathogenic variants of known clinical significance. The *de novo* c.578T>G (p.Val193Gly) variant in *CDK8* was identified through DDD CAP#144.

Subject 12

This boy, a 3.5-year-old Armenian/Caucasian male, was referred for clinical genetics assessment secondary to history of hypotonia. His parents initially became concerned during the first year of life owing to delay in achieving his gross motor milestones. As an infant he was bottle-fed because of inability to latch onto the breast, but otherwise he had no feeding difficulties and weight gain was satisfactory.

He had been followed by the cardiology team secondary to a VSD, which has not required any surgical repair. His last echocardiogram, performed at the age of 6 mo showed one small 2 mm mid-muscular VSD and a second tiny anterior muscular VSD. He has passed his vision screening in his primary care physician's office, and his newborn hearing screen. A more recent hearing evaluation was non-contributory owing to lack of co-operation.

He was diagnosed with autism aged 3 yr by his school psychologist and is currently enrolled in a special needs academic program at the pre-kindergarten level. He receives physical therapy, speech therapy, and occupational therapy. His growth measurements are normal. He is mildly dysmorphic, with a broad mouth, long philtrum which is well-defined, broad nasal tip, slightly down-slanted palpebral fissures and prominent ears; in addition, persistent fetal finger pads bilaterally and slight internal rotation of the medial malleoli were noted. He wears ankle-foot orthoses, which help with ambulation. He demonstrates a positive Gower maneuver when moving from a seated to standing position. Serum creatine kinase was normal. Fragile X and SNP chromosome microarray testing was normal. Clinical WES was performed on the parent-child trio by GeneDx and did not identify any known pathogenic variants; however the *de novo* c.669A>G (p.Ile223Met) substitution in *CDK8* was highlighted as a VUS.

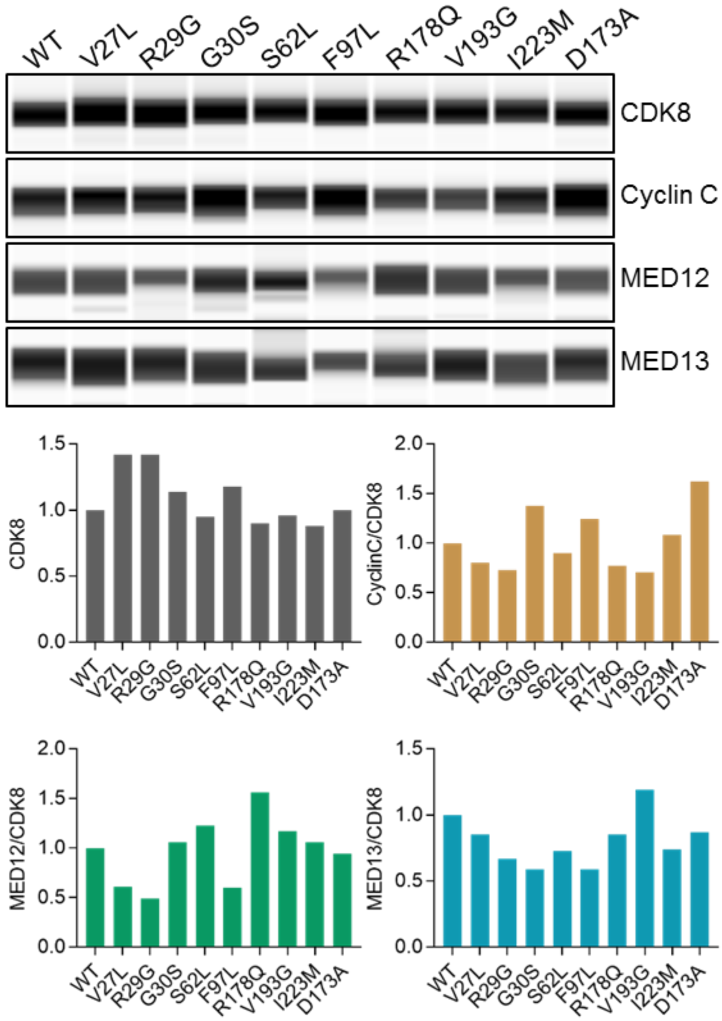

Figure S1. Detection of CDK8, cyclin C, MED12 and MED13 in the purified CDK8 Modules. Lysates from HEK293T cells transiently transfected with Myc-FLAG-tagged WT or mutant CDK8 constructs were used for immunoprecipitation using anti-FLAG antibody or mouse IgG isotype control antibody. The immunocomplexes were purified using magnetic beads with protein G. CDK8 was released by adding FLAG peptide and an aliquot of the eluted fraction was analyzed by automated capillary immunoassay system to detect CDK8, cyclin C, MED12 and MED13 (virtual blots at the top; relative quantifications from this experiment below).

	Subject 1	Subject 2	Subject 3	Subjects 4-8	Subject 9	Subject 10	Subjects 11	Subject 12
**Genomic coordinate**	chr13:26254720	chr13:26254726	chr13:26254729	chr13:26337623	chr13:26349158	chr13:26385229	chr13:26385274	chr13:26393389
**DNA variant**	c.79G>C	c.85C>G	c.88G>A	c.185C>T	c.291T>G	c.533G>A	c.578T>G	c.669A>G
**Protein change**	p.Val27Leu	p.Arg29Gly	p.Gly30Ser	p.Ser62Leu	p.Phe97Leu	p.Arg178Gln	p.Val193Gly	p.Ile223Met
**Inheritance**	*de novo*	*de novo*	*de novo*	*de novo*[a]	*de novo*	*de novo*	*de novo*	*de novo*
**MutationTaster**	disease causing	disease causing	disease causing	disease causing	disease causing	disease causing	disease causing	disease causing
**SIFT**	damaging	damaging	damaging	damaging	tolerated	damaging	damaging	damaging
**Polyphen-2**	possibly damaging	probably damaging	possibly damaging	probably damaging	benign	probably damaging	probably damaging	probably damaging

Genomic coordinates from GRCh38. Nomenclature is based on the NM_001260.2 transcript. [a]This variant was demonstrated *de novo* in subjects 4-6 and was negative in the maternal samples of individuals 7 and 8, for whom paternal samples were unavailable.

Table S1. Summary of *CDK8* mutations and analyses of pathogenicity

Primer	Sequence (5'-3')	Variants to screen
CDK8-gDNA-Exon1-F	CCTCAGAGGCTGTGACAATGG	c.79G>C (p.V27L), c.85C>G (p.R29G), c.88G>A (p.G30S)
CDK8-gDNA-Exon1-R	GCGTCGAGGAAGTCAGAGCC	
CDK8-gDNA-Exon2-F	ATCAAATAATGGGACTGGACTAGG	c.185C>T (p.S62L)
CDK8-gDNA-Exon2-R	ACAATACAGAGCCTTATATAGTTGG	
CDK8-gDNA-Exon3-F	TTTACCTGTGGGTTTGTATTGCC	c.291T>G (p.F97L)
CDK8-gDNA-Exon3-R	AATACTCTAGCCTTCGTCATGCC	
CDK8-gDNA-Exon6-F	GTAACAGTAATTTGAAGAGAATGG	c.533G>A (R178Q), c.578T>G (p.V193G)
CDK8-gDNA-Exon6-R	TTAATTAGTTCTACTCACCAATAGC	
CDK8-gDNA-Exon7-F[a]	cacgacgttgtaaaacgacGGGTGGAGGGCATGGGAATA	c.669A>G (p.I223M)
CDK8-gDNA-Exon7-R[a]	ggataacaatttcacacaggACCTCTCCAACGAAGACATCTAAGT	

[a]Primers for the exon 7 amplification include universal M13-tails (lower case).

Table S4. Primers used for validation of the genomic variants in *CDK8*

Primers for the directed mutagenesis	Forward (5'-3')	Reverse (5'-3')
CDK8-SDM-V27L	GGGCTGCAAAcTTGGCCGAGG	TCGTATTCAAACAGGTCCTC
CDK8-SDM-R29G	CAAAGTTGGCgGAGGCACTTATG	CAGCCCTCGTATTCAAAC
CDK8-SDM-G30S	AGTTGGCCGAaGCACTTATGG	TTGCAGCCCTCGTATTCAAAC
CDK8-SDM-S62L	ATCTCTATGTtGGCATGTAGAG	CCCAGTTCCTTCTATTTG
CDK8-SDM-F97L	GGCTTCTGTTgGACTATGCTG	ACACCTTCCTATCAGCATG
CDK8-SDM-R178Q	GGCTTTGCCCaATTATTTAATTC	CATGTCAGCAATTTTTACTC
CDK8-SDM-V193G	TTGGATCCAGgGGTTGTTACA	ATCTGCTAAAGGCTTCAAAG
CDK8-SDM-I223M	TAGGGTGTATgTTTGCAGAAC	TAGCCCAAATATCAATAGC
CDK8-SDM-D173A	AAAATTGCTGcCATGGGCTTTG	TACTCTTCCTCGCTCAGG
**Primers for sequencing**	**Sequence (5'-3')**	
CDK8-R1	TCGCTCAGGACCTTCACCC	
CDK8-F2	GAAGCCAGTTCAGTTACCTCG	
CDK8-F3	TGCAGCCTTATCAAGTATATGG	

Table S5. Mutagenesis and sequencing primers

Supplemental Material and Methods

Exome/genome sequencing and validation

Trio whole genome sequencing of Subject 3 was performed by Complete Genomics (Mountain View, CA, USA), as described by Drmanac et al.[6] Data were analyzed using cga tools version 1.6.0.43. A *de novo* disease model was tested, using the 'calldiff script' (Python script kindly provided by Complete Genomics) as described by Gilissen et al.[7] Prioritization of variants was based on the minimal somatic score and manual curation of the variants.

Trio exome sequencing of Subjects 1 and 5 (Utrecht cases) was performed as follows: exomes were enriched using the SureSelect XT Human All Exon V5 kit (Agilent) and sequenced in rapid run mode on the HiSeq2500 sequencing system (Illumina) at a mean target depth of 100x. The target was defined as all coding exons of UCSC and Ensembl +/- 20 bp intron flanks. At this depth ~95% of the target was covered at least 15x. Reads were aligned to hg19 using BWA (BWA-MEM v0.7.5a) and variants were called using the GATK haplotype caller (v2.7-2). Detected variants were annotated, filtered and prioritized using the Bench NGS Lab platform (Cartagenia, Leuven, Belgium). Analysis was based upon a tiered approach. The first tier analyzed known intellectual disability genes. The second tier filtered for *de novo* variants and the last tier filtered for recessive variants.

Trio exome sequencing of Subjects 2, 4, 6 and 12 (GeneDx cases) was performed as follows. Using genomic DNA from the proband and parents, the exonic regions and flanking splice junctions of the genome were captured using the Clinical Research Exome kit (Agilent Technologies, Santa Clara, CA) or the IDT xGen Exome Research Panel v1.0. Massively parallel sequencing was done on an Illumina system with 100 bp or greater paired-end reads. Reads were aligned to human genome build GRCh37/hg19, and analyzed for sequence variants using a custom-developed analysis tool. Additional details of the sequencing technology and variant interpretation protocol have been described previously.[8] The general assertion criteria for variant classification are publicly available on the GeneDx ClinVar submission page.

Exome sequencing of Subjects 7, 8 and 10 (as singletons), and 9 and 11 (as trios) was performed as part of the DDD research study.[9] Variant data were obtained as part of the approved Complementary Analysis Project #144 from Datafreeze 3, comprising 7,833 trios and 1,792 singletons with undiagnosed developmental disorders, primarily developmental delay/learning disability.

All the *CDK8* variants were validated by dideoxy-sequencing of the probands, and all available parental samples were analyzed by exome/genome and/or dideoxy-sequencing to assess inheritance of the *CDK8* mutations. In subject 10, in whom the *CDK8* variant was identified in a sample sequenced as a singleton, correct sample relationships of the trio were checked by demonstrating consistent inheritance of 13 microsatellite *loci* (*D1S2868*, *D3S1311*, *D4S403*, *D5S2027*, *D6S1610*, *D7S519*, *D9S158*, *D10S548*, *D11S898*, *D13S1265*, *D14S280*, *D16S415* and *D18S474*). The primers used for the PCR and dideoxy-sequencing validations of the genomic variants are listed in the Table S4.

Protein alignment

CDK8 protein sequences (FASTA format) from *H. sapiens* (human; P49336), *M. musculus* (mouse; Q8R3L8), *X. tropicalis* (western clawed frog; Q6P3N6), *D. rerio* (zebrafish; Q8JH47), *D. melanogaster* (fruit fly; Q9VT57), *A. aegypti* (mosquito; Q17IE8), *C. elegans* (nematode worm; P90866), and the human CDK19 protein sequence (Q9BWU1), the vertebrate paralogue of CDK8,[10] were downloaded from Uniprot and a multiple protein sequence alignment was conducted by using Clustal Omega to evaluate the conservation of the CDK8 residues mutated in the subjects of this study.

Structure preparation

A homology model of CDK8 and cyclin C was generated in MOE 2015.10[11] using the CDK8-cyclin C complex structure (PDB entry 4F7S)[12] as template and the Uniprot[13] sequence identifier P49336 (CDK8 isoform 1) and P24863 (cyclin C isoform 1) to fill in gaps in CDK8 and remove artificial N-terminal residues in cyclin C in the input structure for molecular dynamics simulation. The homology model was aligned to the

CDK9 structure (PDB entry 3BLQ)[14] and ATP and Mg^{2+} were copied into the CDK8 structure. The CDK8 complex (referring to the structure of CDK8 + ATP + Mg^{2+} + cyclin C) was prepared using the default settings of the protein preparation wizard in Maestro release 2017-2.[15] Mutations were introduced using MOE.

Simulation

ATP topologies and parameters were generated using CGenFF webserver and cgenff_charmm2gmx.py python script (MacKerell lab webserver). The protein, solvent and ions were parameterized using the July 2017 update of the CHARMM36 force field[16] in GROMACS version 2016.4.[17-23] The simulations were performed in a cubic box with a minimum distance to the box boundaries of 10 Å, solvated with TIP3P[24] waters and neutralized with chloride ions. After minimization, the system was equilibrated with 100 ps runs using both NVT and NPT ensemble where N = 138057 (CDK8 complex WT), 138059 (CDK8 complex p.Ser62Leu), 138055 (CDK8 complex p.Arg178Gln); T= 300 K; and P = 1 bar. Production simulations were run at least twice for 50 ns. If the variation between runs was large, a third run was included. Time steps of 2 fs were used and frames were saved every 10 ps. Temperature and pressure were maintained at 300 K and 1 bar and controlled by modified Berendsen thermostat[25] and the Parrinello-Rahman[26] barostat. The LINCS[27] and Particle-Mesh Ewald[28] algorithms were applied to define bond parameters and long-range electrostatics. A radius cut-off of 12 Å was applied to short-range van der Waals and electrostatic terms. The analysis was performed using the GROMACS analysis tools (rms, distance, hbond, covar, and anaeig) and figures were created using PyMOL.[29]

Expression constructs

The pCMV6-CDK8-Myc-FLAG (Origene, RC212592), containing the human cDNA open reading frame of human *CDK8* (CDK8 WT) with C-terminal Myc-FLAG tags, was used for the functional analysis. The mutations in *CDK8* were generated by mutagenesis PCR using the primers listed in the Table S5 and following the Q5-Mutagenesis kit protocol (New England Biolabs). The previously described catalytically inactive CDK8 kinase-dead (p.Asp173Ala) mutant was used as control.[30-

[32] All constructs were verified by dideoxy-sequencing (R1, F2 and F3 primers in Table S5).

Cell culture, transfection and immunoblotting

The generation and full characterization of the SW620 *CDK8*/*CDK19* double-knockout cells will be described in detail elsewhere.[33] SW620 *CDK8*/*CDK19* double knockout and HEK293T cells were cultured in DMEM supplemented with 10% fetal bovine serum (FBS), L-glutamine, and penicillin-streptomycin at 37°C under 5% CO_2. For the functional assays transfections were performed using Lipofectamine 2000 (Thermo Fisher Scientific) according to the manufacturer's instructions and cells were processed after 24 h.

For immunoblotting, cells were collected and lysed in Cellytic M lysis buffer (Sigma-Aldrich) containing protease (Complete, Sigma-Aldrich) and phosphatase (PhosStop, Sigma-Aldrich) inhibitors. The lysates were clarified by centrifugation at 15,000 *g* for 15 min at 4°C and protein concentration was determined with the BCA Protein Assay Kit (Pierce, Thermo Scientific). All lysates were resolved on SDS-PAGE (Mini-Protean TGX 4-15% gradient gels, Biorad) and analyzed by Western blotting (WB) with specific antibodies. Primary antibodies used were anti-FLAG (F1804, Sigma-Aldrich), anti-CDK8 (4106S, Cell Signaling), anti-phospho-STAT1-Ser727 (8826, Cell Signaling), anti-STAT1 (total) (9176, Cell Signaling), anti-cyclin C (A301-989A-M, Bethyl Cambridge Bioscience) and anti-Myc (2278T, Cell Signaling). GAPDH was detected as a loading control using the HRP-conjugated anti-GAPDH (3683, Cell Signaling). For anti-phospho antibodies, the blocking reagent was 5% BSA in Tris-buffered saline, 0.1% Tween 20 (T-TBS) instead of non-fat milk. For quantitative analysis, captured bands were analyzed with the Image J software (National Institutes of Health, Bethesda, MD).

Immunoprecipitation, elution and thermal stability assay

We previously showed that ligand binding to the Mediator kinases determined by biochemical assays with recombinant CDK8/CDK19 and cyclin C is not always recapitulated in intact cells, suggesting that the other components of the kinase

module can influence CDK8 ligand-binding and activity.[30] Therefore we determined whether the CDK8 kinase domain substitutions had caused structural changes and/or affected the ability to bind ATP using whole kinase Module complexes isolated from cells. HEK293T cells were transiently transfected, harvested after 24 h, washed with phosphate-buffered saline (PBS) and lysed in 220 µl of immunoprecipitation lysis buffer (50 mM HEPES [pH 7.3], 150 mM NaCl, 1 mM $MgCl_2$, 1% Triton X100 [TX100]) containing protease (Complete, Sigma-Aldrich) and phosphatase (PhosStop, Sigma-Aldrich) inhibitors. The lysates were centrifuged at 15,000 *g* for 15 min at 4°C and the supernatant was transferred to a new tube. An aliquot of 20 µl was saved from the supernatant to analyze (by WB) the total cell lysate fraction (TCL). The remaining lysates (200 µl) were processed for immunoprecipitation. All the incubations and washes were done in a rotator at 4°C. Lysates were incubated with 1 µg of anti-FLAG or the mAb IgG1 isotype control (5415S, Cell Signaling) antibody for 4 h. Then, 50 µl of Dynabeads Protein G (Invitrogen) were added and incubated for 2 h. Beads containing the immunocomplexes were washed three times with washing buffer (20 mM HEPES [pH 7.3], 150 mM NaCl, 1 mM $MgCl_2$, 0.2% TX100, supplemented with protease and phosphatase inhibitors). The immunoprecipitates were released from the magnetic beads by adding 200 µl of washing buffer containing 200 µg/mL of the FLAG peptide (F3290, Sigma-Aldrich) and saved as eluted fraction. An aliquot of the eluted fraction was evaluated by WB to demonstrate the presence of the immunoprecipitated CDK8 protein and its specific interactor cyclin C. The remaining volume of the eluted fractions was used for the thermal stability assay. Each elution was diluted with kinase buffer (Cell Signaling Technology) and with or without ATP at 2 mM (Thermo Fisher Scientific). Each sample (with or without ATP) was divided into 12 equal aliquots and heated individually at different temperatures determined between 50 to 82°C for 3 min, using a gradient thermal cycler, and followed by 3 min cooling at 21°C. The samples were then centrifuged for 20 min at 4°C at 18,000 *g* to separate the soluble proteins from the protein precipitated/degraded. The supernatant was collected and the protein content was analyzed by capillary immunoassay following the manufacturer's protocol. The following antibodies were used when the capillary immunoassay system was employed: anti-CDK8 (4106S, Cell Signaling), anti-cyclin C (A301-989A, Bethyl Cambridge Bioscience), anti-MED12 (SC-5372, Santa-Cruz) and anti-MED13 (Abcam, Ab76923).

Statistical analysis

Graphs and statistical analyses were conducted with Prism 5 (GraphPad). The quantification of immunoblot analysis was performed from five independent experiments. P-values were calculated using one-way ANOVA with Dunnett's *post hoc* analysis. Values shown represent means ± standard error of the mean (SEM). In all figures, *$p \leq 0.05$, **$p \leq 0.01$, ***$p \leq 0.001$.

Web Resources

CGenFF webserver, https://cgenff.umaryland.edu/

GeneDx ClinVar submission page, http://www.ncbi.nlm.nih.gov/clinvar/submitters/26957/

MacKerell lab webserver, http://mackerell.umaryland.edu/

Supplemental References

1. Luo, X., Rosenfeld, J.A., Yamamoto, S., Harel, T., Zuo, Z., Hall, M., Wierenga, K.J., Pastore, M.T., Bartholomew, D., Delgado, M.R., et al. (2017). Clinically severe *CACNA1A* alleles affect synaptic function and neurodegeneration differentially. Plos Genet. *13*, e1006905.
2. Pinto, D., Delaby, E., Merico, D., Barbosa, M., Merikangas, A., Klei, L., Thiruvahindrapuram, B., Xu, X., Ziman, R., Wang, Z.Z., et al. (2014). Convergence of genes and cellular pathways dysregulated in autism spectrum disorders. Am. J. Hum. Genet. *94*, 677-694.
3. Frank, M., Albuisson, J., Ranque, B., Golmard, L., Mazzella, J.M., Bal-Theoleyre, L., Fauret, A.L., Mirault, T., Denarie, N., Mousseaux, E., et al. (2015). The type of variants at the *COL3A1* gene associates with the phenotype and severity of vascular Ehlers-Danlos syndrome. Eur. J. Hum. Genet. *23*, 1657-1664.
4. Wisniewski, S.A., and Trzeciak, W.H. (2012). A rare heterozygous *TRAF6* variant is associated with hypohidrotic ectodermal dysplasia. Brit. J. Dermatol. *166*, 1353-1356.
5. Hubshman, M.W., Basel-Vanagaite, L., Krauss, A., Konen, O., Levy, Y., Garty, B.Z., Smirin-Yosef, P., Maya, I., Lagovsky, I., Taub, E., et al. (2017). Homozygous deletion of *RAG1*, *RAG2* and 5' region *TRAF6* causes severe immune suppression and atypical osteopetrosis. Clin. Genet. *91*, 902-907.
6. Drmanac, R., Sparks, A.B., Callow, M.J., Halpern, A.L., Burns, N.L., Kermani, B.G., Carnevali, P., Nazarenko, I., Nilsen, G.B., Yeung, G., et al. (2010). Human genome sequencing using unchained base reads on self-assembling DNA nanoarrays. Science *327*, 78-81.
7. Gilissen, C., Hehir-Kwa, J.Y., Thung, D.T., van de Vorst, M., van Bon, B.W., Willemsen, M.H., Kwint, M., Janssen, I.M., Hoischen, A., Schenck, A., et al. (2014). Genome sequencing identifies major causes of severe intellectual disability. Nature *511*, 344-347.
8. Retterer, K., Juusola, J., Cho, M.T., Vitazka, P., Millan, F., Gibellini, F., Vertino-Bell, A., Smaoui, N., Neidich, J., Monaghan, K.G., et al. (2016). Clinical application of whole-exome sequencing across clinical indications. Genet. Med. *18*, 696-704.
9. Mcrae, J.F., Clayton, S., Fitzgerald, T.W., Kaplanis, J., Prigmore, E., Rajan, D., Sifrim, A., Aitken, S., Akawi, N., Alvi, M., et al. (2017). Prevalence and architecture of *de novo* mutations in developmental disorders. Nature *542*, 433-438.
10. Galbraith, M.D., Donner, A.J., and Espinosa, J.M. (2010). CDK8: a positive regulator of transcription. Transcription *1*, 4-12.
11. Molecular Operating Environment (MOE), 2015.10; Chemical Computing Group ULC, 1010 Sherbooke St. West, Suite #910, Montreal, QC, Canada, H3A 2R7, 2018.
12. Schneider, E.V., Bottcher, J., Huber, R., Maskos, K., and Neumann, L. (2013). Structure-kinetic relationship study of CDK8/CycC specific compounds. Proc. Natl. Acad. Sci. USA *110*, 8081-8086.

13. Bateman, A., Martin, M.J., O'Donovan, C., Magrane, M., Alpi, E., Antunes, R., Bely, B., Bingley, M., Bonilla, C., Britto, R., et al. (2017). UniProt: the universal protein knowledgebase. Nucleic Acids Res. *45*, D158-D169.
14. Baumli, S., Lolli, G., Lowe, E.D., Troiani, S., Rusconi, L., Bullock, A.N., Debreczeni, J.E., Knapp, S., and Johnson, L.N. (2008). The structure of P-TEFb (CDK9/cyclin T1), its complex with flavopiridol and regulation by phosphorylation. EMBO J. *27*, 1907-1918.
15. Schrödinger Release 2017-2: Maestro, Schrödinger, LLC, New York, NY, 2018.
16. Huang, J., Rauscher, S., Nawrocki, G., Ran, T., Feig, M., de Groot, B.L., Grubmuller, H., and MacKerell, A.D. (2017). CHARMM36m: an improved force field for folded and intrinsically disordered proteins. Nat. Methods *14*, 71-73.
17. Berendsen, H.J.C., Vanderspoel, D., and Vandrunen, R. (1995). GROMACS - a message-passing parallel molecular-dynamics implementation. Comput. Phys. Commun. *91*, 43-56.
18. Lindahl, E., Hess, B., and van der Spoel, D. (2001). GROMACS 3.0: a package for molecular simulation and trajectory analysis. J. Mol. Model. *7*, 306-317.
19. Van der Spoel, D., Lindahl, E., Hess, B., Groenhof, G., Mark, A.E., and Berendsen, H.J.C. (2005). GROMACS: fast, flexible, and free. J. Comput. Chem. *26*, 1701-1718.
20. Hess, B., Kutzner, C., van der Spoel, D., and Lindahl, E. (2008). GROMACS 4: algorithms for highly efficient, load-balanced, and scalable molecular simulation. J. Chem. Theory Comput. *4*, 435-447.
21. Pronk, S., Pall, S., Schulz, R., Larsson, P., Bjelkmar, P., Apostolov, R., Shirts, M.R., Smith, J.C., Kasson, P.M., van der Spoel, D., et al. (2013). GROMACS 4.5: a high-throughput and highly parallel open source molecular simulation toolkit. Bioinformatics *29*, 845-854.
22. Pall, S., Abraham, M.J., Kutzner, C., Hess, B., and Lindahl, E. (2015). Tackling exascale software challenges in molecular dynamics simulations with GROMACS. Lect. Notes Comput. Sci. *8759*, 3-27.
23. Abraham, M.J., Murtola, T., Schulz, R., Páll, S., Smith, J.C., Hess, B., and Lindahl, E. (2015). GROMACS: high performance molecular simulations through multi-level parallelism from laptops to supercomputers. SoftwareX *1-2*, 19-25.
24. Jorgensen, W.L., Chandrasekhar, J., Madura, J.D., Impey, R.W., and Klein, M.L. (1983). Comparison of simple potential functions for simulating liquid water. J. Chem. Phys. *79*, 926-935.
25. Berendsen, H.J.C., Postma, J.P.M., Vangunsteren, W.F., Dinola, A., and Haak, J.R. (1984). Molecular-Dynamics with coupling to an external bath. J. Chem. Phys. *81*, 3684-3690.
26. Parrinello, M., and Rahman, A. (1981). Polymorphic transitions in single-crystals - a new molecular-dynamics method. J. Appl. Phys. *52*, 7182-7190.
27. Hess, B., Bekker, H., Berendsen, H.J.C., and Fraaije, J.G.E.M. (1997). LINCS: a linear constraint solver for molecular simulations. J. Comput. Chem. *18*, 1463-1472.
28. Darden, T., York, D., and Pedersen, L. (1993). Particle mesh Ewald - An N Log(N) method for Ewald sums in large systems. J. Chem. Phys. *98*, 10089-10092.
29. PyMOL: the PyMOL molecular graphics system, Version 1.7.2.3 Schrödinger, LLC, New York, NY, 2018.
30. Dale, T., Clarke, P.A., Esdar, C., Waalboer, D., Adeniji-Popoola, O., Ortiz-Ruiz, M.J., Mallinger, A., Samant, R.S., Czodrowski, P., Musil, D., et al. (2015). A

selective chemical probe for exploring the role of CDK8 and CDK19 in human disease. Nat. Chem. Biol. *11*, 973-980.

31. Firestein, R., Bass, A.J., Kim, S.Y., Dunn, I.F., Silver, S.J., Guney, I., Freed, E., Ligon, A.H., Vena, N., Ogino, S., et al. (2008). *CDK8* is a colorectal cancer oncogene that regulates beta-catenin activity. Nature *455*, 547-551.
32. Gold, M.O., and Rice, A.P. (1998). Targeting of CDK8 to a promoter-proximal RNA element demonstrates catalysis-dependent activation of gene expression. Nucleic Acids Res. *26*, 3784-3788.
33. Ruiz, M.J.O., Popoola, O., Mallinger, A., Gowan, S., Court, W., Box, G., Valenti, M., Brandon, A.D.H., Te-Poele, R., Workman, P., et al. (2016). Elucidation of the different roles of CDK8 and CDK19 in colorectal cancer (CRC) using CRISPR gene editing technology. Cancer Res. *76(14 Suppl)*, Abstract nr 4355.
34. Guna, A., Butcher, N.J., and Bassett, A.S. (2015). Comparative mapping of the 22q11.2 deletion region and the potential of simple model organisms. J. Neurodev. Disord. *7*, 18.
35. Graham, J.M., Jr., and Schwartz, C.E. (2013). *MED12* related disorders. Am. J. Med. Genet. *161A*, 2734-2740.
36. Asadollahi, R., Zweier, M., Gogoll, L., Schiffmann, R., Sticht, H., Steindl, K., and Rauch, A. (2017). Genotype-phenotype evaluation of *MED13L* defects in the light of a novel truncating and a recurrent missense mutation. Eur. J. Med. Genet. *60*, 451-464.
37. Mukhopadhyay, A., Kramer, J.M., Merkx, G., Lugtenberg, D., Smeets, D.F., Oortveld, M.A.W., Blokland, E.A.W., Agrawal, J., Schenck, A., van Bokhoven, H., et al. (2010). *CDK19* is disrupted in a female patient with bilateral congenital retinal folds, microcephaly and mild mental retardation. Hum. Genet. *128*, 281-291.
38. Snijders Blok, L., Hiatt, S.M., Bowling, K.M., Prokop, J.W., Engel, K.L., Cochran, J.N., Bebin, E.M., Bijlsma, E.K., Ruivenkamp, C.A.L., Terhal, P., et al. (2018). *De novo* mutations in *MED13*, a component of the Mediator complex, are associated with a novel neurodevelopmental disorder. Hum. Genet. *137*, 375-388.
39. Vodopiutz, J., Schmook, M.T., Konstantopoulou, V., Plecko, B., Greber-Platzer, S., Creus, M., Seidl, R., and Janecke, A.R. (2015). *MED20* mutation associated with infantile basal ganglia degeneration and brain atrophy. Eur. J. Pediatr. *174*, 113-118.
40. Hashimoto, S., Boissel, S., Zarhrate, M., Rio, M., Munnich, A., Egly, J.M., and Colleaux, L. (2011). *MED23* mutation links intellectual disability to dysregulation of immediate early gene expression. Science *333*, 1161-1163.
41. Trehan, A., Brady, J.M., Maduro, V., Bone, W.P., Huang, Y., Golas, G.A., Kane, M.S., Lee, P.R., Thurm, A., Gropman, A.L., et al. (2015). *MED23*-associated intellectual disability in a non-consanguineous family. Am. J. Med. Genet. *167A*, 1374-1380.
42. Lionel, A.C., Monfared, N., Scherer, S.W., Marshall, C.R., and Mercimek-Mahmutoglu, S. (2016). *MED23*-associated refractory epilepsy successfully treated with the ketogenic diet. Am. J. Med. Genet. *170A*, 2421-2425.
43. Figueiredo, T., Melo, U.S., Pessoa, A.L.S., Nobrega, P.R., Kitajima, J.P., Correa, I., Zatz, M., Kok, F., and Santos, S. (2015). Homozygous missense mutation in *MED25* segregates with syndromic intellectual disability in a large consanguineous family. J. Med. Genet. *52*, 123-127.

44. Basel-Vanagaite, L., Smirin-Yosef, P., Essakow, J.L., Tzur, S., Lagovsky, I., Maya, I., Pasmanik-Chor, M., Yeheskel, A., Konen, O., Orenstein, N., et al. (2015). Homozygous *MED25* mutation implicated in eye-intellectual disability syndrome. Hum. Genet. *134*, 577-587.
45. Leal, A., Huehne, K., Bauer, F., Sticht, H., Berger, P., Suter, U., Morera, B., Del Valle, G., Lupski, J.R., Ekici, A., et al. (2009). Identification of the variant Ala335Val of MED25 as responsible for CMT2B2: molecular data, functional studies of the SH3 recognition motif and correlation between wild-type MED25 and PMP22 RNA levels in CMT1A animal models. Neurogenetics *10*, 275-287.
46. Leal, A., Bogantes-Ledezma, S., Ekici, A.B., Uebe, S., Thiel, C.T., Sticht, H., Berghoff, M., Berghoff, C., Morera, B., Meisterernst, M., et al. (2018). The polynucleotide kinase 3'-phosphatase gene (PNKP) is involved in Charcot-Marie-Tooth disease (CMT2B2) previously related to MED25. Neurogenetics *19*, 215–225.
47. Kaufmann, R., Straussberg, R., Mandel, H., Fattal-Valevski, A., Ben-Zeev, B., Naamati, A., Shaag, A., Zenvirt, S., Konen, O., Mimouni-Bloch, A., et al. (2010). Infantile cerebral and cerebellar atrophy is associated with a mutation in the *MED17* subunit of the transcription preinitiation Mediator complex. Am. J. Hum. Genet. *87*, 667-670.
48. Hirabayashi, S., Saitsu, H., and Matsumoto, N. (2016). Distinct but milder phenotypes with choreiform movements in siblings with compound heterozygous mutations in the transcription preinitiation Mediator complex subunit 17 (MED17). Brain Dev. *38*, 118-123.
49. Jeronimo, C., and Robert, F. (2017). The Mediator complex: at the nexus of DNA polymerase II transcription. Trends Cell Biol. *27*, 765-783.
